# Supplementary material for: A real-time regional model for COVID-19: Probabilistic situational awareness and forecasting
Source: PLoS Comput Biol. 2023 Jan 23;19(1):e1010860. doi: 10.1371/journal.pcbi.1010860 (PMC9894546; doi:10.1371/journal.pcbi.1010860)
Supplement: S1 Text — Additional supplementary results and methods. (PDF) [file pcbi.1010860.s001.pdf]

S1 Materials and Methods

S1.1 Population preserving mobility regularisation

When we used the calibrated model for prediction, we needed to consider inconsistencies in the total in- and outgoing flow for each region. Reasons for these inconsistencies were multiple: Customers turning off/on their mobile phone, the handset losing connection with the network, customers churning between different mobile operator's networks, etc. When there is an imbalance in the number of people entering and leaving a region, we will for long-term predictions risk that the population size in some regions grow out of control. In contrast, other regions may, in the worst case, become empty. Therefore, to preserve population at the region level, we regularised the mobility matrices. The regularised mobility matrix was the closest matrix to the observed mobility matrix, measured by mean squared error per entry, which satisfied the conservation constraint, namely that total incoming flow was equal to the total outgoing flow for each region. The obtained matrix entries were then rounded to the nearest integer.

Specifically, let X_{ij}^t be the element of the mobility matrix denoting the number of people moving from location i to location j between time t and time $t+1$. The conservation of people for each region requires Eq (S1) to hold:

$$X_{ii}^t + \sum_{j \neq i} X_{ji}^t = X_{ii}^{t+1} + \sum_{j \neq i} X_{ij}^{t+1}. \tag{S1}$$

Conserving the number of people in a location requires a balance in people not moving, people travelling out from the location and people travelling to it. In terms of the mobility matrices, this means the following: For a given location i and time t, all incoming transitions are summed up (column-wise), and this must equal the sum of outgoing transitions at time $t+1$ (row-wise).

Assume now that we want to simulate ahead in time, using only one mobility matrix for all times t. When we use the same matrix all the time, then Eq (S1) can be rewritten as Eq (S2) (where we drop t): For all sites i

$$\sum_{k:k \neq i} X_{i,k} = \sum_{k:k \neq i} X_{k,i}, \tag{S2}$$

which means that the matrix row-sums are equal to the column-sums. A symmetric matrix trivially fulfils this requirement. However, this is not the only possibility, and skewed mobility matrices are important due to commuting patterns.

We find the matrix Z that preserves the population of each region by solving the following Quadratic Programming problem:

$$\min_Z \sum_{i,j:i\neq j} (X_{ij} - Z_{ij})^2, \tag{S3}$$

subject to the linear constraints

$$\forall i \sum_{k:k\neq i} Z_{ik} = \sum_{k:k\neq i} Z_{ki}. \tag{S4}$$

For the predictions in this paper, we used regularised mobility data from the dates in Table A.

Prediction date	Regularisation date
09/01/2020	09/01/2020
10/01/2020	10/01/2020
11/01/2020	10/30/2020
03/01/2021	03/01/2021
04/01/2021	03/25/2021

Table A: Regularisation dates used in predictions. Dates are provided as mm/dd/yyyy.

S1.2 Hospitalisation risk and length of stay

To calculate the hospitalisation probability conditional on infection per age group, we started with the estimated hospitalisation probabilities in [1]. Note that the compartmental model did not have age compartments, as they would make the model computationally intractable. On the other hand, the probability of requiring hospitalisation was highly dependent on age. We corrected the hospital admission probabilities to account for age-dependencies in transmission. The disease burden did not just follow the age demography, and we needed to quantify the proportion of infected individuals per age group. To this end, we used the age distribution of the positive cases as a proxy for the age distribution of COVID-19 infections. Test data a commonly available data source. For Norway, we obtained test data from the MSIS registry. To account for a changing age profile of infected throughout

the pandemic, we used monthly estimates from May 1, 2020 in the national model, reported in Table B. In the regional model we used daily estimates.

Before May 1, 2020, we do not use the test data. Hence, because the demographic age profile in each county was available, we computed the county-wise probabilities of hospital admission as a mixture of the probabilities per age group in [1] prior to May 1, 2020, each multiplied with the proportion of the age group in the counties.

Compared to the population under study in [1], Norway has had a practice of not admitting elderly with COVID-19 living in nursing homes to hospitals. Therefore, we reduced the hospitalisation risk for the 80+ age group by 15% which corresponds to the percentage of non-nursing home residents . The total hospitalisation, $h_{i,t}$ for time, t and region i is given by

$$h_{i,t} = \sum_{j=0}^{9} \left[S_j * p_{ijt} \right], \tag{S5}$$

where p_{ijt} is the fraction of positive tests in age group j in region i at time t and S_j are the baseline probabilities from [1] corrected by the percentage of non-nursing home residents. The time-scale of t used was monthly in the national model and daily in the regional model (see section below).

When we predicted future hospitalisations in this paper, we did not use the future hospitalisation risks as it would require the use of impending positive test data that would not be available in a real-time setting. Instead, we used the latest available risk estimates for the relevant dates.

S1.2.1 Regional hospitalisation risks

The age distribution of the positive cases varied between counties and changed over time. We estimated the daily hospitalisation proportion of positive tests in each age group, county-wise and nationally, over a moving window over the last 28 days. We used the observed proportion of hospitalised cases in each age group. We added a small number in both the number of hospitalisations and the number of positive tests in each age group to avoid dividing by zero. Specifically, a pseudo count of 0.001 was added to the number of individuals hospitalised in each age group before dividing by the total number of individuals testing positive plus the number of age groups times 0.001. The country-specific proportions were adjusted using national data in those regions when few tested positive in a given period. This was done by calculating a weighted fraction: the county-specific

Table B: Hospitalisation probabilities when infected, adjusted for age, as estimated from test data, national values. Dates are provided as mm/dd/yyyy.

	Until 05/01/2020	Until 06/01/2020	Until 07/01/2020	Until 08/01/2021
0-9 years	0.0001	0.0005	0.001	0.00045
10 - 19 years	0.0004	0.001	0.001	0.001
20 - 29 years	0.006	0.007	0.0099	0.009
30 - 39 years	0.013	0.019	0.0091	0.017
40 - 49 years	0.018	0.015	0.015	0.016
50 - 59 years	0.043	0.032	0.016	0.032
60 - 69 years	0.059	0.031	0.025	0.039
70 - 79 years	0.086	0.048	0.038	0.032
80+ years	0.319	0.134	0.134	0.029
	Until 09/01/2020	Until 10/01/2020	Until 11/01/2020	Until 12/01/2020
0-9 years	0.0004	0.0004	0.0003	0.0006
10 - 19 years	0.001	0.001	0.001	0.0015
20 - 29 years	0.014	0.011	0.01	0.0072
30 - 39 years	0.013	0.015	0.016	0.014
40 - 49 years	0.013	0.014	0.017	0.016
50 - 59 years	0.025	0.024	0.027	0.028
60 - 69 years	0.015	0.030	0.031	0.036
70 - 79 years	0.029	0.033	0.032	0.037
80+ years	0.040	0.091	0.067	0.12
	Until 01/01/2021	Until 02/01/2021	Until 03/01/2021	From 03/01/2021
0-9 years	0.0006	0.0007	0.0014	0.0018
10 - 19 years	0.001	0.0013	0.0021	0.0026
20 - 29 years	0.007	0.0087	0.01	0.011
30 - 39 years	0.014	0.016	0.022	0.022
40 - 49 years	0.016	0.018	0.026	0.025
50 - 59 years	0.03	0.032	0.039	0.038
60 - 69 years	0.038	0.032	0.041	0.04
70 - 79 years	0.049	0.033	0.031	0.037
80+ years	0.14	0.14	0.064	0.043

fractions got a weight equal to the total number of positive cases during that period, while the national fraction got a weight of 50. Then this adjusted fraction of positive tests by age and county was used together with the age-specific probabilities of [1] to obtain a hospitalisation risk per age group and county.

S1.2.2 Change in hospitalisation risk due to new viral variants

Viral variants sometimes impact the hospitalisation risk. This was the case for the alpha variant (also called the B.1.1.7 variant)[2, 3]. The two first detected cases of the alpha variant imported to Norway occurred on December 27, 2020[4]. Since then, Norway experienced an increasing proportion of cases attributed to the alpha variant, which eventually became predominant. We adjusted for a gradually increasing hospitalisation risk by multiplying by a logistic increase in hospitalisation risk between January 1, 2021 and March 31, 2021, assuming a total increase of 60%, resulting in the following logistic function

$$B_{117}(t) = \frac{1000 \exp(-0.073t) + 1.6 \cdot 10 \exp(0.073t)}{1000 \exp(-0.073t) + 10 \exp(0.073t)} \tag{S6}$$

whose slope was estimated from the test data, and t denotes number of days from January 1st 2021.

The equation S7 shows how the regional hospitalisation probabilities are calculated from May 1st 2020 taking into account the take over curve of the B_{117} variant showed in S6. In the national model, we assumed an increase of 9% in January, 50% in February and 60% from March 2021 corresponding to the monthly averages of the logistic increase.

$$h_{i,t,117} = h_{i,t} * B_{117}(t) \tag{S7}$$

S1.2.3 Length of stay in hospital and time to hospital admission

To compute hospital incidence, we needed to know the time from symptom onset to hospital admission. Moreover, the predictions of hospital prevalence and the number of occupied intensive care treatment beds have been of interest, requiring estimates of the length of stay (LOS) in hospital for COVID-19 patients and the proportion needing intensive care treatment. The LOS in hospital for COVID-19 patients may change over time. We therefore allowed changepoints in the hospitalisation parameters. In addition, there may be regional differences, and we thus also included the possibility for separate parameters for certain counties.

These periods will here be called time-space periods. The parameters for the time-space periods were estimated separately.

The aim was to estimate distributions for the time from symptom onset to hospitalisation, the LOS for patients with and without ventilator treatment, and for the proportion of hospitalised cases needing ventilator treatment. The LOS for patients needing ventilator treatment was divided into three periods: before, during and after ventilator treatment to obtain estimates of the number of required ventilators per day. For patients with more than one COVID-19 hospitalisation episode, the LOS was set equal to the sum of all admission periods.

We used a proportional hazard Cox model to estimate the parameters for each time-space period, assuming right censoring in time. The survival curves were estimated with time-space periods as an explanatory variable, using the *survfit* function in the R-package *survival*. Specifically, one minus the cumulative distribution function of the negative binomial distributions was fitted to each survival curve by minimising the sum of squared errors, using the function *optim* in R.

To estimate the hospitalisation parameters we used individual-level data from the Norwegian Surveillance System for Communicable Diseases (MSIS) and the Norwegian Intensive Care and Pandemic Registry (NIPaR)[5] as explained in the main text. By April 12, 2021, NIPaR included 4.248 hospitalised individuals, of whom 735 needed mechanical respirator treatment, while MSIS contained 103.461 positive test results. We include three changepoints, dividing the data into four periods – before June 2020, June 2020-December 2020, January 2021-February 2021, and March 2020-April 12, 2021.

Except for the county of Oslo, there were no significant differences in the regional COVID-19 hospitalisation parameters. The hospitals in Oslo increased the use of mechanical ventilators from January 2021. Therefore, we obtained separate estimates for Oslo in the last two time periods. When estimating the time spent under ventilator treatment, we merged the periods January 2021-February 2021 and March 2021-April 12, 2021, to reduce the level of censoring. In a separate project, we are trying to find the causes of these changes, see also [6]. Figs A, B, C, D and E show for each time-space period a histogram of time to event together with an estimated negative binomial distribution fitting the data in red. The parameters of the negative binomial distribution (mu and size) together with the number of patients (N) in the data are shown in the upper right corner of each panel.

S1.2.4 Time from symptom onset to hospitalisation

When estimating time from symptom onset to the first hospitalisation, patients with symptom onset date equal to the COVID-19 test date were disregarded. In Norway, this registration practice is common in situations where symptom onset is unknown. We assumed that these cases were missing at random and hence did not introduce bias. All patients included in this analysis were hospitalised, so no right censoring was needed for this calculation. Fig A shows the real data and the fitted distribution in red.

S1.2.5 Time from hospitalisation to discharge

The time from hospitalisation to discharge was estimated separately for two groups, those who required ventilator treatment and those who did not. Right censoring occurred in the last time-space period from March 2021; 15 and 65 patients from Oslo and the rest of Norway, respectively, were still in hospital at the end of that period, out of a total of 476 patients from Oslo and 960 from the rest of Norway. The number of patients included in each time-space period is shown in Fig B together with the real data and the fitted distribution in red.

For those needing mechanical ventilator treatment, we considered three time intervals: time from admission to start of ventilator treatment (Fig C), time on ventilator (Fig D) and time to discharge after ventilator treatment (Fig E). Both the estimation of the distribution of the time on ventilator and time after ventilator treatment included censoring events in the last period from January 2021. 13 patients from Oslo and 23 patients from the rest of Norway were censored when estimating time on ventilator, out of a total of 132 patients from Oslo and 207 patients from the rest of Norway. For the time after ventilator treatment, the censored patients were 17 and 28 for the same periods, out of a total of 120 for Oslo and 170 for the rest of Norway.

The proportion of patients needing ventilator treatment has changed over time. From the beginning until June 2020, 0.149 (163 out of 1092) of patients required ventilator treatment. Between June 2020-December 2020 the proportion decreased to 0.098 (133 out of 1363). In the last period, January 2021-April 12 2021, 0.156 (121 out of 774) of patients in Oslo required ventilators, in the rest of Norway the proportion was 0.121 (191 out of 1581).

S1.3 Parameter assumptions

A lot is uncertain about the natural history of SARS-CoV-2. Hence, there are many unknown parameters in the model that our method estimates (or calibrates) from data. We calibrate the amplification factor and the transmission parameters β_t^i, which are directly related to the reproductive numbers. We also calibrate the parameters π_0 and π_1 in the model for the detection of positive tests, and the delay d between entering the infectious symptomatic or asymptomatic class and a positive test. As described above, we estimate the hospitalisation parameters from individual-level data on hospitalised cases.

The remaining parameters related to the natural history of COVID-19 are assigned fixed values or distributions based on values found in the literature. All the parameter choices and values are provided in Table C.

Table C: Parameter assumptions

Assumptions	Mean	Distribution	Inspirational reference
**Model parameters**			
Exposed period ($1/\lambda_1$)	3 days	Exponential	[7]
Presymptomatic period ($1/\lambda_2$)	2 days	Exponential	[7]
Symptomatic infectious period ($1/\gamma$)	5 days	Exponential	[7]
Asymptomatic, infectious period ($1/\gamma$)	5 days	Exponential	[7]
Infectiousness asympt. (r_{I_a})	0.1	Fixed	[7]
Infectiousness presymp. (r_{E_2})	1.25	Fixed	[7]
Probability asymptomatic (p_a)	0.4	Fixed	[7]
**Healthcare**			
% symptomatic and asymptomatic infections requiring hospitalisation:<br>0-9 years<br>10 - 19 years<br>20 - 29 years<br>30 - 39 years<br>40 - 49 years<br>50 - 59 years<br>60 - 69 years<br>70 - 79 years<br>80+ years	 <br>0.1%<br>0.1%<br>0.5%<br>1.1%<br>1.4%<br>2.9%<br>5.8%<br>9.3%<br>22.3%	Fixed	[1]<br>corrected for: proportion of elderly living in elderly homes in Norway (last two age groups), and age-profile in laboratory-confirmed cases since May 1, 2020. Corrected values available in table B.
Overall hospitalisation risk	2.26%	Fixed	[1]<br>(adapted to Norwegian demography)

S1.4 Regional baseline model

To compare the performance of the regional model with a regional baseline or null model, we implemented a simple baseline model. The model uses the last 21

days of the data (hospitalisation or confirmed cases) and fits a negative binomial regression model with only an intercept term. The regression model is then used to make predictions for the next 21 days as the in the other models. To ensure some variation in the data if there were no hospitalisations during the training period, we added one hospitalisation on the first day of the training data.

S2 Supplementary results

S2.1 Estimated national reproduction numbers

In addition to Table 2 in the main text, we also provide a visualisation of the estimated national reproduction numbers in Fig F.

S2.2 Validation results

The validation scores are provided in Table D.

Table D: Validation results comparing the regional, national and regional baseline model.

	National			Regional			Regional baseline		
	Week 1	Week 2	Week 3	Week 1	Week 2	Week 3	Week 1	Week 2	Week 3
**Hospital**									
County Energy Score	25.62	30.81	31.51	13.78	16.29	18.97	15.23	26.08	35.70
National CRPS	4.52	16.46	32.12	21.75	23.03	18.48	18.92	41.77	59.63
Coverage 50% intervals	0.44	0.36	0.35	0.64	0.45	0.56	0.58	0.47	0.51
Coverage 95% intervals	0.62	0.56	0.62	0.85	0.85	0.80	0.87	0.78	0.67
**Confirmed Cases**									
County Energy Score	754.81	839.09	860.36	380.28	529.86	704.24	440.22	816.90	1032.77
National CRPS	547.20	784.84	893.63	674.01	612.28	644.91	919.10	1453.29	1791.04
Coverage 50% intervals	0.13	0.22	0.22	0.24	0.25	0.18	0.11	0.29	0.25
Coverage 95% intervals	0.36	0.45	0.51	0.55	0.64	0.64	0.58	0.56	0.56

S2.3 Fit to test and hospitalisation data

When estimating the transmissibility, we choose to use both the hospitalisation data from the beginning of the epidemic and the test data from May 2020, as both contain information.

S2.3.1 Fit to data up to April 2021

The fit to the regional hospitalisation data until April 2021, along with three-weeks ahead predictions, is provided in Figs G-H, for the model allowing region-specific transmissibilities. The fit to the test data is provided in Figs I-J. Note that the test data are more volatile than the hospitalisation incidence due to flare-ups of local outbreaks that become controlled. For this reason, test data are not well captured by the constant step-function in the transmissibility unless the dates for the changepoints are tailored to the dates of the outbreaks (see for example Agder).

However, the test data do contain information on the transmissibility, in particular for the more recent days. We note that for the first days of prediction, our hospital incidence predictions seem to cover the real data that are later observed, but that we start overpredicting over time. This is due to local restrictions implemented in Oslo on March 17, 2021, and national restrictions implemented on March 25, 2021, which have reduction effects that we have not captured with our most recently estimated effective reproduction number, which is active from March 2, 2021. This is particularly clear in the fit to the largest region in Norway, Viken, and in the national aggregate.

Note that we do not capture all the uncertainty in the test data. This is likely because we calibrate to a 7-days-backwards moving average of the daily test data. Moreover, the binomial assumption could be too simple, and a beta-binomial distribution would allow more variance, at the cost of an additional parameter.

S2.3.2 Fit to data up to March 2021

We include also the fit to the test data and hospitalisation data for the calibration up to March 2021. Here, no changepoints later than February 8, 2021 were used. The fit to the regional hospitalisation data until March 1, along with three-weeks ahead predictions, is provided in Figs K-L, for the model allowing region-specific transmissibilities. The fit to the test data is provided in Figs M-N. The predictions of future hospitalisations are better here than for April 1, 2021, as the intervention measures implemented later in March take more time before the effect shows up in the data. We note however that we are underestimating the hospitalisation a bit, in particular in Viken.

We repeat the calibration up to March 1, 2021 using only hospitalisation data. The fit to the hospital incidence is provided in Figs O-P. If we consider Viken, we note that the hospitalisation predictions are better in this setting when only calibrating to the hospitalisation data, than when we calibrate to both the hospitalisa-

tion data and the test data, when we underpredicted the hospitalisation incidence (Figs K-L). However, the variance is also larger.

S2.3.3 Fit to data up to November 1, 2020

We include also the fit to the test data and hospitalisation data for the calibration up to November 1, 2020. Here, no changepoints later than October 1, 2020 were used. The fit to the regional hospitalisation data until November 1, 2020, along with three-weeks ahead predictions, is provided in Figs Q-R, for the model allowing region-specific transmissibilities. The fit to the test data is provided in Figs S-T. We note that for the future hospitalisations, we are underestimating the cases in Oslo.

S2.3.4 Fit to data up to October 1, 2020

We include also the fit to the test data and hospitalisation data for the calibration up to October 1, 2020. Here, no changepoints later than September 1, 2020 were used. The fit to the regional hospitalisation data until October 1, 2020, along with three-weeks ahead predictions, is provided in Figs U-V, for the model allowing region-specific transmissibilities. The fit to the test data is provided in Figs W-X. We note the low level of hospitalisation incidence in Norway in October 2020.

S2.3.5 Fit to data up to September 1, 2020

We include also the fit to the test data and hospitalisation data for the calibration up to September 1, 2020. Here, no changepoints later than August 1, 2020 were used. The fit to the regional hospitalisation data until September 1, 2020, along with three-weeks ahead predictions, is provided in Figs Y-Z, for the model allowing region-specific transmissibilities. The fit to the test data is provided in Figs AA-AB. In September 2020, there were few COVID-19 cases in Norway.

S2.3.6 Fit to data up to August 1, 2020

We include also the fit to the test data and hospitalisation data for the regional calibration up to August 1, 2020. This is the calibration of the past which is considered fixed in the later regional calibrations. Here, no changepoints later than July 1, 2020 were used. The fit to the regional hospitalisation data until August 1, 2020 is provided in Figs AC-AD, for the model allowing region-specific transmissibilities. The fit to the test data is provided in Figs AE-AF. We note

Calibration	Sep 1st-2020	Oct 1st-2020	
Period	08/01-08/31	08/01-08/16	08/17-09/30
Oslo	0.88 (0.73 - 1.03)	0.58 (0.22 - 0.99)	1.28 (1.16 - 1.39)
Rogaland	0.19 (0.04 - 0.34)	0.15 (0.01 - 0.38)	0.7 (0.37 - 0.97)
Nordland	0.16 (0.02 - 0.37)	0.28 (0.03 - 0.67)	0.29 (0.03 - 0.66)
Møre og Romsdal	0.84 (0.42 - 1.23)	1.4 (0.31 - 2.5)	0.21 (0.01 - 0.5)
Viken	0.39 (0.29 - 0.49)	0.32 (0.09 - 0.53)	0.88 (0.77 - 0.99)
Innlandet	0.42 (0.17 - 0.64)	0.36 (0.03 - 0.82)	0.48 (0.1 - 0.82)
Vestfold og Telemark	0.15 (0.01 - 0.37)	0.32 (0.02 - 0.82)	0.12 (0.01 - 0.35)
Agder	0.27 (0.08 - 0.46)	0.18 (0.01 - 0.46)	0.55 (0.11 - 0.93)
Vestland	0.95 (0.79 - 1.12)	0.82 (0.29 - 1.28)	1.1 (0.99 - 1.21)
Trøndelag	0.36 (0.14 - 0.58)	0.79 (0.21 - 1.43)	0.11 (0.01 - 0.25)
Troms og Finmark	0.21 (0.03 - 0.44)	0.56 (0.07 - 1.15)	0.09 (0.01 - 0.21)
Calibration	Nov 1st-2020		
Period	08/01-08/31	09/01-09/30	10/01-10/31
Oslo	0.97 (0.62 - 1.32)	1.25 (0.97 - 1.52)	1.46 (1.22 - 1.68)
Rogaland	0.25 (0.02 - 0.51)	1.03 (0.7 - 1.29)	
Nordland	0.24 (0.02 - 0.61)	0.75 (0.06 - 1.65)	1.18 (0.15 - 2.08)
Møre og Romsdal	0.4 (0.04 - 0.91)	0.63 (0.1 - 1.17)	
Viken	0.46 (0.11 - 0.74)	0.88 (0.51 - 1.23)	1.49 (1.18 - 1.83)
Innlandet	0.23 (0.01 - 0.71)	1.14 (0.79 - 1.46)	
Vestfold og Telemark	0.25 (0.02 - 0.7)	0.62 (0.14 - 1.13)	
Agder	0.16 (0.01 - 0.45)	1.04 (0.35 - 1.69)	0.82 (0.11 - 1.68)
Vestland	0.92 (0.6 - 1.27)	0.97 (0.65 - 1.25)	1.51 (1.18 - 1.88)
Trøndelag	0.19 (0.02 - 0.43)	0.93 (0.52 - 1.24)	
Troms og Finmark	0.25 (0.02 - 0.58)	0.87 (0.45 - 1.22)	

Table E: Estimated regional reproductive numbers. Calibration from August 1 ,2020. Median and 95 % CIs.

the low disease level during summer. The corresponding estimated reproduction numbers are provided in Tables E, F,G.

S2.4 Changepoint selection

We here present our calibration of the changepoint corresponding to the first lockdown in Norway. We calibrated the timing of the first changepoint by comparing the fit with a changepoint on March 11, 12, 13, 14, 15, and 16. This was done because the interventions were implemented gradually. The fit for the different changepoints is provided in Fig AG. The fit was clearly best when using March 15 or March 16 as a changepoint. The differences between the two were minimal, but March 15 gave a slightly better fit, and we thus chose to use March 15 as the changepoint.

Calibration	March 1st-2021 (test and hospitalizations)		March 1st-2021 (hospitalizations)	
Period	01/04-02/04	02/05-03/01	01/04-02/04	02/05-03/01
Oslo	0.78 (0.69 - 0.88)	1.67 (1.44 - 1.91)	0.71 (0.54 - 0.87)	1.77 (1.22 - 2.33)
Rogaland	0.3 (0.16 - 0.43)	0.85 (0.13 - 1.65)	0.14 (0.01 - 0.38)	0.84 (0.06 - 2.15)
Nordland	0.42 (0.12 - 0.68)	0.73 (0.1 - 1.51)	0.29 (0.02 - 0.76)	0.78 (0.04 - 2)
Møre og Romsdal	0.69 (0.35 - 1.01)	1.3 (0.53 - 1.96)	0.32 (0.02 - 0.85)	0.88 (0.09 - 2.29)
Viken	0.84 (0.76 - 0.92)	1.22 (1 - 1.41)	0.71 (0.55 - 0.86)	1.57 (1.1 - 2.05)
Innlandet	0.44 (0.18 - 0.66)	0.4 (0.04 - 0.96)	0.19 (0.01 - 0.55)	0.65 (0.05 - 1.64)
Vestfold og Telemark	0.73 (0.51 - 0.91)	1.01 (0.57 - 1.46)	0.32 (0.02 - 0.72)	1.17 (0.12 - 2.5)
Agder	0.88 (0.61 - 1.1)	1.39 (0.96 - 1.85)	0.31 (0.03 - 0.78)	0.7 (0.05 - 1.86)
Vestland	0.94 (0.78 - 1.11)	0.5 (0.12 - 0.95)	1.02 (0.6 - 1.38)	0.39 (0.03 - 1.03)
Trøndelag	0.33 (0.16 - 0.51)	0.58 (0.09 - 1.17)	0.2 (0.02 - 0.52)	0.75 (0.04 - 1.92)
Troms og Finmark	0.14 (0.01 - 0.37)	0.77 (0.09 - 1.71)	0.27 (0.02 - 0.76)	0.73 (0.05 - 1.9)

Table F: Estimated regional reproductive numbers. Calibration from January 4, 2021 up to March 1, 2021. Median and 95 % CIs.

	April 1st-2021			
	R_1	Period	R_2	Period
Oslo	0.88 (0.81 - 0.95)	01/04-02/04	1.56 (1.49 - 1.64)	02/05-03/13
Rogaland	0.25 (0.1 - 0.39)	01/04-01/31	1.29 (0.63 - 1.8)	02/01-02/19
Nordland	0.36 (0.13 - 0.59)	01/04-02/04	0.36 (0.03 - 0.87)	02/05-03/08
Møre og Romsdal	0.52 (0.18 - 0.87)	01/04-02/04	1.16 (0.26 - 2.09)	02/05-02/19
Viken	0.86 (0.78 - 0.94)	01/04-02/04	1.13 (0.93 - 1.34)	02/05-02/21
Innlandet	0.23 (0.03 - 0.48)	01/04-02/21	0.81 (0.29 - 1.38)	02/22-03/08
Vestfold og Telemark	0.57 (0.32 - 0.83)	01/04-02/04	1.6 (1 - 2.22)	02/05-02/21
Agder	0.67 (0.45 - 0.91)	01/04-02/19	0.85 (0.23 - 1.45)	02/20-03/08
Vestland	0.65 (0.34 - 0.98)	01/04-02/04	0.83 (0.41 - 1.21)	02/05-03/08
Trøndelag	0.45 (0.23 - 0.65)	01/04-02/04	0.2 (0.01 - 0.59)	02/05-03/08
Troms og Finmark	0.33 (0.09 - 0.57)	01/04-02/04	0.55 (0.04 - 1.5)	02/05-02/19
	R_3	Period	R_4	Period
Oslo	0.84 (0.72 - 0.99)	03/14-04/01	-	
Rogaland	1.84 (1.19 - 2.7)	02/20-03/08	1.18 (0.79 - 1.56)	03/09-04/01
Nordland	1.72 (0.49 - 3.02)	03/09-04/01	-	
Møre og Romsdal	1.22 (0.51 - 1.89)	02/20-03/08	0.65 (0.26 - 1.02)	03/09-04/01
Viken	1.61 (1.43 - 1.79)	02/22-03/08	1.19 (1.08 - 1.29)	03/09-04/01
Innlandet	0.57 (0.16 - 1.01)	03/09-04/01	-	
Vestfold og Telemark	1.73 (1.26 - 2.27)	02/22-03/08	0.55 (0.26 - 0.85)	03/09-04/01
Agder	0.29 (0.03 - 0.84)	03/09-04/01	-	
Vestland	0.94 (0.28 - 1.68)	03/09-04/01	-	
Trøndelag	0.53 (0.05 - 1.34)	03/09-04/01	-	
Troms og Finmark	1.07 (0.25 - 1.83)	02/20-03/08	0.55 (0.09 - 1.05)	03/09-04/01

Table G: Estimated regional reproductive numbers. Calibration from January 4, 2021 up to April 1, 2021. Median and 95 % CIs.

S2.5 Computational time and convergence

In particular for the regional calibration, the parameter space is very high-dimensional and correlated in both time and space, which makes the calibration exercise difficult. The calibration took one week running on 1400 cores. The run-time for a single sample is in seconds, what mainly takes time is to find parameters which improve the error, not the disease spread simulation itself.

Fig AH shows a typical example of the evolution of the estimated distributions of the parameters throughout the calibration rounds.

S2.6 Policy compliance and reduced mobility

The mobility data are indicators for compliance with mobility reducing policies, like travel restrictions and working from home. Fig AI shows the daily number of trips out from each of Norway's counties. There was a clear reduction in mobility during the March 2020 lockdown of approximately 50%. Thereafter, mobility increased steadily until the summer. In July, geographic differences in mobility corresponded to summer vacations. Later in 2020, Norway experienced high, regionally varying mobility, with peaks around vacations. Late 2020 and early 2021, mobility decreased again during the second and third waves. The systematic mobility drop on weekends indicated commuting to work even though teleworking was recommended. We compared mobility to March 1, 2020, as we do not have mobility data before the pandemic, which also means that we cannot compare general seasonalities in mobility volume.

For some counties (e.g., Troms og Finnmark), the mobility recovered to the levels prior to the first lockdown, while this was not the case for others (Oslo and Viken).

S2.7 Sensitivity to mobility implementation

We performed a sensitivity analysis to ensure robustness to the choice of mobility rule for moving people between regions in the model. To allow for comparison of the spread of the pandemic between different regions, we utilised a national model for this analysis, assuming common fixed reproductive values for each region.

We ran the model from February 17, 2020 to October 18, 2021 with two different R_0 values of 1.1 and 1.5 in order to understand the importance of the mobility rule with different infectivity levels in the pandemic. We use the actual imported cases in the simulations. To understand the importance of these mobility rules in a

more interpretable scenario of limited foreign seeding, we in addition ran simulations with each rule starting with 100 cases in Oslo on day 1 (February 17, 2020) and no seeding thereafter.

We ran 100 simulations of the model assuming different mobility rules, when considering the problem of how to move individuals from location A to location B. We denote by "visitors" the individuals who are present in B and have A as their home location. We denote by "host" the individuals who are present in location B and have B as their home location. We denote by "others" the individuals who are present in location B and have other locations than A or B as their home location. The rule applied in the paper will therefore hereafter be referred to as $Visitors-Host-Others$ (VHO), as we first send the individuals here defined as "visitors", then the individuals here defined as "host", and lastly the individuals here defined as "others". If there are not enough individuals present in location B, we add susceptibles to the host population of B. As a control experiment, we also ran another set of 100 simulations for the rule applied in the paper, hereafter referred to as $Visitors-Host-Others2$ $(VHO2)$. Hence, VHO2 is a repetition of VHO to assess the variability between two runs with the same rule. We additionally explored four other mobility rules defined as follows:

1. $Random$ (R): We randomly sample from all people in a location, without distinguishing between visitors and host population. Then, we add extra people if necessary.

2. $Visitors - Random$ (VR): We send back visitors from location B first, and then randomly sample from the remaining individuals who are present in A and add extra people if necessary.

3. $Visitors - Others - Host$ (VOH): We send back visitors from location B first, and then select from the other visitors to the location. Finally, we select from the host population and add extra people if necessary.

4. $VHORegularised$ (Reg): We used the VHO mobility rule applied in the paper to move people between two locations, but we used regularised matrices instead of the original origin-destination matrices. This ensures that we do not have to add extra people into the model at any point.

To better understand the effect of regularisation of the mobility matrices, we additionally quantified the absolute daily difference between inter-county mobility in the regularised matrices and the original mobility matrices from February 17, 2020 to September 27, 2021 (Fig AM). We observe that the differences are generally higher during the summer and autumn seasons than the winter and spring seasons.

We determined the date and size of the peak, i.e. the maximum number of

infected cases on a national and county level for each of the simulations. We define infected cases as the number of infectious, infectious asymptomatic, and pre-symptomatic infectious cases who are residents in the county of interest and are thus considered a part of the host population of that county. We then plotted the mean of the peak size for the 100 simulations nationally, (Fig AJ), and county-wise (Fig AK).We also computed the mean number of days until 1% of each county's resident population was infected (Fig AL).

We only conducted regional analyses for a scenario with limited foreign seeding, as it is easier to interpret the effect of the mobility rules without additional seeding throughout the pandemic.

Using the metrics of the national and county-specific peak sizes and time to 1% infected, we observe very few statistically significant differences between the rules. However, the random rule (R) has a higher national peak that is almost statistically significant, which is expected, as infections tend to spread more with random movement of people [8]. Though, we do not find this behaviour in the county-specific analysis. In Oslo county, the peak is lower when moving visitors and hosts randomly (VR) compared to those obtained with the other mobility rules. This finding may be explained by the strong asymmetry in the matrices from this particular county. With the regularised matrices (Reg), the peaks tend to be lower than with the other rules, though not significantly. One reason for this observation is that no extra people are added to the model, which may give rise to fewer infected people at the peaks. Given that the differences between the results from the different mobility rules were almost always statistically insignificant, the sensitivity analysis indicates that our simulations are robust to the choice of mobility rule.

S2.8 Effect of mobility on exponential growth rates

We illustrate the effect of mobility on the exponential growth by simulating a set of scenarios where we increase the reproduction number in Oslo county while setting the reproduction number in every other county equal to one. We simulate these scenarios with and without mobility included in the model. In Fig AN we see that when the difference in reproduction number is large between counties and with substantial mobility, there is a significant decrease in the exponential growth rate for a given reproduction number when we include mobility. When there are large differences between reproduction numbers, one could easily underestimate the reproduction number in the county with higher transmission by 0.5. The effect is driven by a large number of infected persons travelling to other counties, while

few infected people travel back to Oslo since the prevalence of infected are much lower in other counties.

S2.9 Comparison of regional reproduction numbers with alternative approaches

A much simpler approach to estimating the (regional) reproduction numbers based on case data is the commonly used method implemented in the R-package EpiNow2 [9]. We run EpiNow2 with the same changepoints as in the regional model. We use an incubation period based on [10] and a generation time with mean 7 days standard deviation of 3.5 days matching the generation time in the regional model.

Using a method like EpiNow2, we lose a lot of details, like for example changes in imported cases over time, the connection between the different regions over time due to mobility, and changes in testing criteria and capacity. It is also not straightforward to include multiple case data sources at the same time, and hence we show the results from EpiNow2 when calibrated to the number of positive tests only. The resulting reproduction numbers for Oslo and Viken are provided in Fig AO and AP, respectively.

In general, the overall patterns are the same. However, we see that the discrepancy is large for the early part of the pandemic. As discussed earlier, this is likely because the test data were not reliable in this period due to frequent changes in testing criteria and capacity. For Oslo, the estimates from our model framework are in general higher, as we have an outflow of infectious individuals from Oslo due to mobility (see also the previous section). The opposite effect is found for Viken. For both Viken and Oslo, we note that the estimates for early autumn 2020 from EpiNow2 are higher than our estimates, which is likely due to a large number of imported cases coinciding with border reopening, which is not taken into account in EpiNow2.

S2.10 Sensitivity analysis of the probability of asymptomatic infection

In order to evaluate whether the estimated reproduction numbers were robust to the probability of developing asymptomatic infection (p_a), which was set to 0.4 in the main analyses, we re-calibrated the model with a national reproduction number, with data until April 1, 2021 (like in section ”The National picture” in the main text) with $p_a = 0.3$ and $p_a = 0.5$. Fig AQ presents a comparison of the

Table H: **Synthetic reproductive numbers**

	R_0 until 03/14	R_1 03/15 - 04/19	R_2 04/20 - 07/24
Oslo	5.52	1.3	0.5
Rest	3.44	1.1	0.7
	R_4 07/25 - 08/16	R_5 From 08/17	
Oslo	1.6	0.4	
Rest	1.3	0.6	

reproductive numbers obtained in the three different calibrations. The posteriors of the estimated reproductive numbers are very similar, and hence robust to the exact assumption of asymptomatic proportion within the analysed range.

S2.11 Verification test for Split-SMC-ABC

In order to study the performance of the Split-SMC-ABC calibration method suggested in this paper, we have performed one simple test with synthetic data, generated with a certain set of known, true underlying parameters.

We generated synthetic data (Fig AR) from the beginning of the pandemic up until October 1, 2020 assuming that the reproductive number was the same in all regions except Oslo. We used four changepoints, resulting in a total of ten reproductive numbers: five for Oslo and five for the other counties (see Table H for more details). The remaining parameters of the model (π_0,π_1, delay between infectious and testing) and the amplification factor, were assumed fixed.

In the Split-SMC-ABC, we applied a split on July 25, 2020, running the first calibration using data 15 days into the future, as in the main analysis. The assumed prior distributions were the same as those used in the main analysis.

As shown in Fig AS and Fig AT, both the presplit and postsplit stages converged asymptotically to the ground truth values for most parameters. One exception is R_1 in Oslo, where the method seems to converge, but to a number which is somewhat higher than the ground truth. Note, however, that we are calibrating to one single realisation of the stochastic disease spread, and hence it might be that the underlying truth is not the best fitting parameter.

S3 Implementation

The main model is implemented in the R-package spread `https://github.com/sykdomspulsen-org/spread`. We provide the implementation code for the extraction of the hospitalisation parameters, the code for fitting the split-ABC algorithm and for the validation of the forecasts in `https://github.com/folkehelseinstituttet/realtime-regional-model`

References

[1] Henrik Salje, Cécile Tran Kiem, Noémie Lefrancq, Noémie Courtejoie, Paolo Bosetti, Juliette Paireau, Alessio Andronico, Nathanaël Hozé, Jehanne Richet, Claire-Lise Dubost, Yann Le Strat, Justin Lessler, Daniel Levy-Bruhl, Arnaud Fontanet, Lulla Opatowski, Pierre-Yves Boelle, and Simon Cauchemez. Estimating the burden of SARS-CoV-2 in France. *Science*, (eabc3517), 2020.

[2] Peter Bager, Jan Wohlfahrt, Jannik Fonager, Morten Rasmussen, Mads Albertsen, Thomas Yssing Michaelsen, Camilla Holten Møller, Steen Ethelberg, Rebecca Legarth, Mia Sarah Fischer Button, Sophie Madeleine Gubbels, Marianne Voldstedlund, Kåre Mølbak, Robert Leo Skov, Anders Fomsgaard, Tyra Grove Krause, and The Danish Covid-19 Genome Consortium. Risk of hospitalisation associated with infection with sars-cov-2 lineage b. 1.1. 7 in denmark: an observational cohort study. *The Lancet Infectious Diseases*, 2021.

[3] Robert Challen, Ellen Brooks-Pollock, Jonathan M Read, Louise Dyson, Krasimira Tsaneva-Atanasova, and Leon Danon. Risk of mortality in patients infected with sars-cov-2 variant of concern 202012/1: matched cohort study. *bmj*, 372, 2021.

[4] Folkehelseinstituttet. COVID-19-EPIDEMIEN: Nye varianter av SARS-CoV-2: kunnskap, risiko og respons, 2020. [Online; accessed 08-March-2021].

[5] Helse Bergen–Haukeland Universitetssjukehus. The Norwegian Pandemic Registry, 2020. [Online; accessed 12-May-2021].

[6] Robert Whittaker, Anja Bråthen Kristofferson, Elina Seppälä, Beatriz Valcarcel Salamanca, Lamprini Veneti, Margrethe Larsdatter Storm, Håkon Bøås, Nina Aasand, Umaer Naseer, Karoline Bragstad, Olav Hungnes, Reidar Kvåle, Karan Golestani, Siri Feruglio, Line Vold, Karin Nygård, and Eirik Alnes Buanes. Trajectories of hospitalisation for patients infected with sars-cov-2 variant b. 1.1. 7 in norway, december 2020–april 2021. *medRxiv*, 2021.

[7] Luca Ferretti, Chris Wymant, Michelle Kendall, Lele Zhao, Anel Nurtay, David G Bonsall, and Christophe Fraser. Quantifying dynamics of sars-cov-2 transmission suggests that epidemic control and avoidance is feasible through instantaneous digital contact tracing. *medRxiv*, 2020.

[8] Leon Danon, House Thomas, and Keeling Matt J. Four key challenges in infectious disease modelling using data from multiple sources. *Epidemics*, 1:250–258, 2010.

[9] Sam Abbott, Joel Hellewell, Katharine Sherratt, Katelyn Gostic, Joe Hickson, Hamada S Badr, Michael DeWitt, Robin Thompson, EpiForecasts, and Sebastian Funk. *EpiNow2: Estimate Real-Time Case Counts and Time-Varying Epidemiological Parameters*, 2020.

[10] Stephen A. Lauer, Kyra H. Grantz, Qifang Bi, Forrest K. Jones, Qulu Zheng, Hannah R. Meredith, Andrew S. Azman, Nicholas G. Reich, and Justin Lessler. The Incubation Period of Coronavirus Disease 2019 (COVID-19) From Publicly Reported Confirmed Cases: Estimation and Application. *Annals of Internal Medicine*, 172(9):577–582, May 2020.

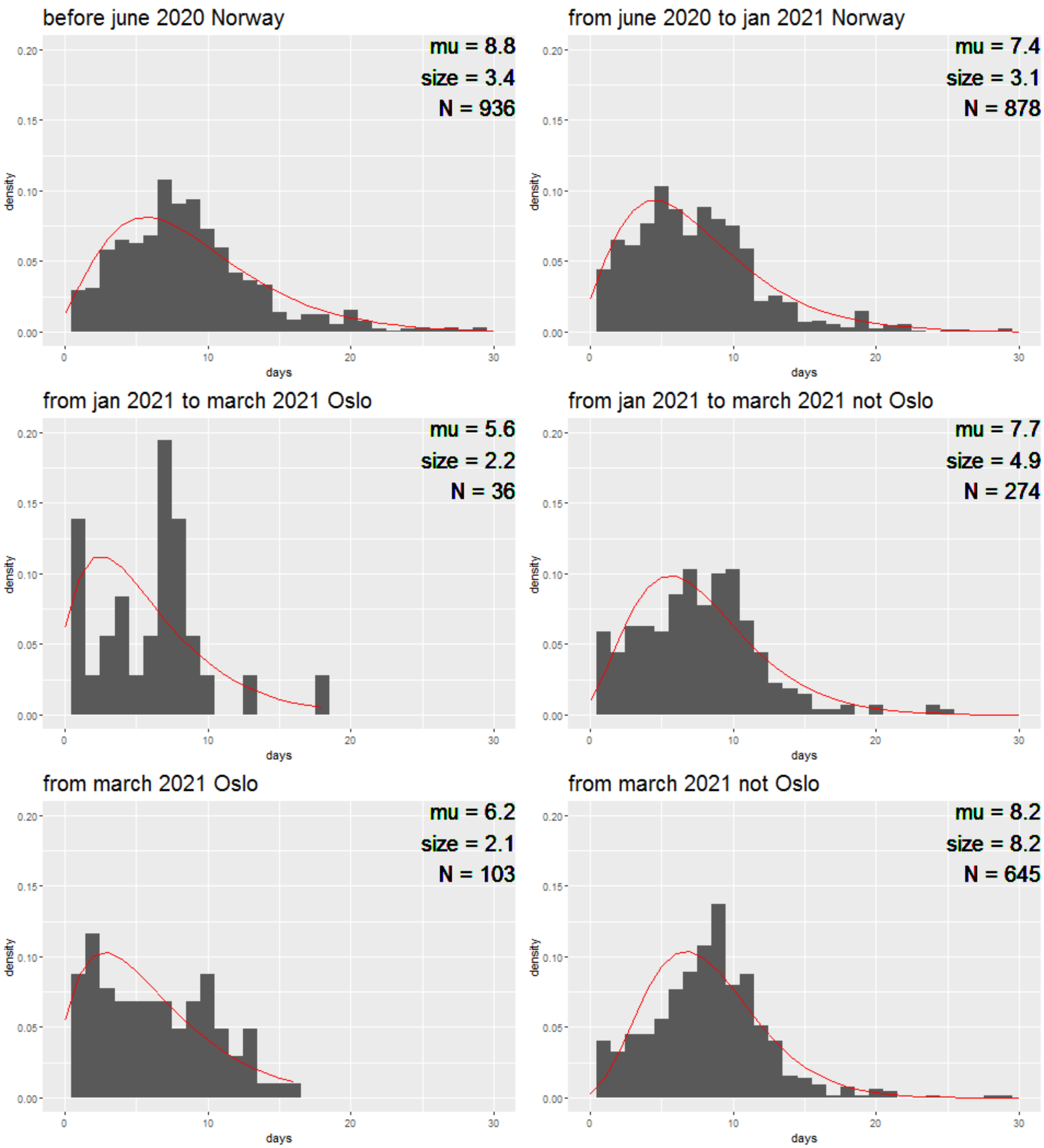

Fig A: Histogram of time from symptom onset to hospitalisation for different time-space periods. Red line shows the fitted negative binomial distribution described with parameters (mu and size) in the upper right corner. N is the number of observations in each histogram.

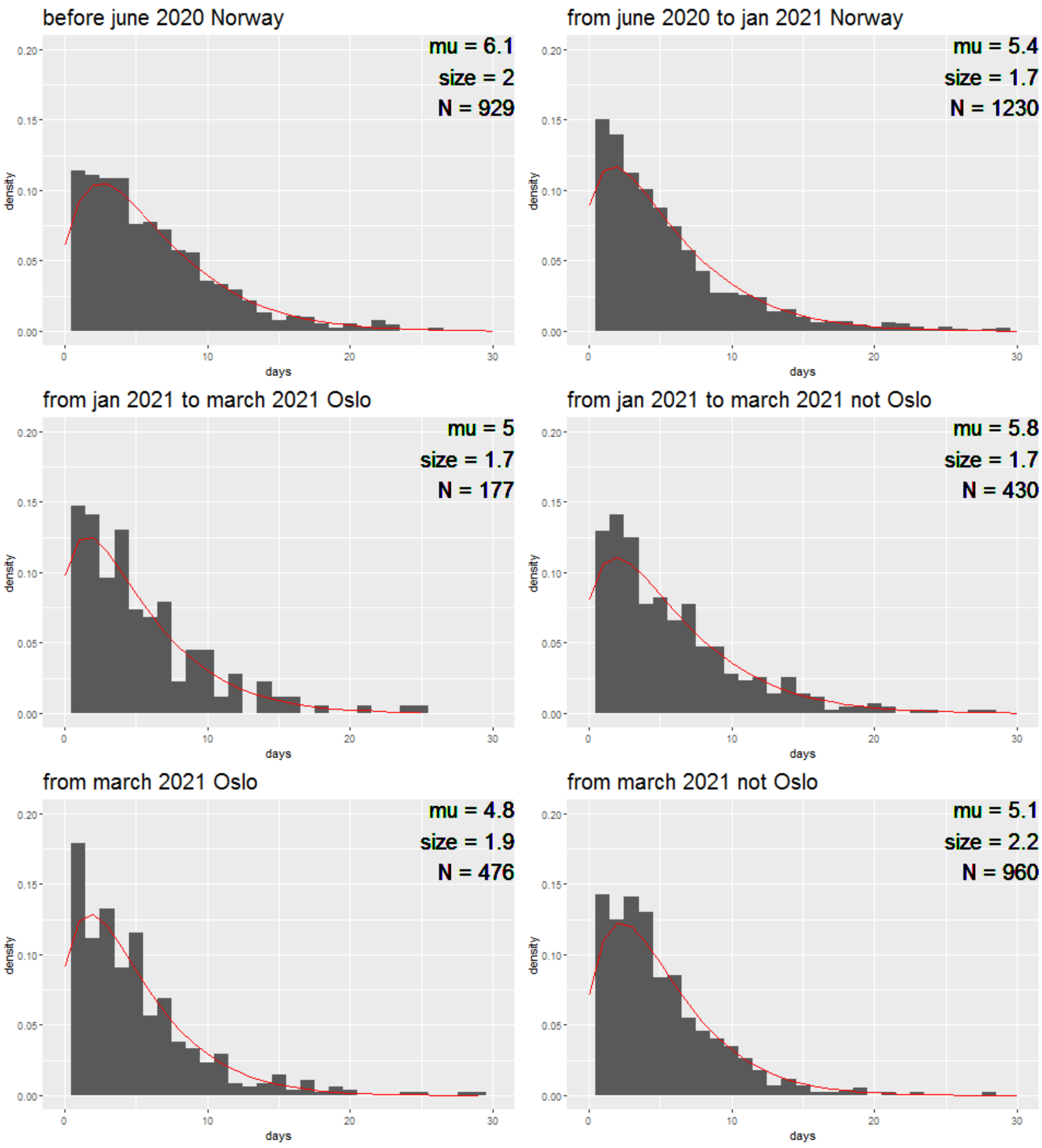

Fig B: Time from hospitalisation to discharge without respirator treatment for different time and space periods. Red line shows the fitted negative binomial distribution described with parameters (mu and size) in the upper right corner. N is the number of observations in each histogram.

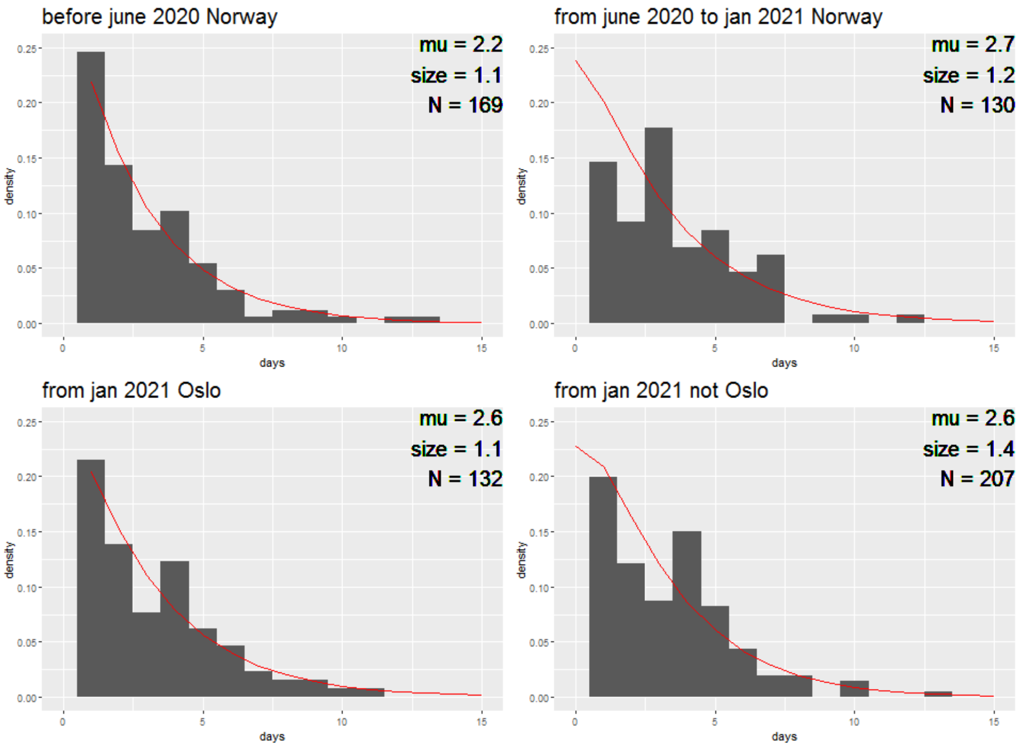

Fig C: Time from hospitalisation to ventilator treatment for different time and space periods. Red line shows the fitted negative binomial distribution described with parameters (mu and size) in the upper right corner. N is the number of observations in each histogram.

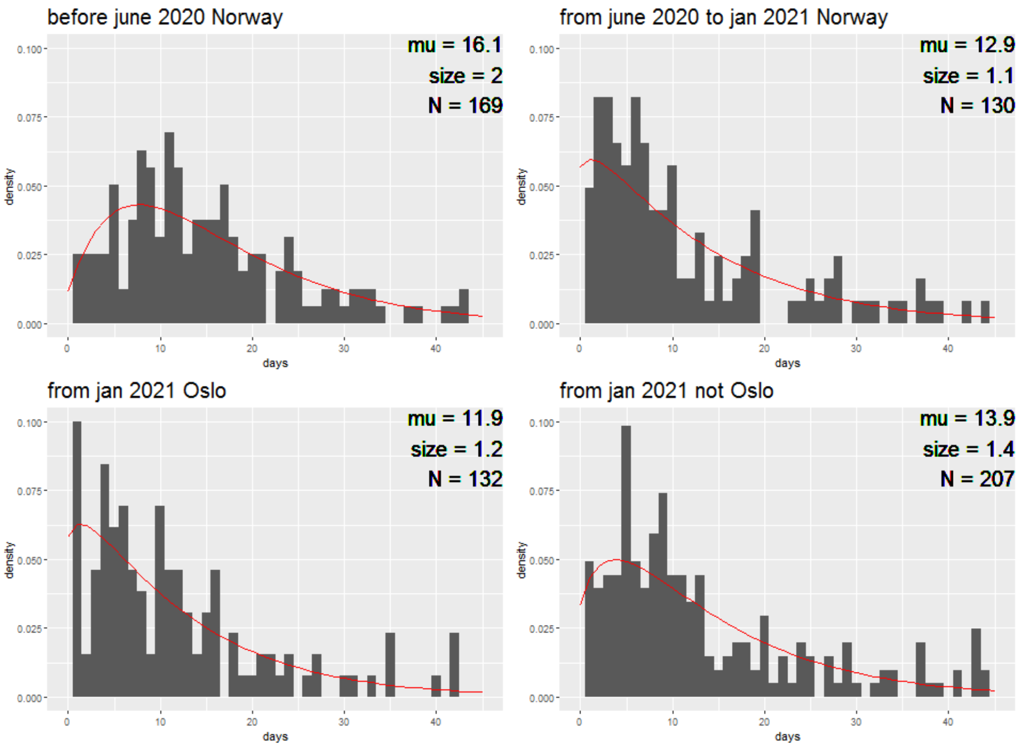

Fig D: Time on ventilator treatment for different time and space periods. Red line shows the fitted negative binomial distribution described with parameters (mu and size) in the upper right corner. N is the number of observations in each histogram.

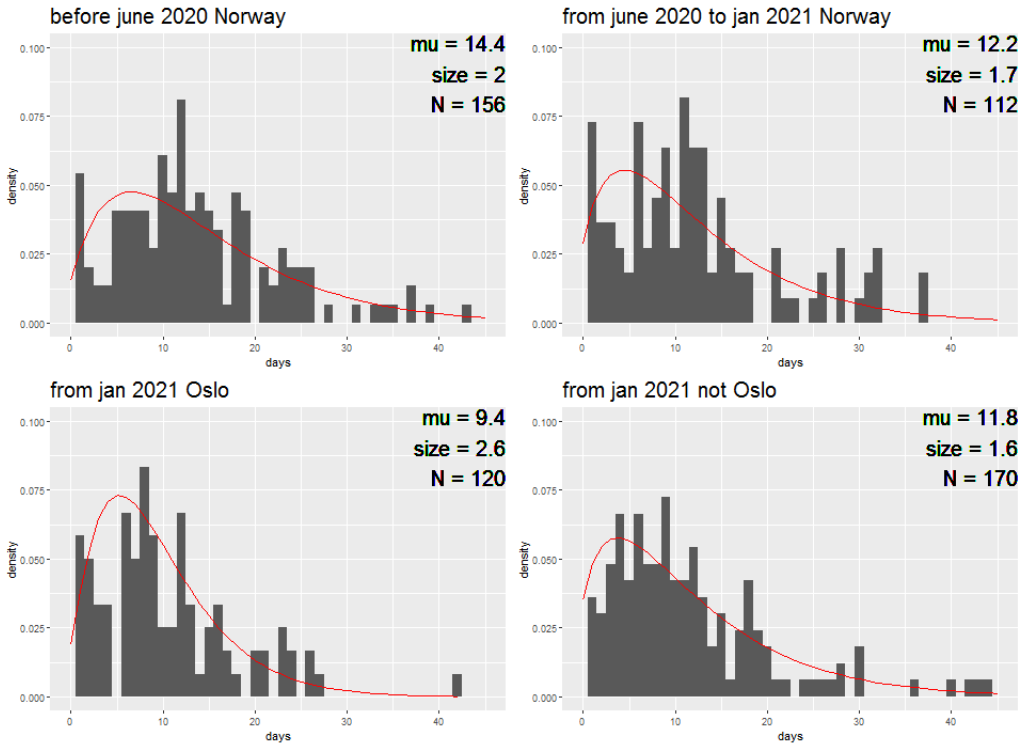

Fig E: Time after ventilator treatment for different time and space periods. Red line shows the fitted negative binomial distribution described with parameters (mu and size) in the upper right corner. N is the number of observations in each histogram.

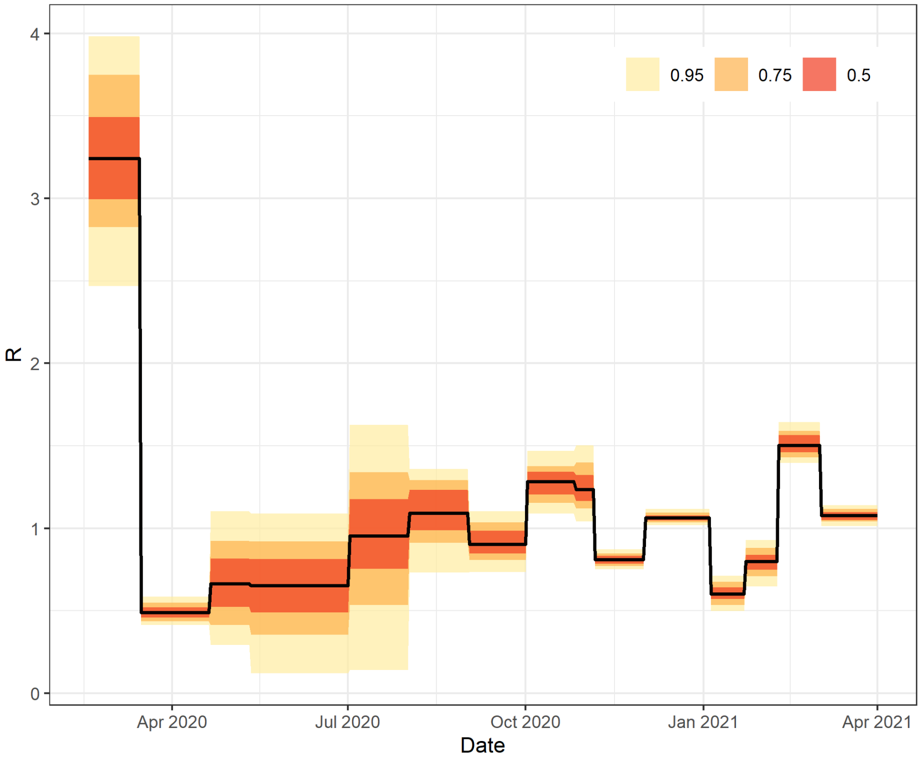

Fig F: Estimated national reproductive numbers (black line, median) with 75% and 95% credible intervals.

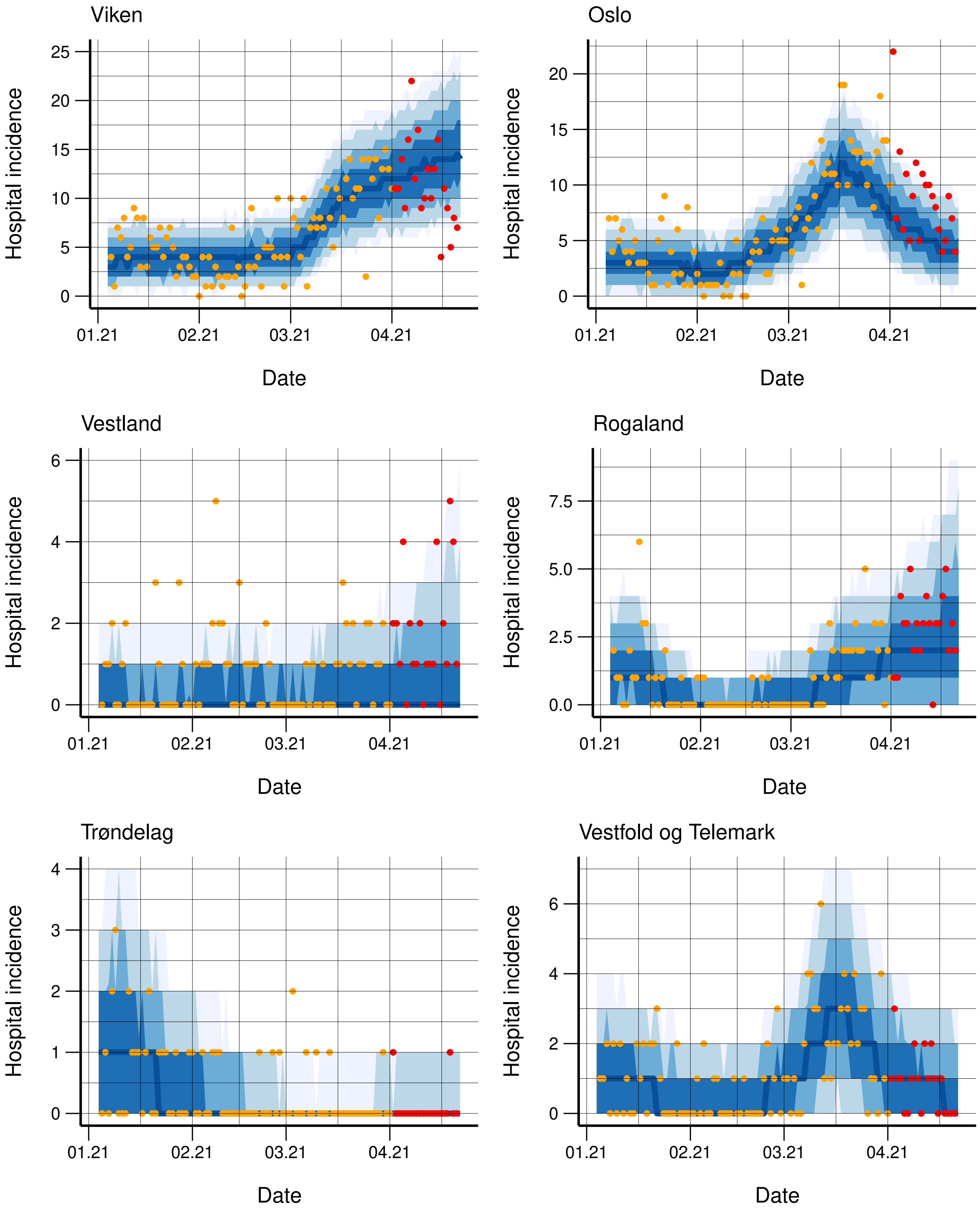

Fig G: Observed (orange dots) and simulated hospitalisation incidence for each region, along with 95%, 90%, 75%, and 50% credibility intervals. The regions are ordered according to population, with the capital Oslo as second. The incidence for the next three weeks not used in the calibration is provided in red.

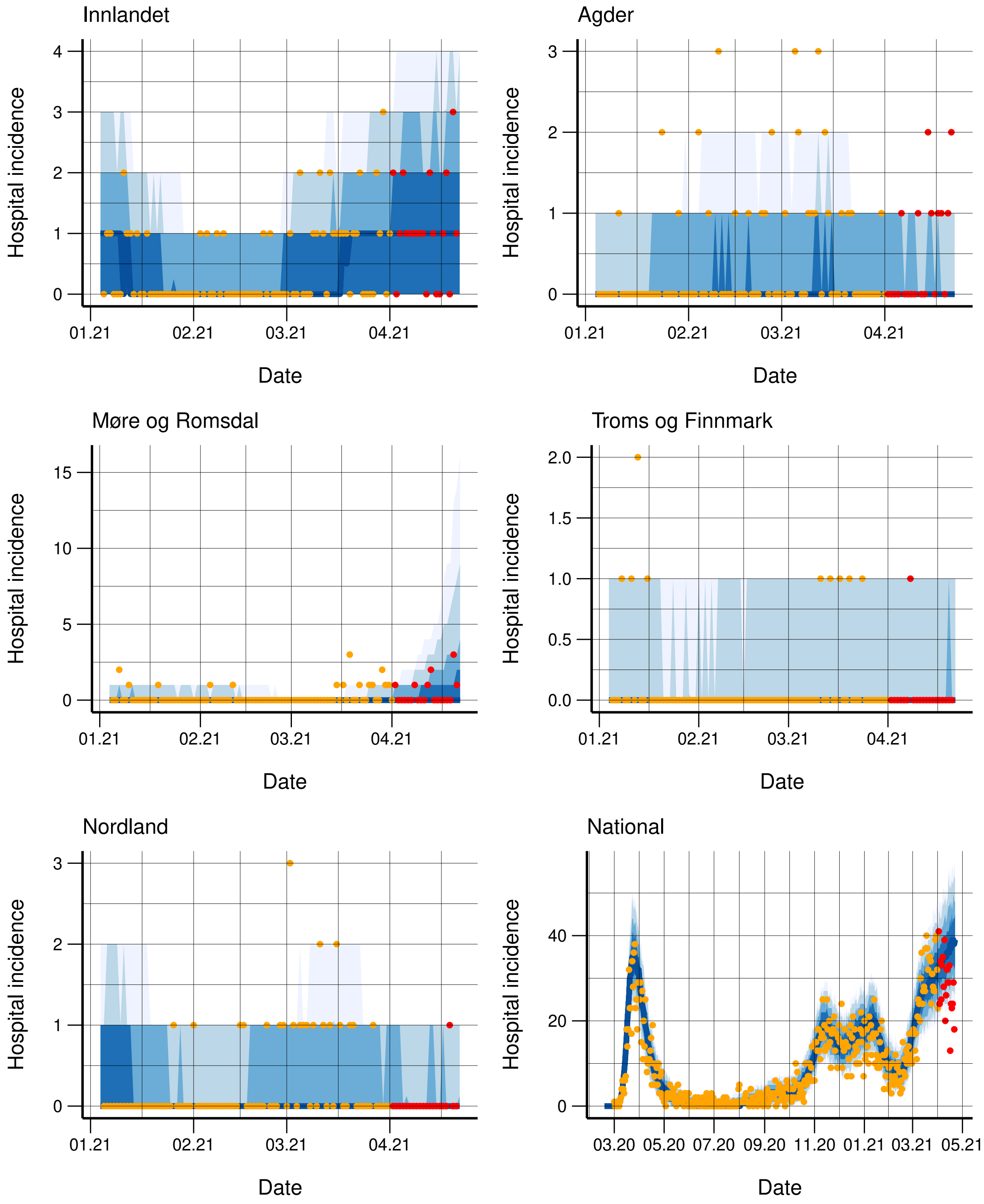

Fig H: Observed (orange dots) and simulated hospitalisation incidence for each region, along with 95%, 90%, 75%, and 50% credibility intervals. The regions are ordered according to population. In the lower right panel, we have the national fit for the model assuming the same transmissibility in all counties. The incidence for the next three weeks not used in the calibration is provided in red.

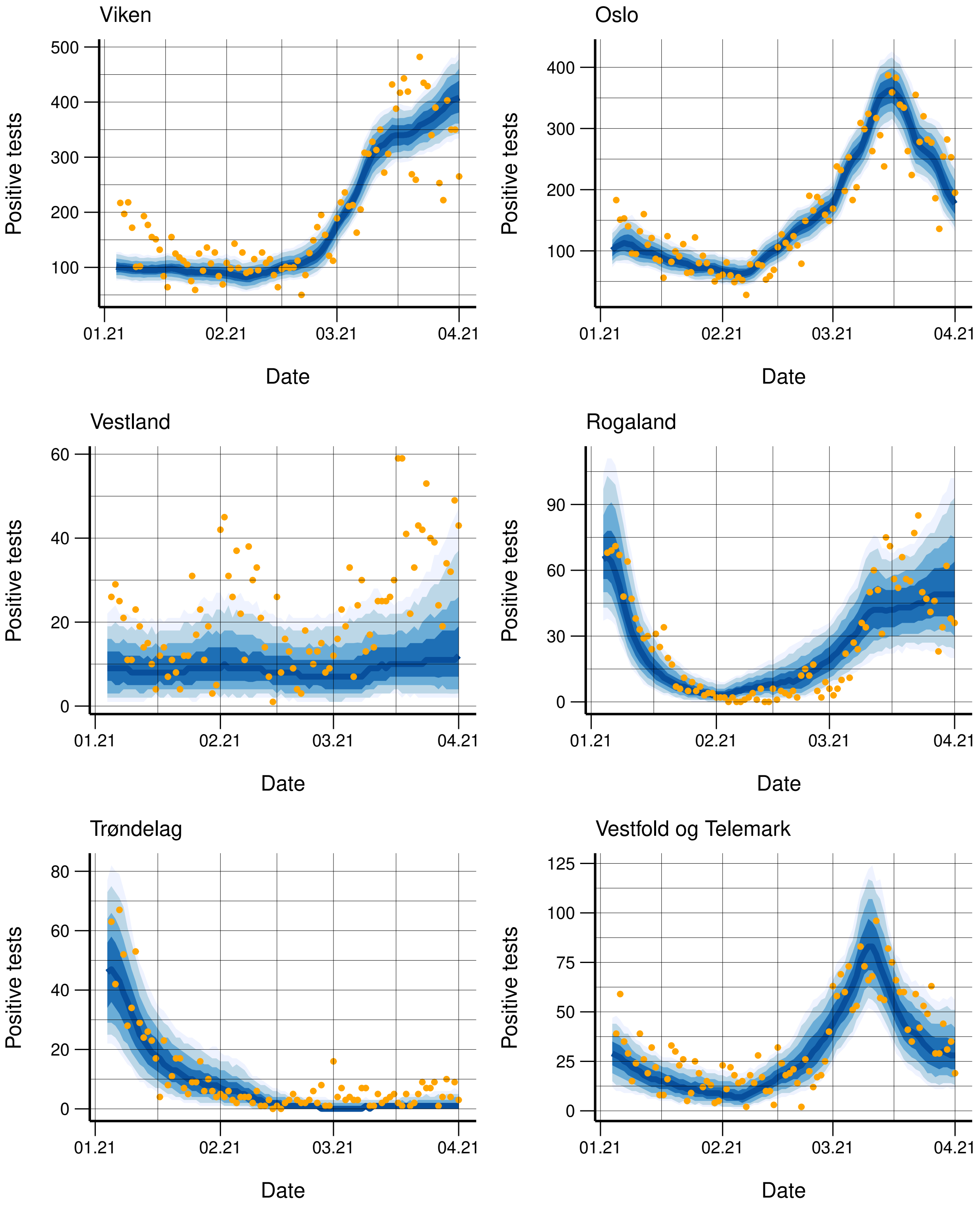

Fig I: Observed (orange dots) and simulated positive tests for each region, along with 95%, 90%, 75%, and 50% credibility intervals. The regions are ordered according to population, with the capital Oslo as second.

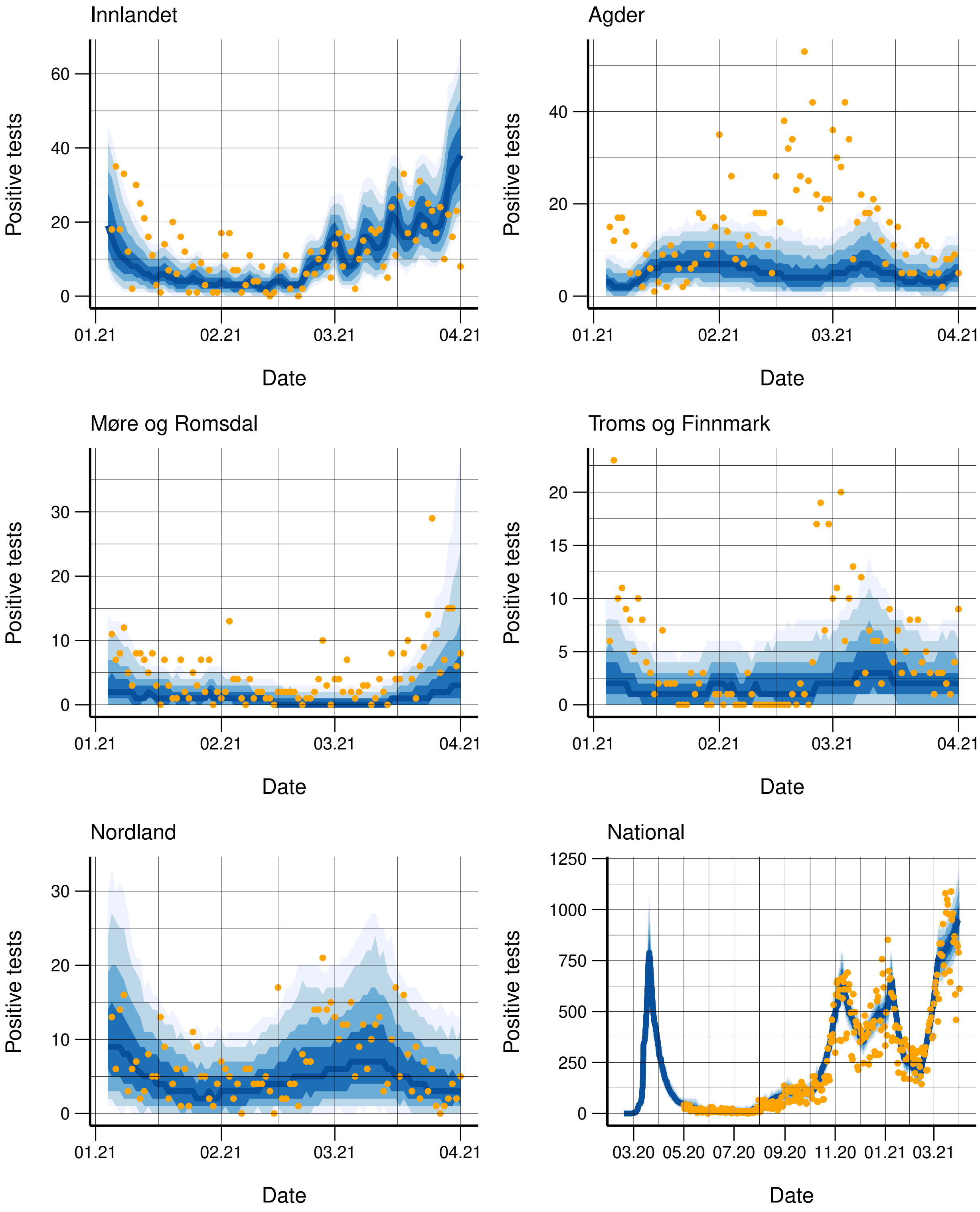

Fig J: Observed (orange dots) and simulated positive tests for each region, along with 95%, 90%, 75%, and 50% credibility intervals. The regions are ordered according to population. In the lower right panel, we have the national fit for the model assuming the same transmissibility in all counties.

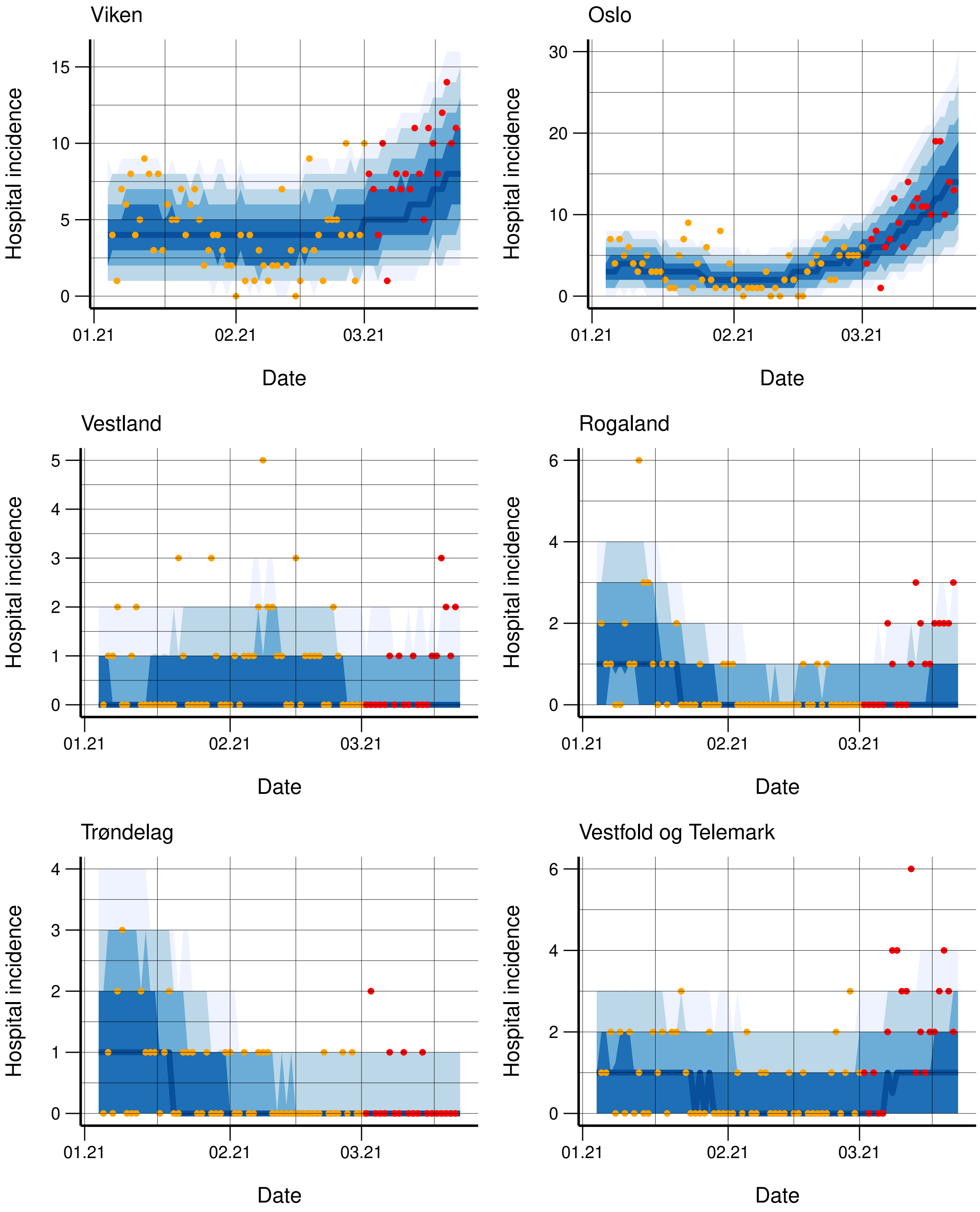

Fig K: Observed (orange dots) and simulated hospitalisation incidence for each region, along with 95%, 90%, 75%, and 50% credibility intervals. The regions are ordered according to population, with the capital Oslo as second. The incidence for the next three weeks not used in the calibration is provided in red.

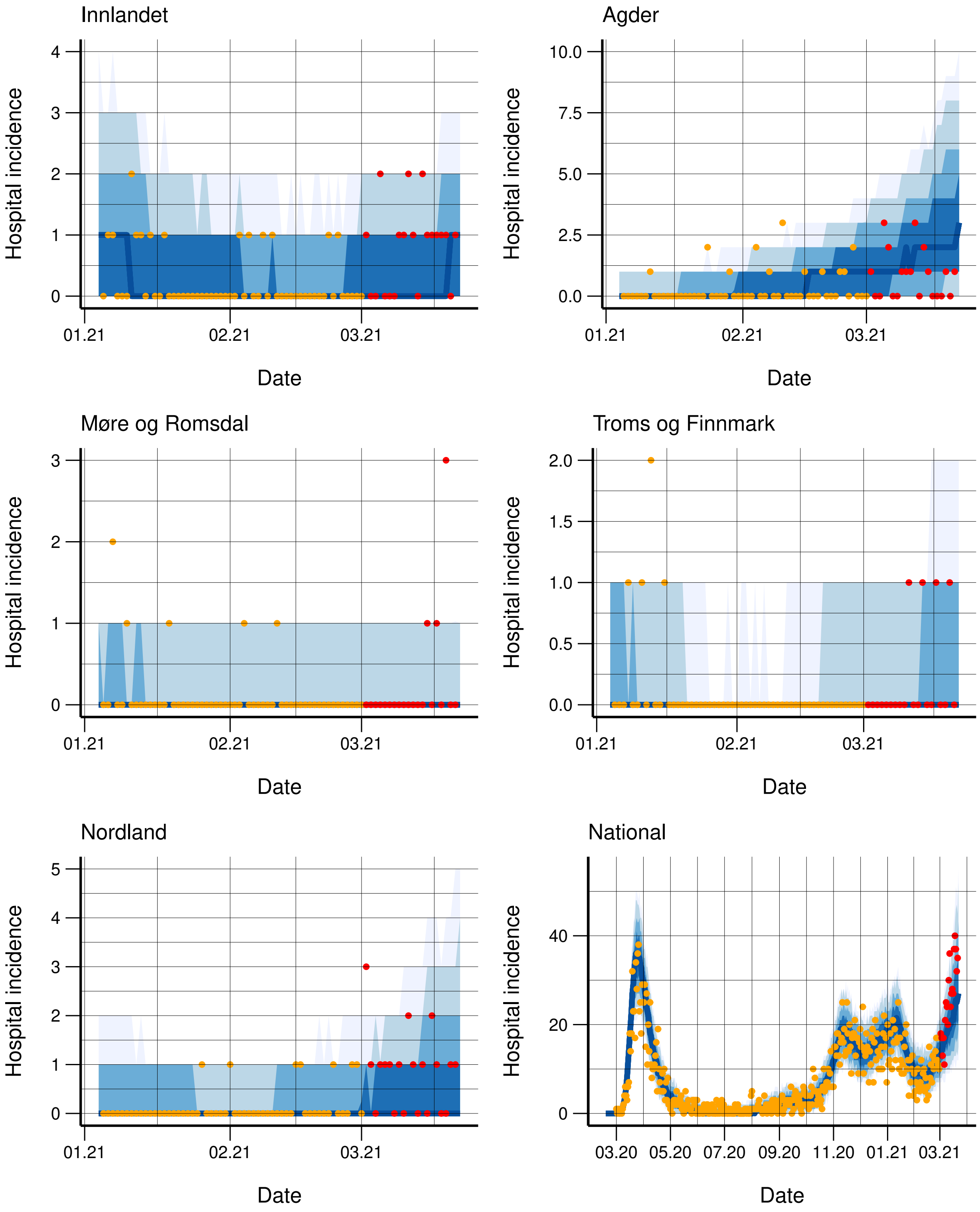

Fig L: Observed (orange dots) and simulated hospitalisation incidence for each region, along with 95%, 90%, 75%, and 50% credibility intervals. The regions are ordered according to population. In the lower right panel, we have the national fit for the model assuming the same transmissibility in all counties. The incidence for the next three weeks not used in the calibration is provided in red.

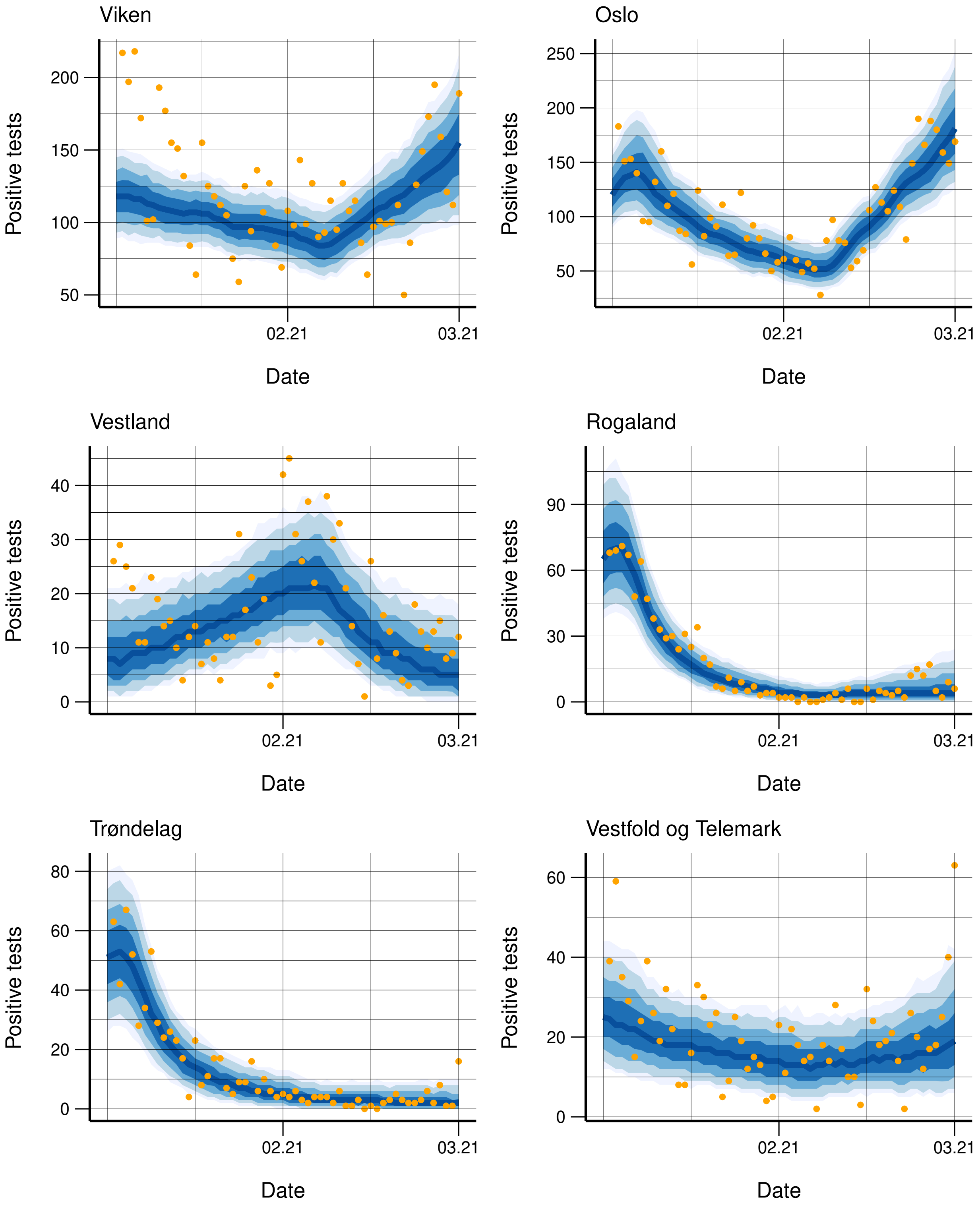

Fig M: Observed (orange dots) and simulated positive tests for each region, along with 95%, 90%, 75%, and 50% credibility intervals. The regions are ordered according to population, with the capital Oslo as second.

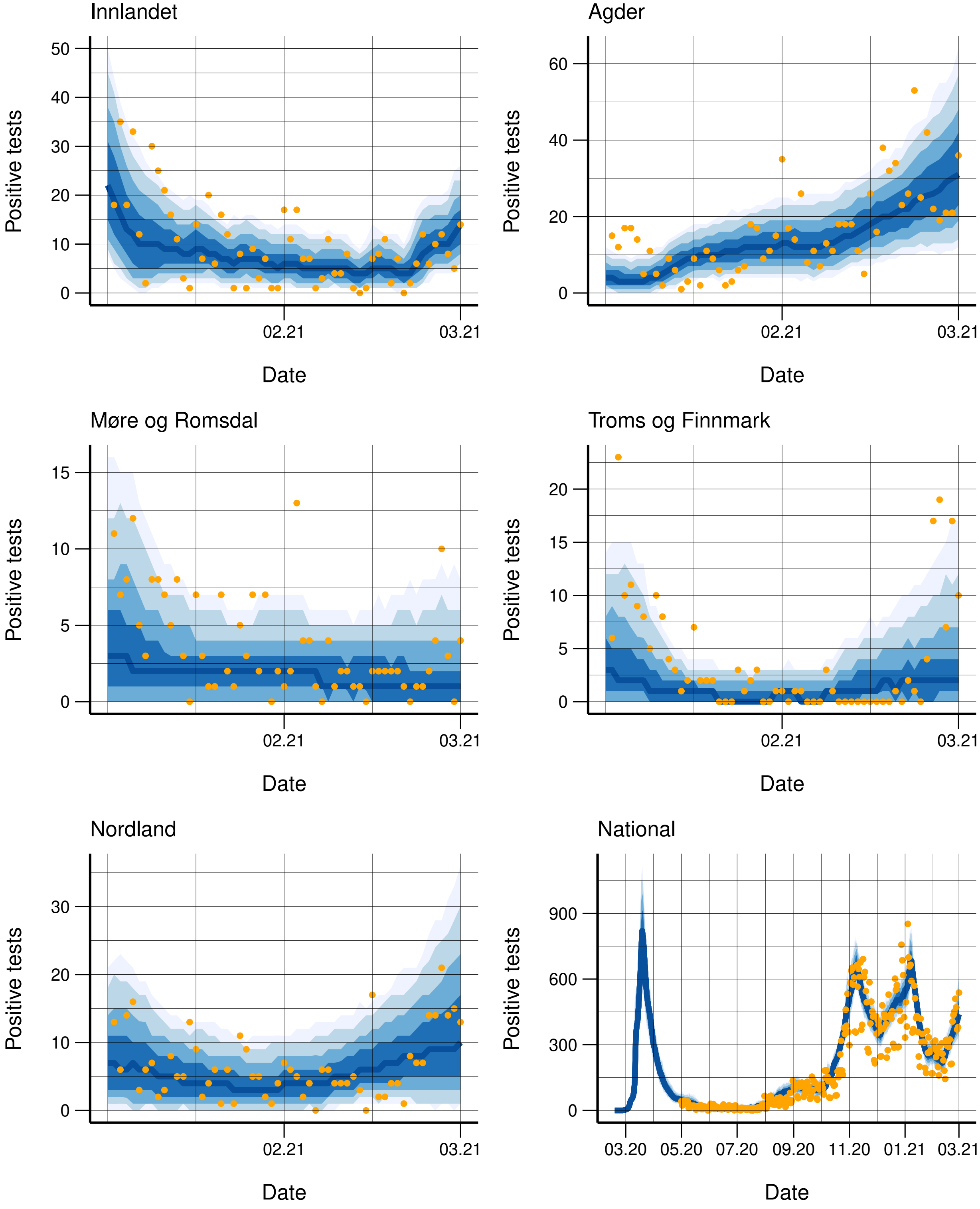

Fig N: Observed (orange dots) and simulated positive tests for each region, along with 95%, 90%, 75%, and 50% credibility intervals. The regions are ordered according to population. In the lower right panel, we have the national fit for the model assuming the same transmissibility in all counties.

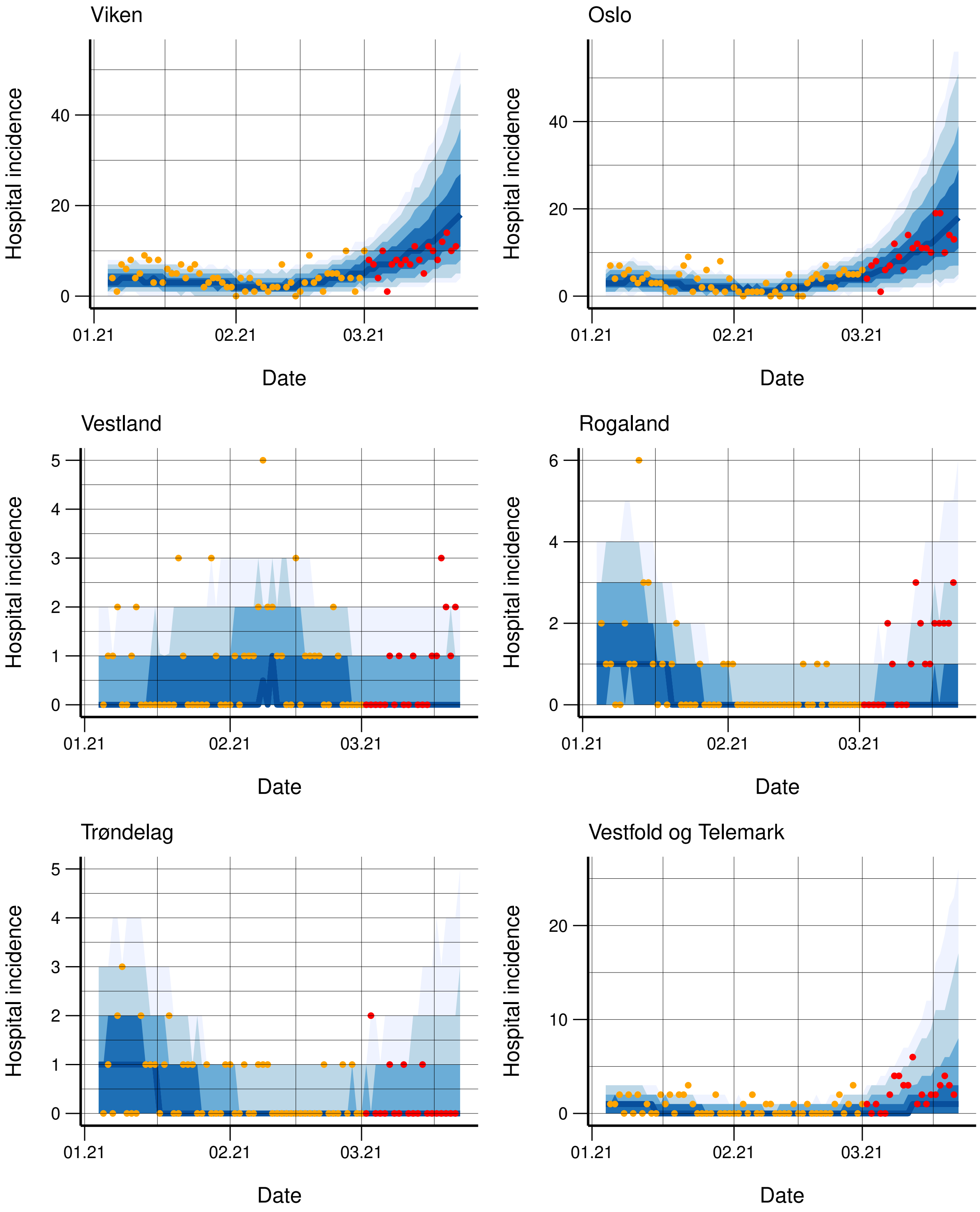

Fig O: Observed (orange dots) and simulated hospitalisation incidence for each region, along with 95%, 90%, 75%, and 50% credibility intervals. The regions are ordered according to population, with the capital Oslo as second. The incidence for the next three weeks not used in the calibration is provided in red.

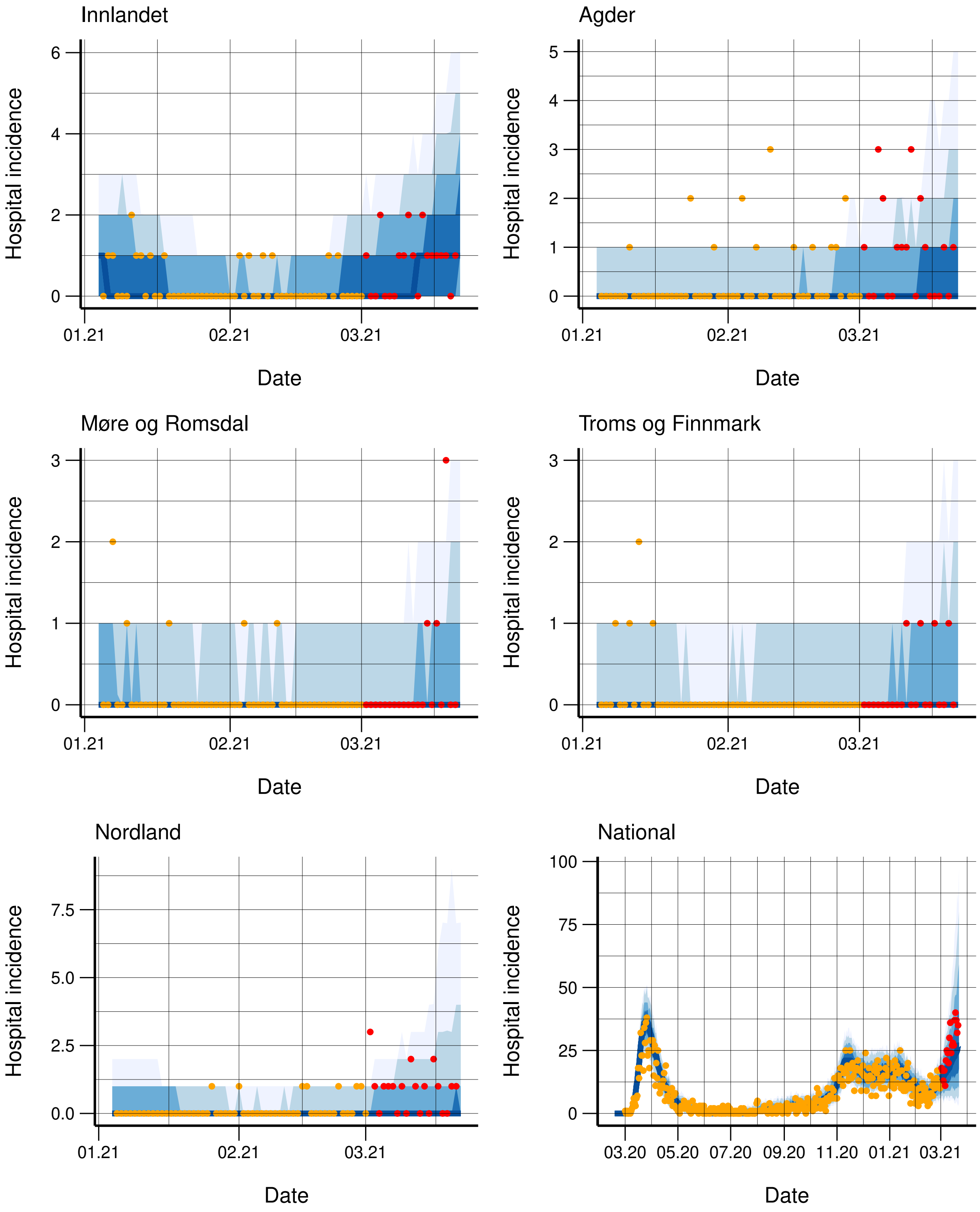

Fig P: Observed (orange dots) and simulated hospitalisation incidence for each region, along with 95%, 90%, 75%, and 50% credibility intervals. The regions are ordered according to population. In the lower right panel, we have the national fit for the model assuming the same transmissibility in all counties. The incidence for the next three weeks not used in the calibration is provided in red.

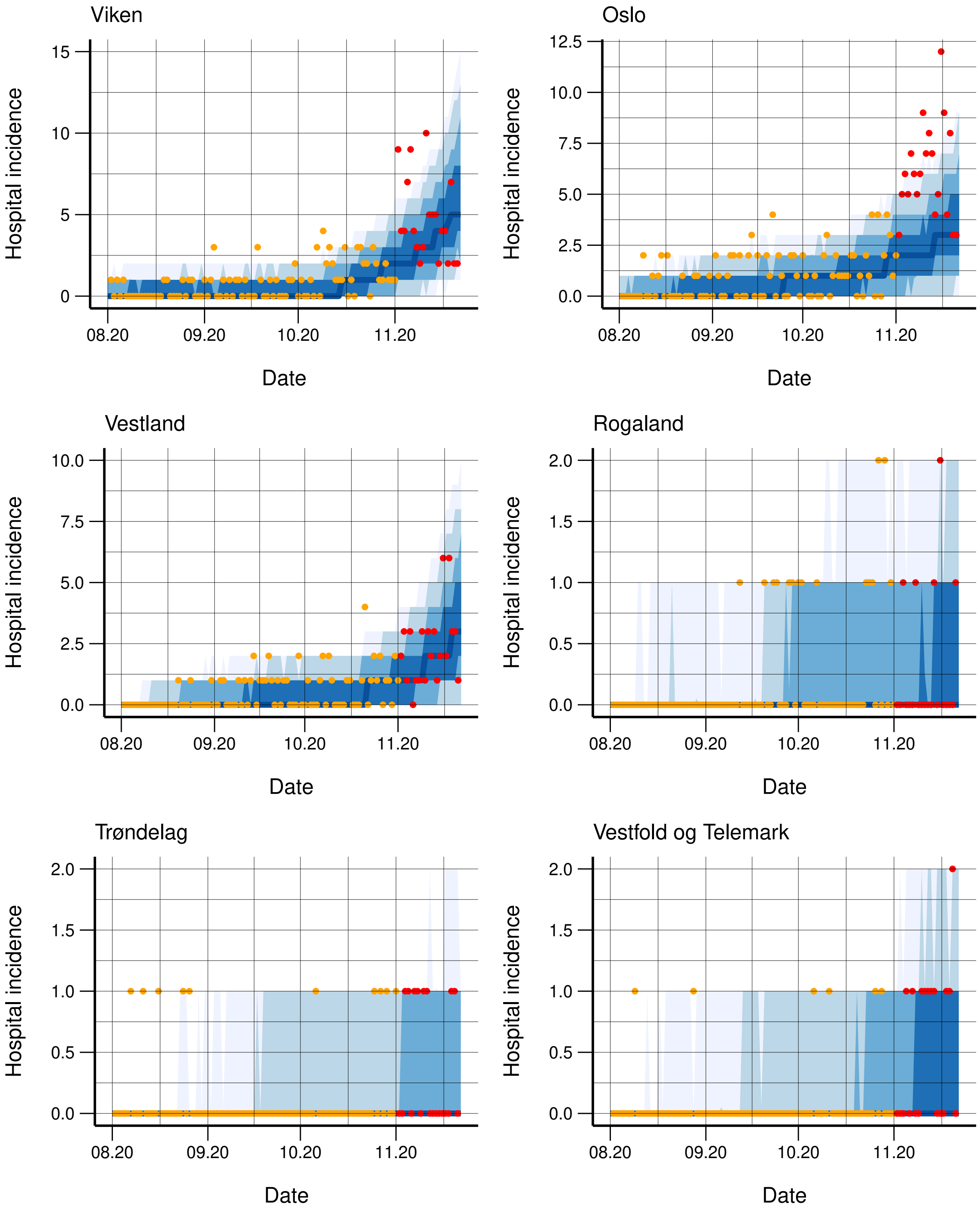

Fig Q: Observed (orange dots) and simulated hospitalisation incidence for each region, along with 95%, 90%, 75%, and 50% credibility intervals. The regions are ordered according to population, with the capital Oslo as second. The incidence for the next three weeks not used in the calibration is provided in red.

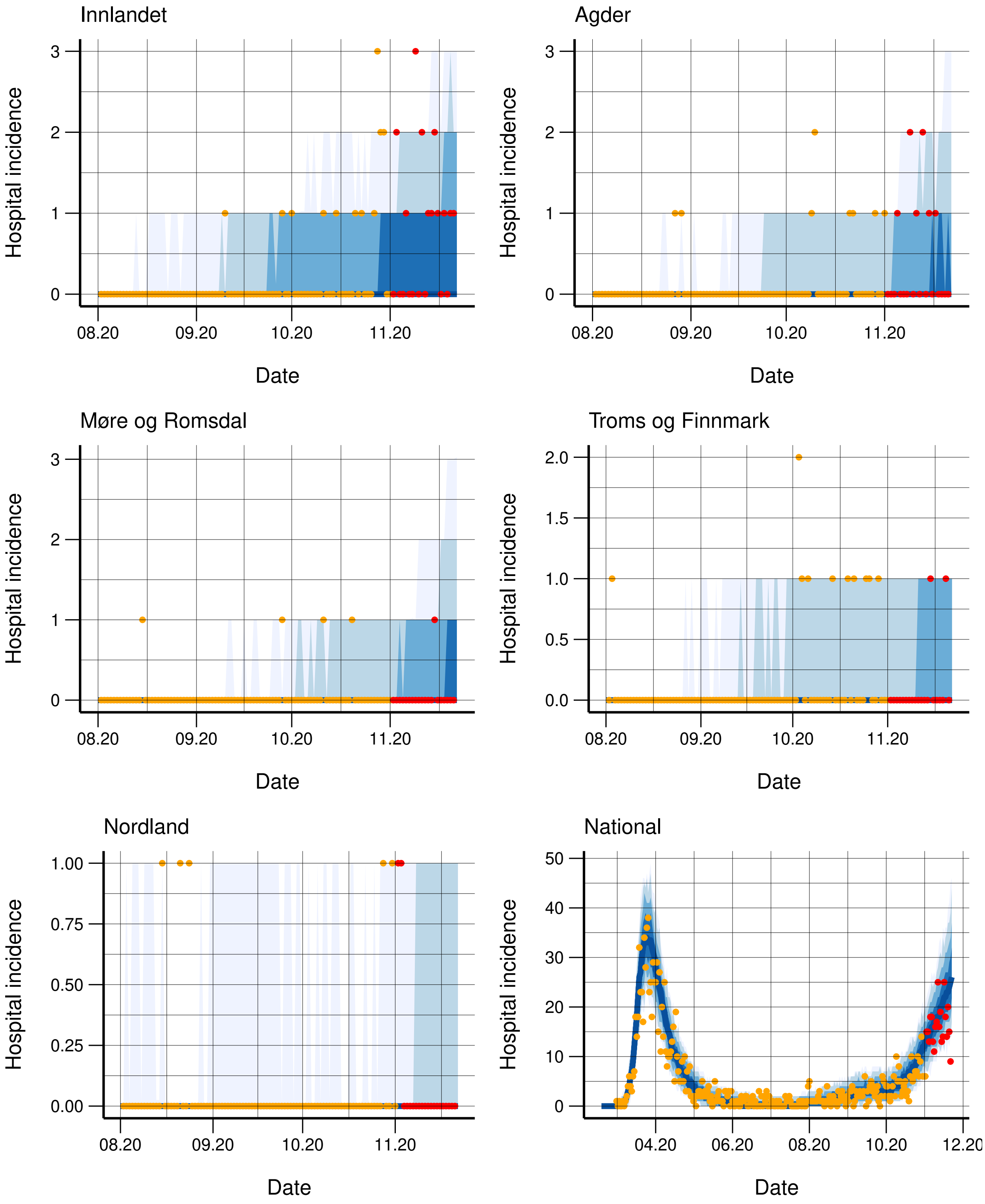

Fig R: Observed (orange dots) and simulated hospitalisation incidence for each region, along with 95%, 90%, 75%, and 50% credibility intervals. The regions are ordered according to population. In the lower right panel, we have the national fit for the model assuming the same transmissibility in all counties. The incidence for the next three weeks not used in the calibration is provided in red.

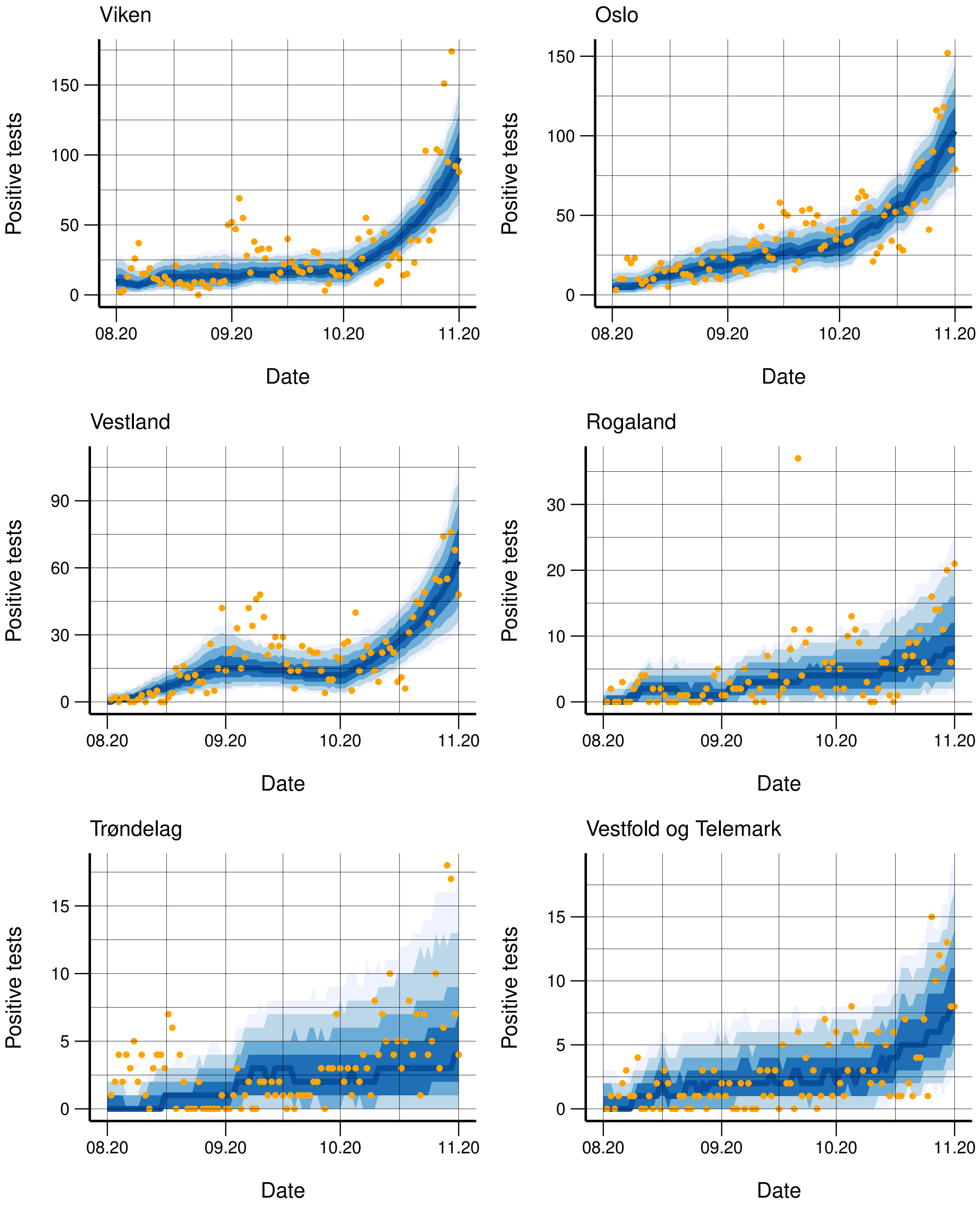

Fig S: Observed (orange dots) and simulated positive tests for each region, along with 95%, 90%, 75%, and 50% credibility intervals. The regions are ordered according to population, with the capital Oslo as second.

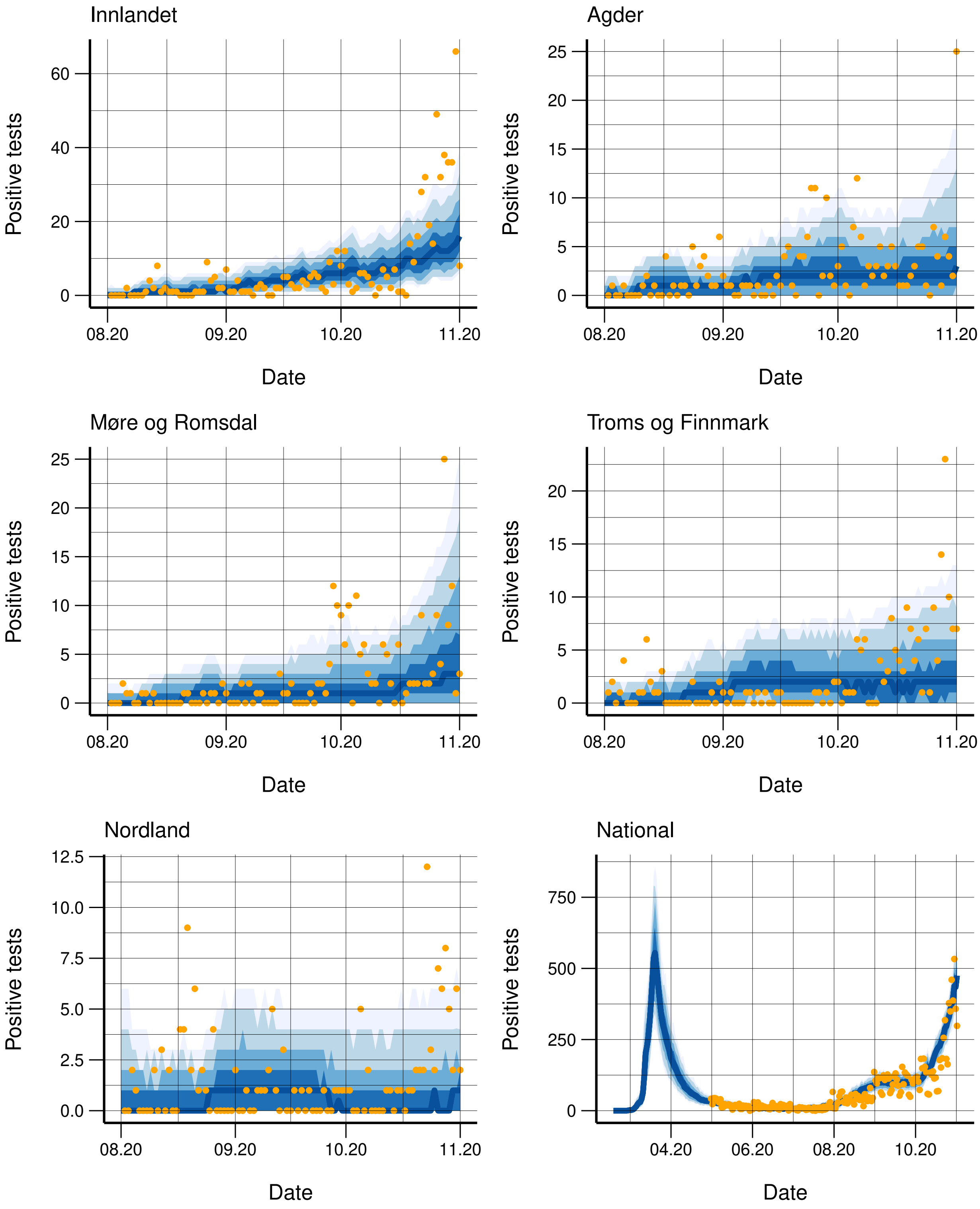

Fig T: Observed (orange dots) and simulated positive tests for each region, along with 95%, 90%, 75%, and 50% credibility intervals. The regions are ordered according to population. In the lower right panel, we have the national fit for the model assuming the same transmissibility in all counties.

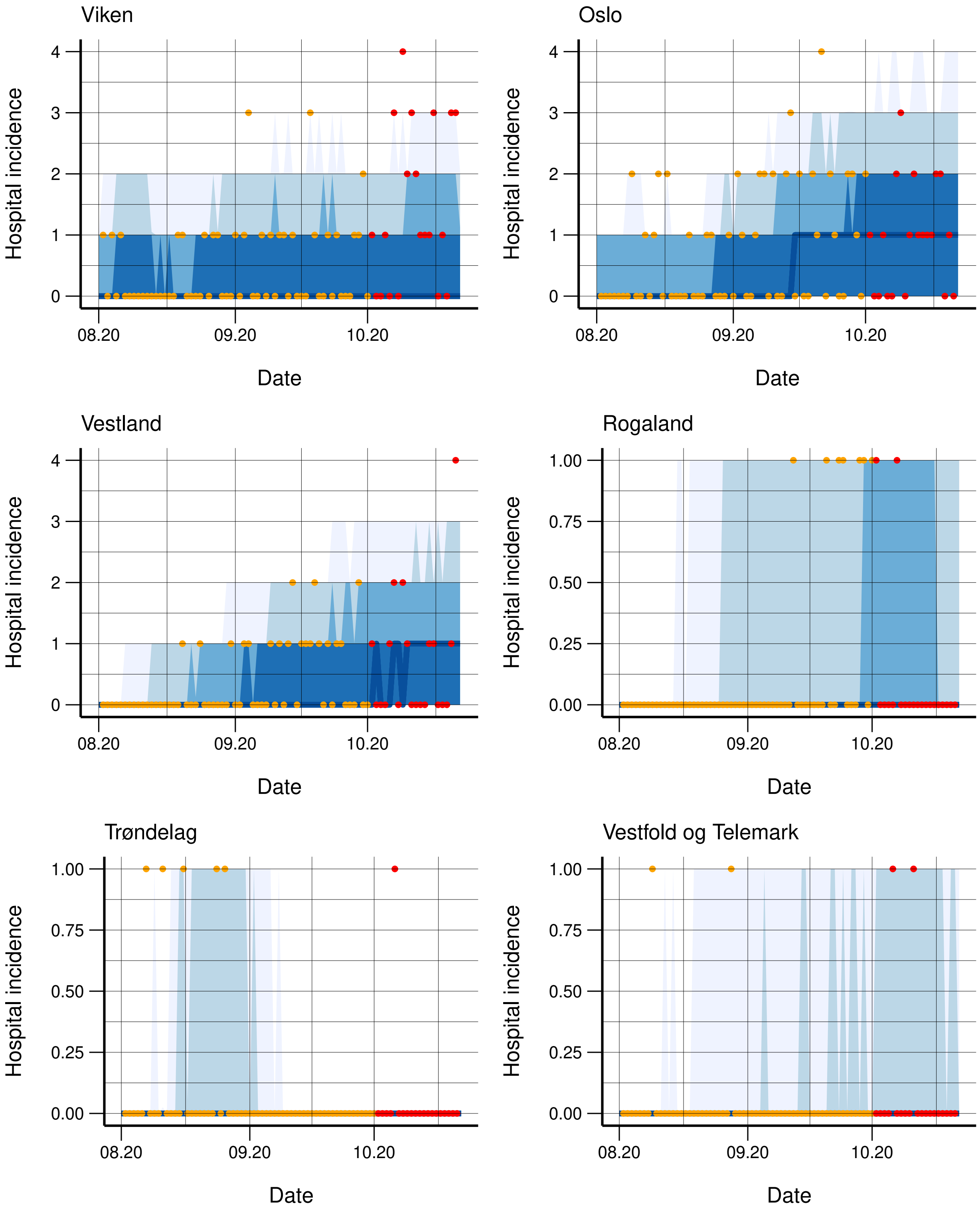

Fig U: Observed (orange dots) and simulated hospitalisation incidence for each region, along with 95%, 90%, 75%, and 50% credibility intervals. The regions are ordered according to population, with the capital Oslo as second. The incidence for the next three weeks not used in the calibration is provided in red.

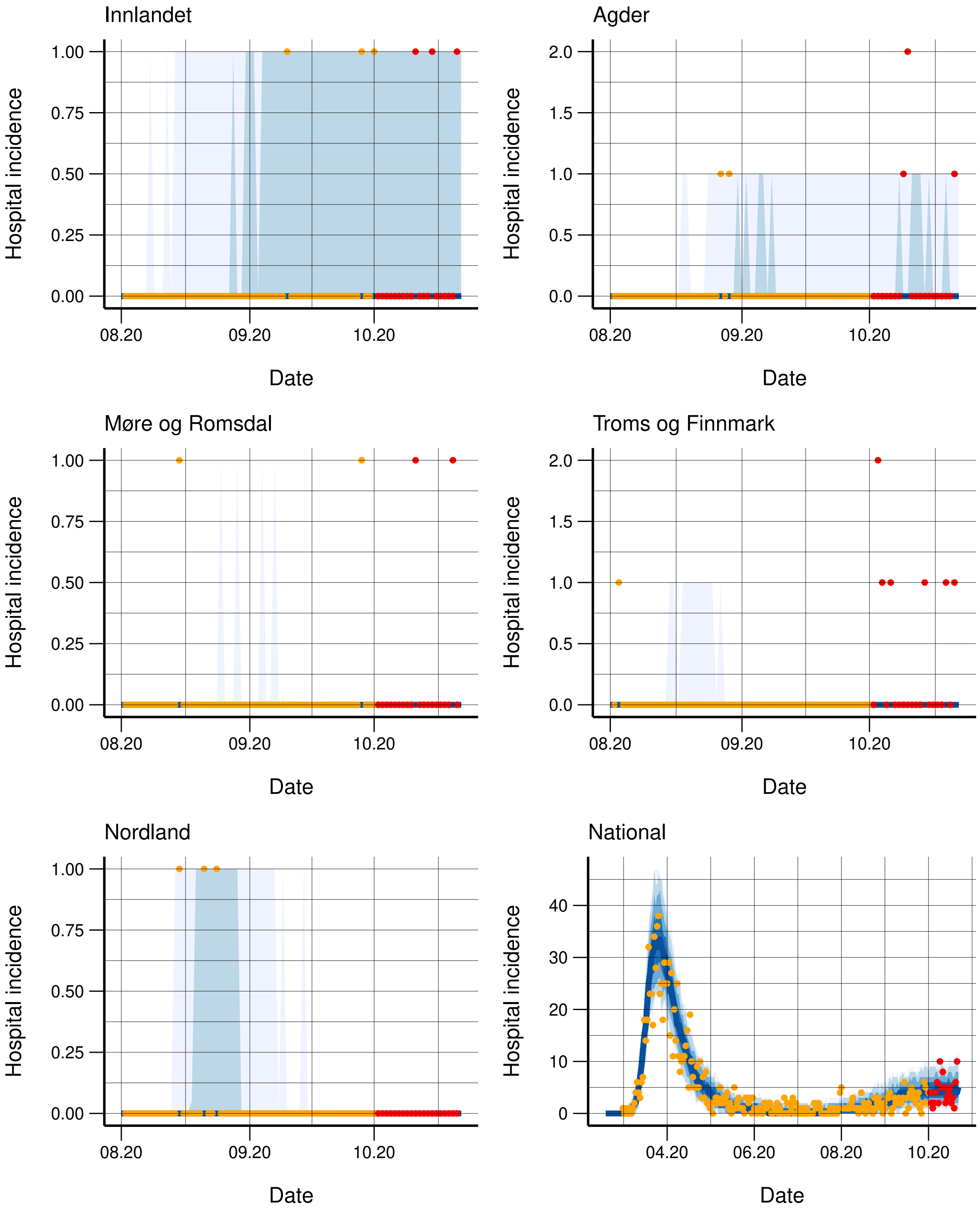

Fig V: Observed (orange dots) and simulated hospitalisation incidence for each region, along with 95%, 90%, 75%, and 50% credibility intervals. The regions are ordered according to population. In the lower right panel, we have the national fit for the model assuming the same transmissibility in all counties. The incidence for the next three weeks not used in the calibration is provided in red.

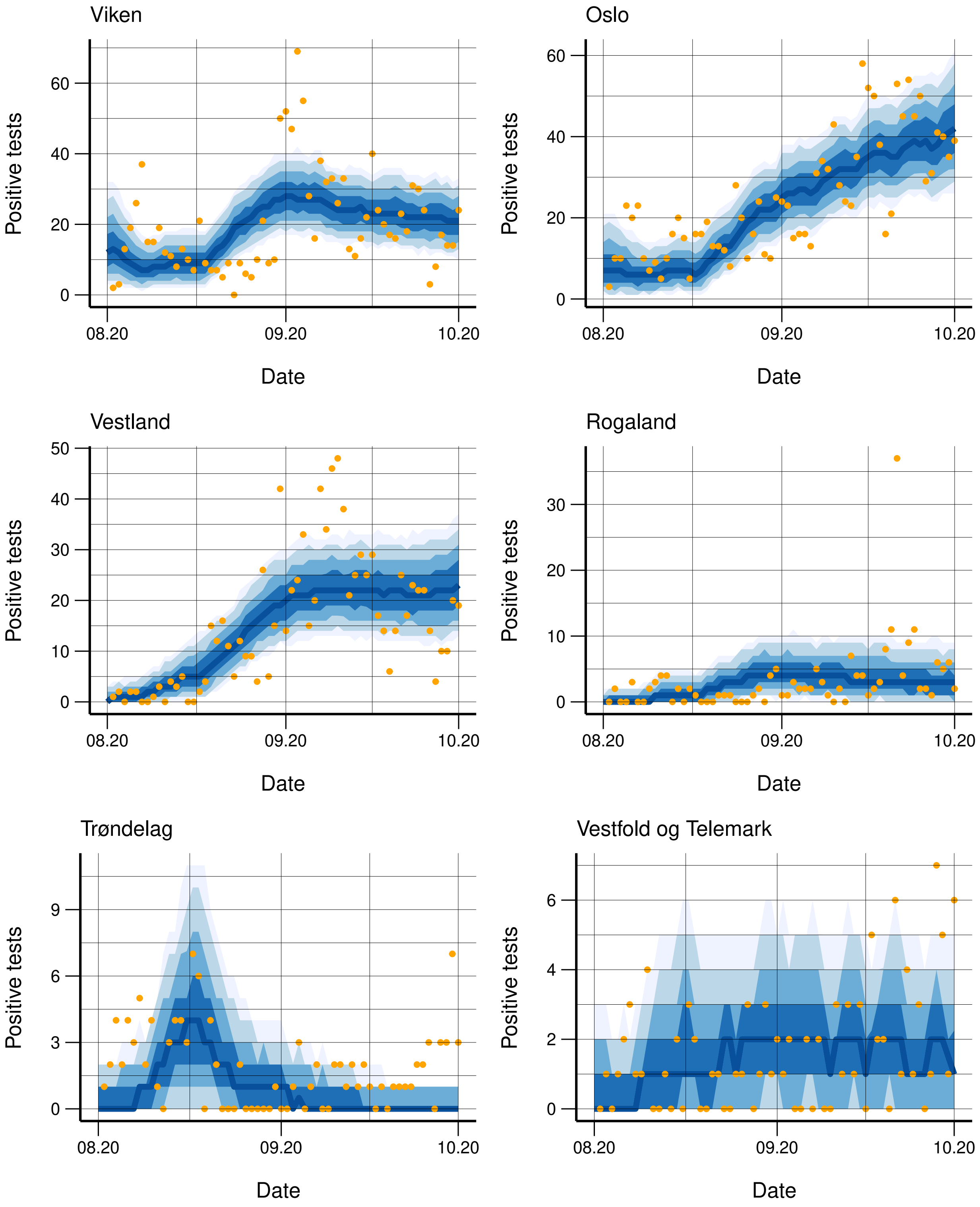

Fig W: Observed (orange dots) and simulated positive tests for each region, along with 95%, 90%, 75%, and 50% credibility intervals. The regions are ordered according to population, with the capital Oslo as second.

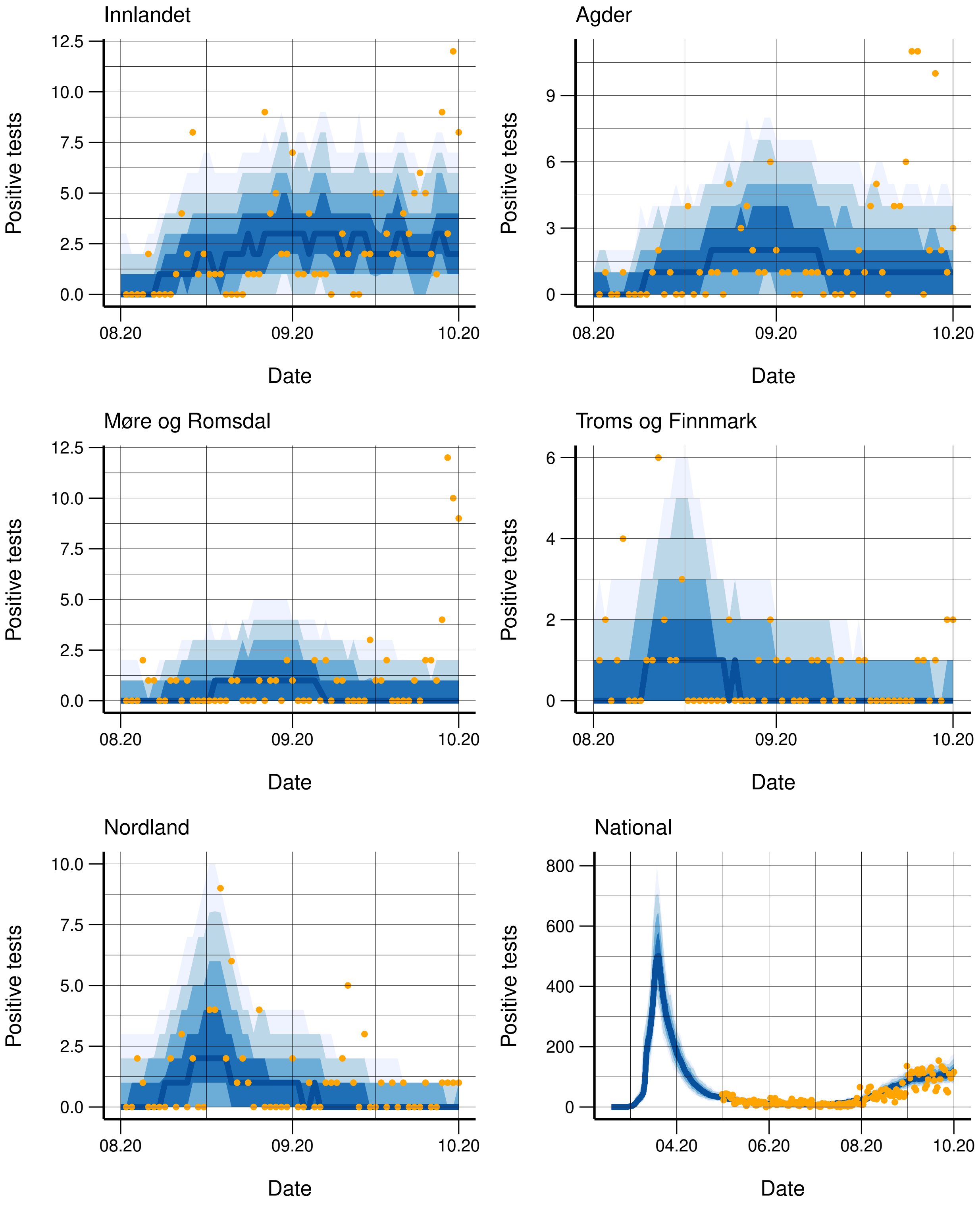

Fig X: Observed (orange dots) and simulated positive tests for each region, along with 95%, 90%, 75%, and 50% credibility intervals. The regions are ordered according to population. In the lower right panel, we have the national fit for the model assuming the same transmissibility in all counties.

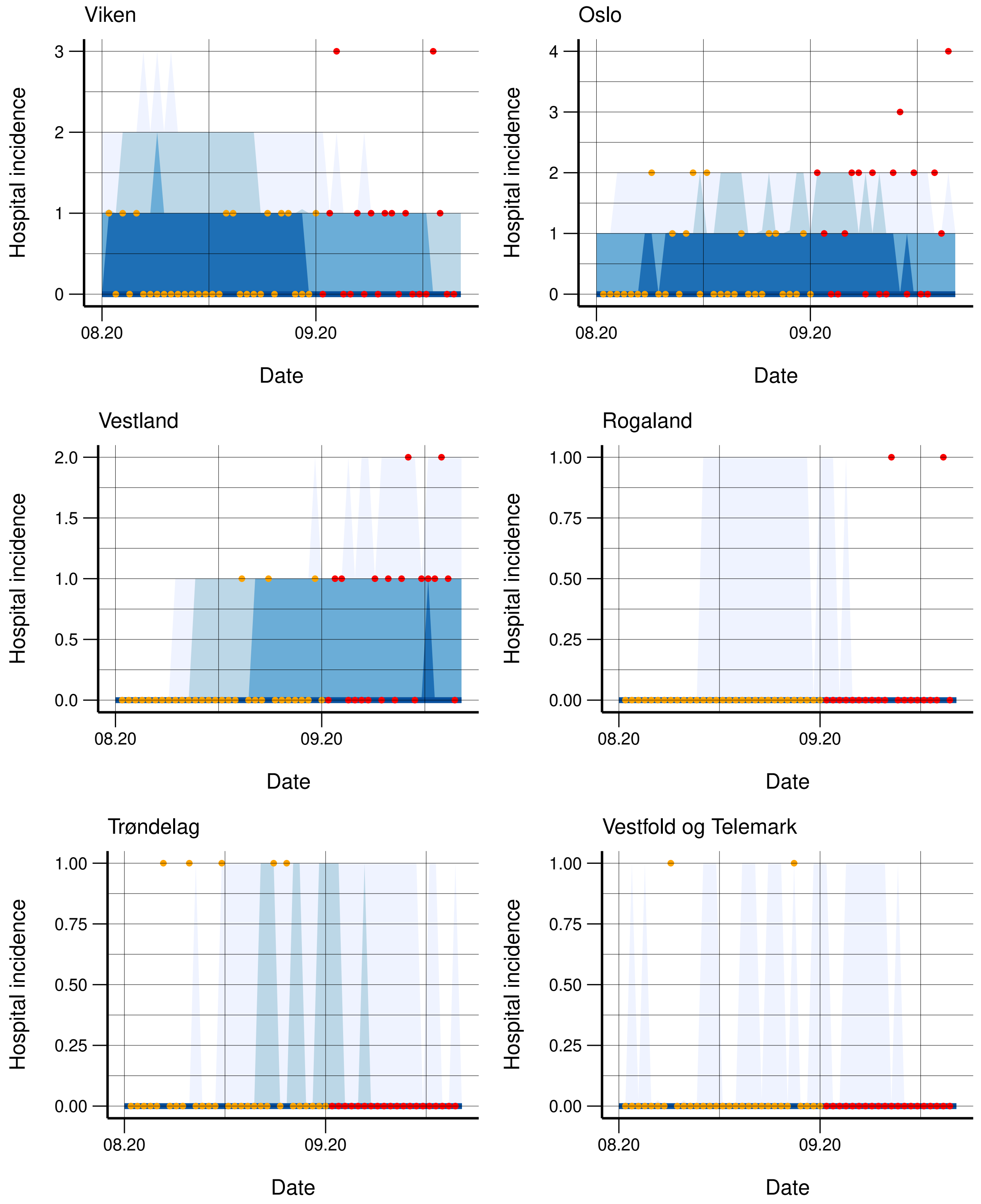

Fig Y: Observed (orange dots) and simulated hospitalisation incidence for each region, along with 95%, 90%, 75%, and 50% credibility intervals. The regions are ordered according to population, with the capital Oslo as second. The incidence for the next three weeks not used in the calibration is provided in red.

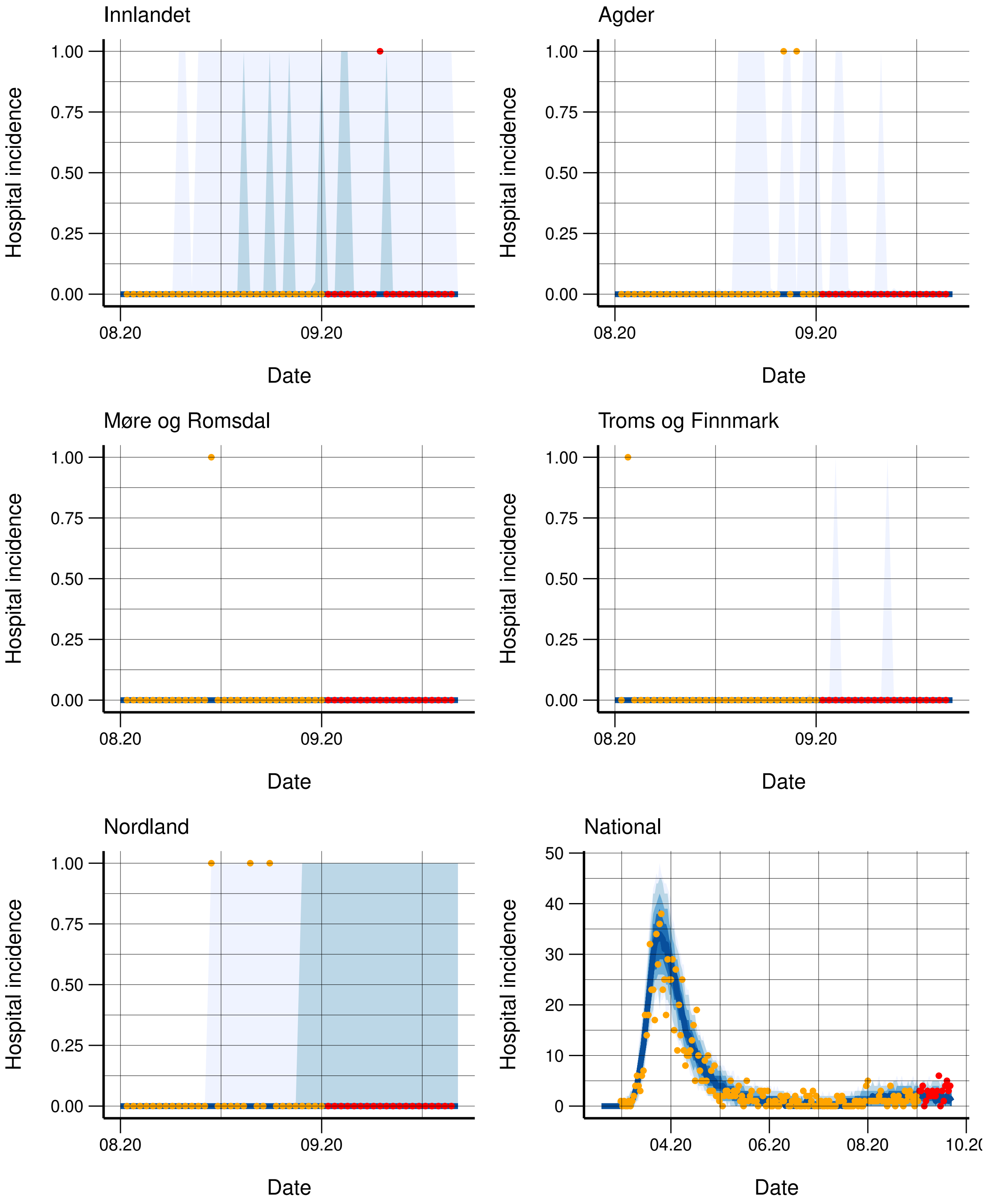

Fig Z: Observed (orange dots) and simulated hospitalisation incidence for each region, along with 95%, 90%, 75%, and 50% credibility intervals. The regions are ordered according to population. In the lower right panel, we have the national fit for the model assuming the same transmissibility in all counties. The incidence for the next three weeks not used in the calibration is provided in red.

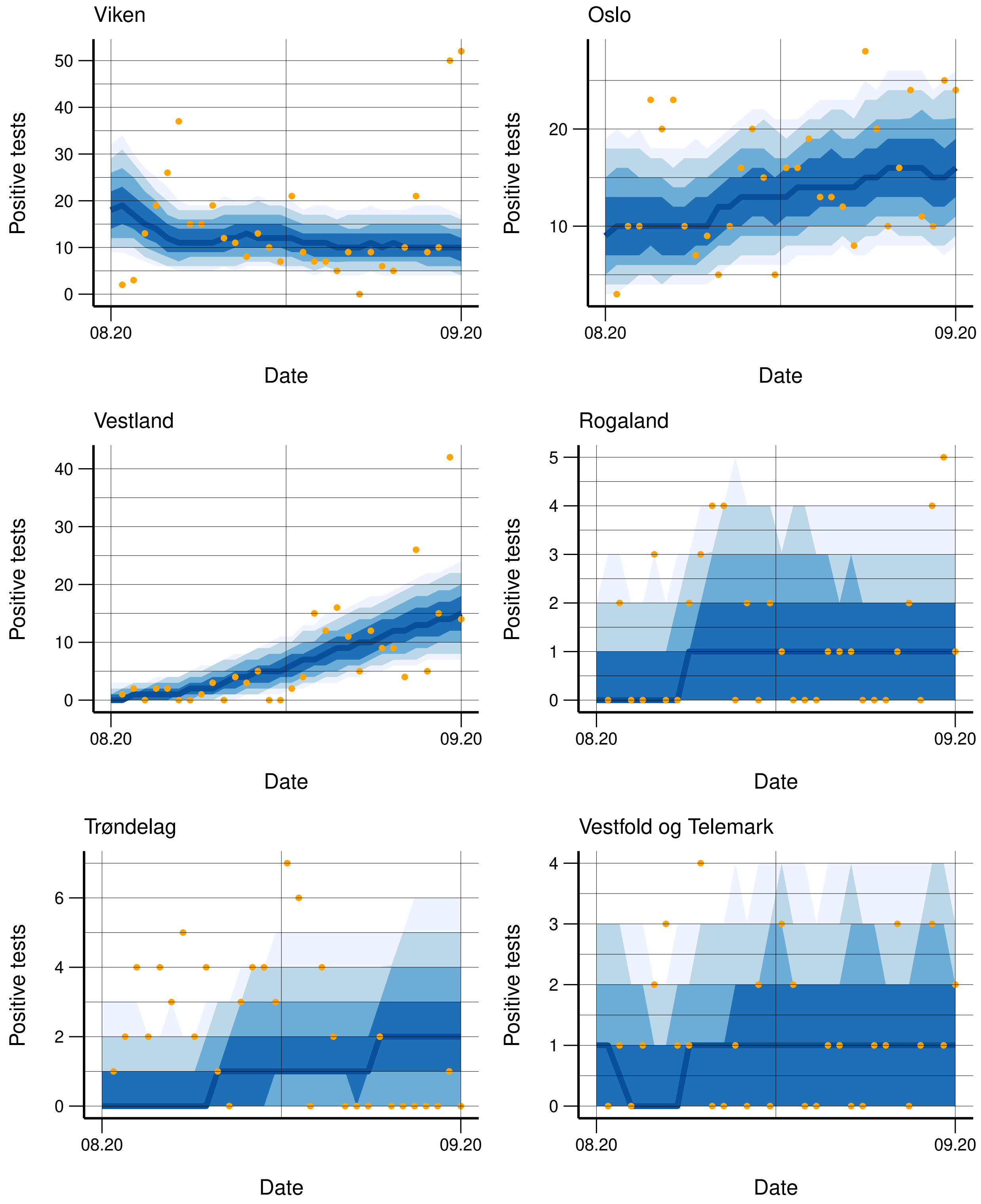

Fig AA: Observed (orange dots) and simulated positive tests for each region, along with 95%, 90%, 75%, and 50% credibility intervals. The regions are ordered according to population, with the capital Oslo as second.

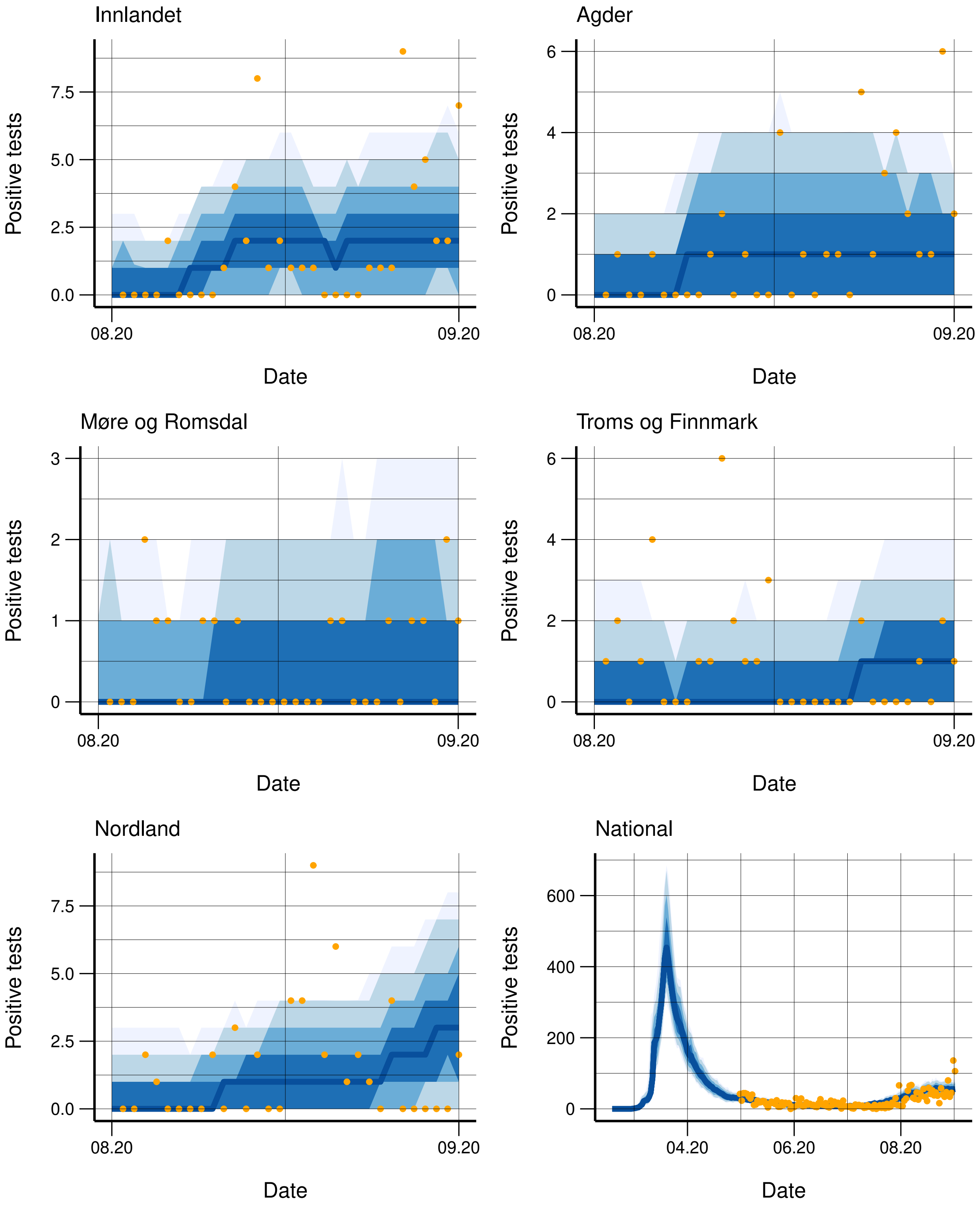

Fig AB: Observed (orange dots) and simulated positive tests for each region, along with 95%, 90%, 75%, and 50% credibility intervals. The regions are ordered according to population. In the lower right panel, we have the national fit for the model assuming the same transmissibility in all counties.

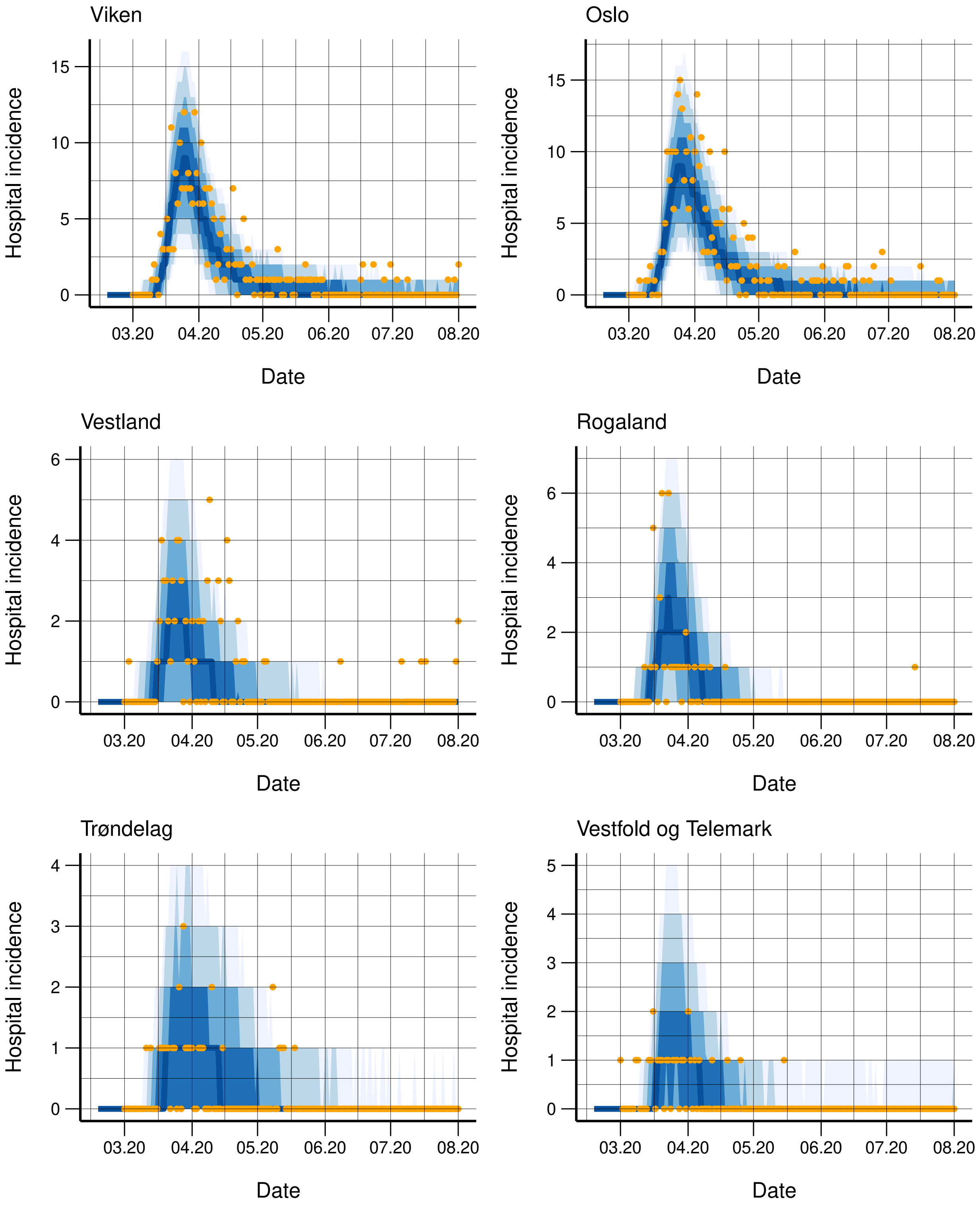

Fig AC: Observed (orange dots) and simulated hospitalisation incidence for each region, along with 95%, 90%, 75%, and 50% credibility intervals. The regions are ordered according to population.

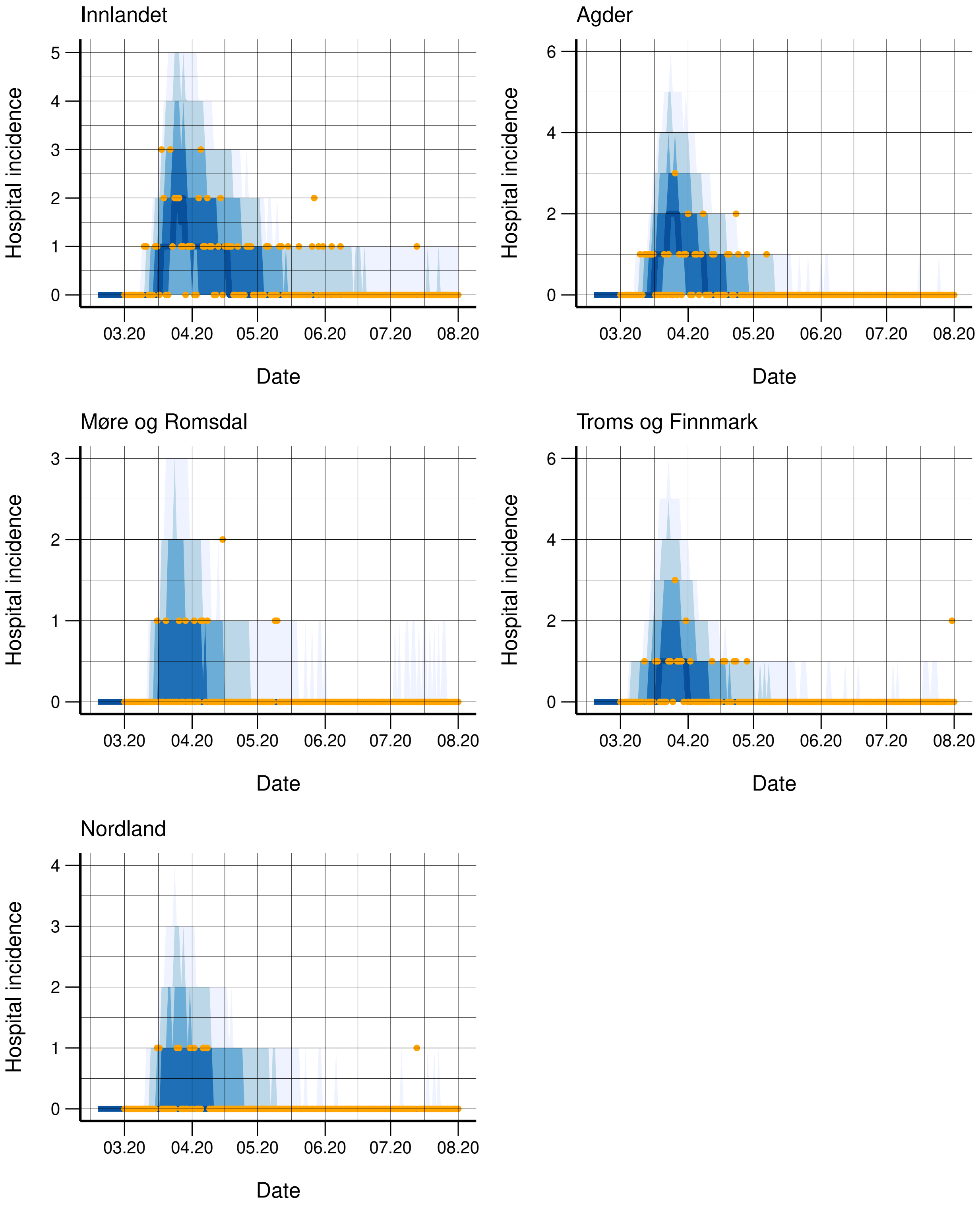

Fig AD: Observed (orange dots) and simulated hospitalisation incidence for each region, along with 95%, 90%, 75%, and 50% credibility intervals. The regions are ordered according to population.

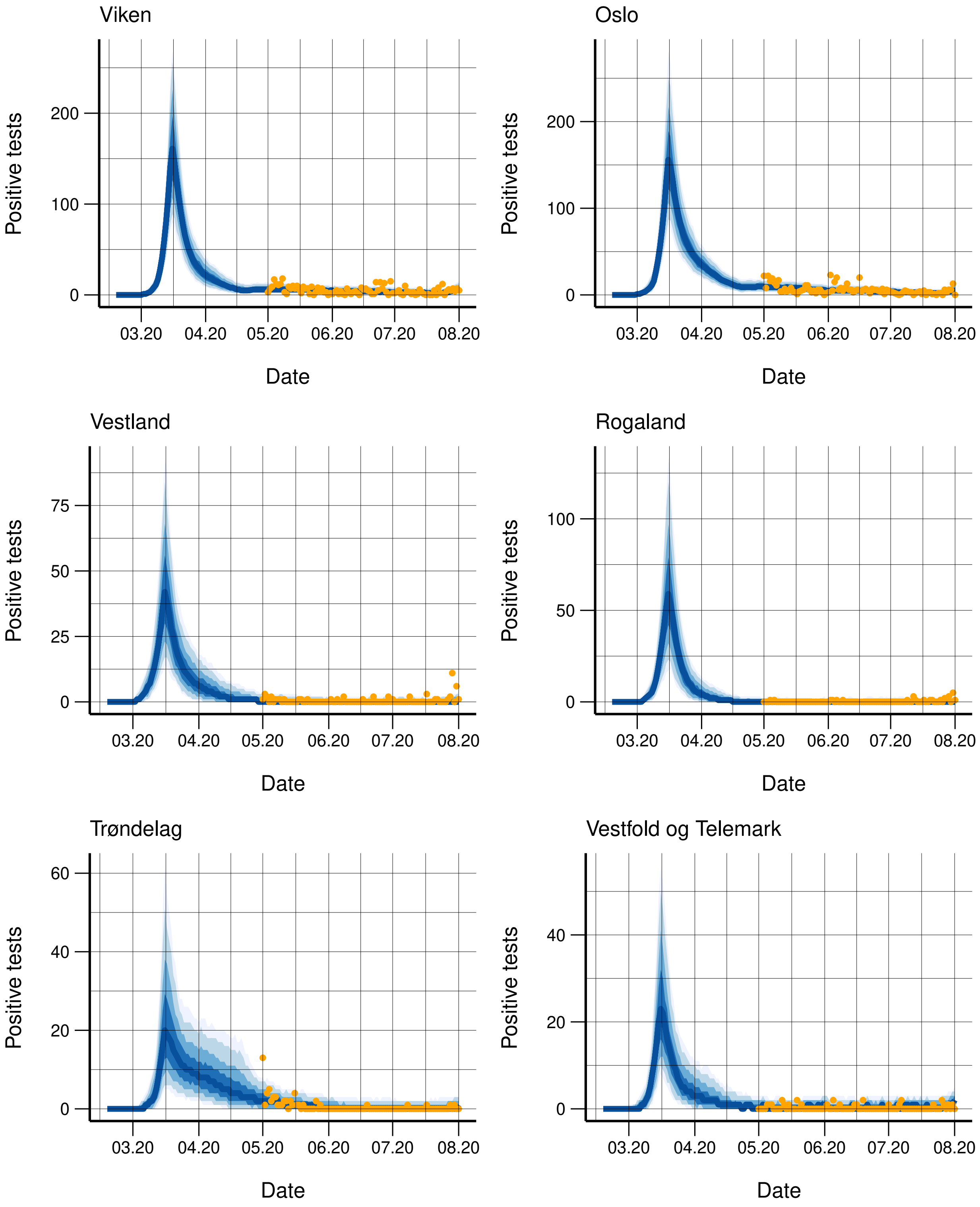

Fig AE: Observed (orange dots) and simulated positive tests for each region, along with 95%, 90%, 75%, and 50% credibility intervals. The regions are ordered according to population.

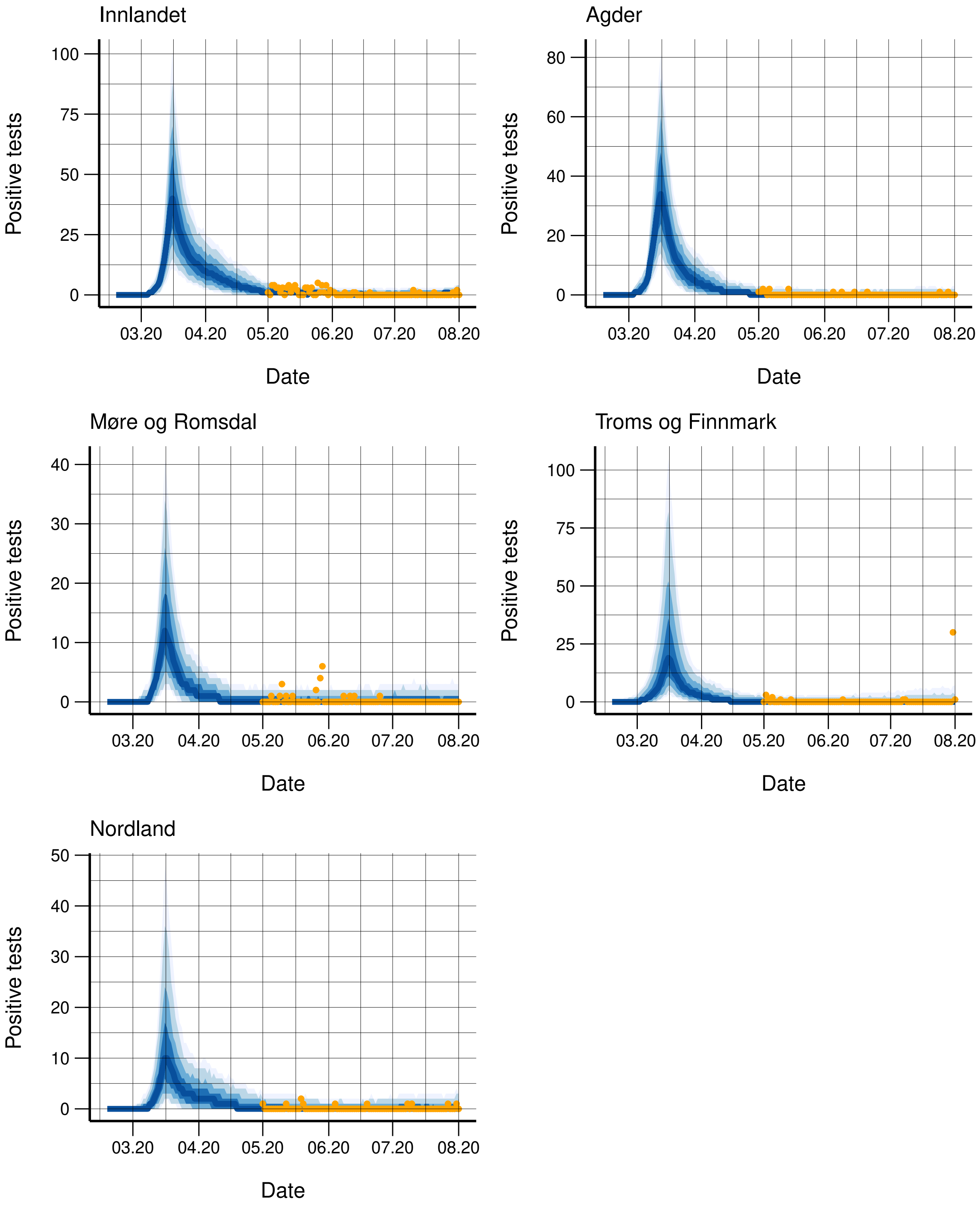

Fig AF: Observed (orange dots) and simulated positive tests for each region, along with 95%, 90%, 75%, and 50% credibility intervals. The regions are ordered according to population.

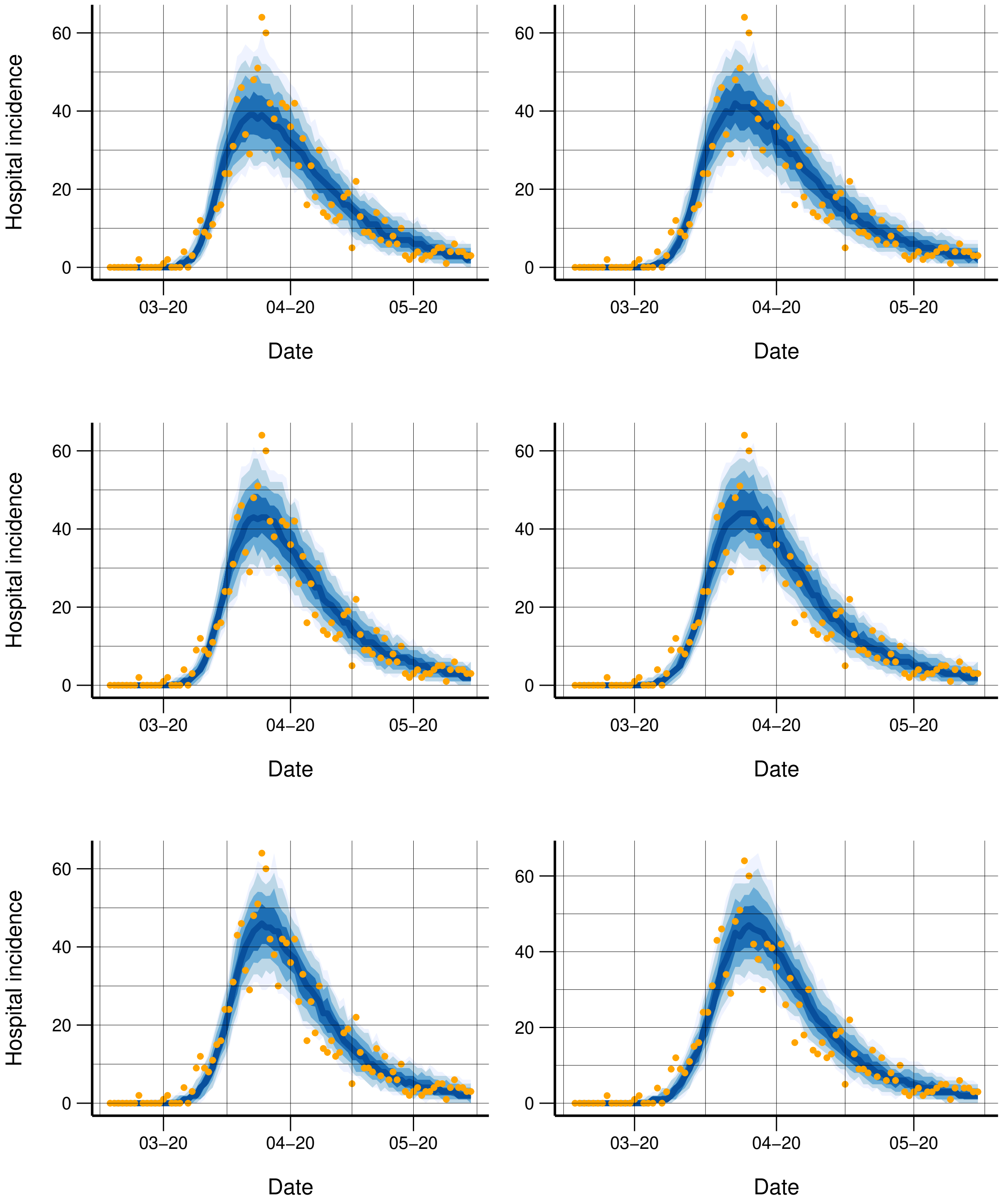

Fig AG: Observed (orange dots) and simulated hospitalisation incidence when varying the changepoint as March 11 (upper left), March 12 (upper right), March 13 (mid left), March 14 (mid right), March 15 (bottom left), and March 16 (bottom right).

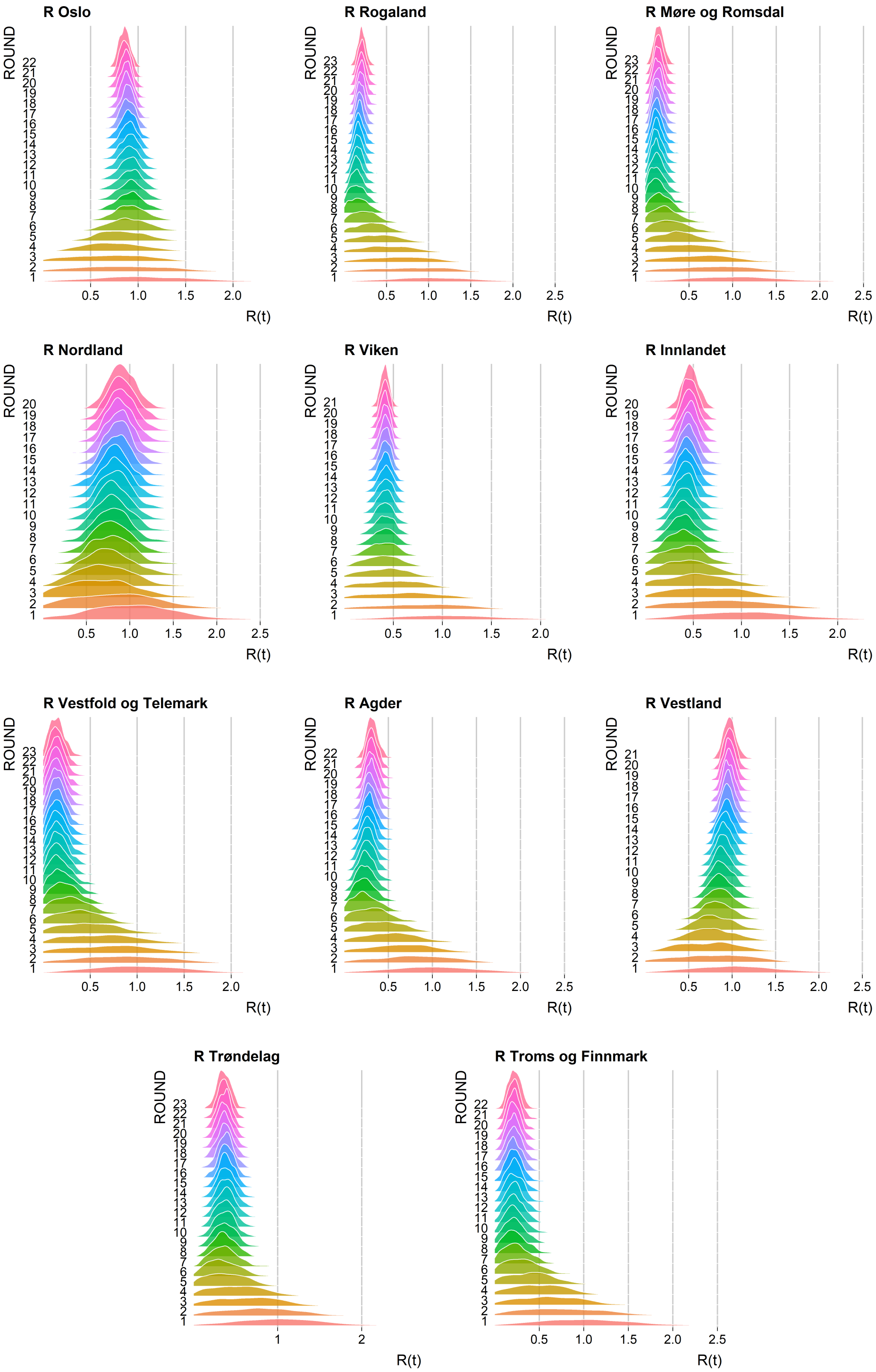

Fig AH: Example of ABC convergence sequences of the distributions of the estimated reproductive numbers throughout the calibration rounds (with data up until September 2020).

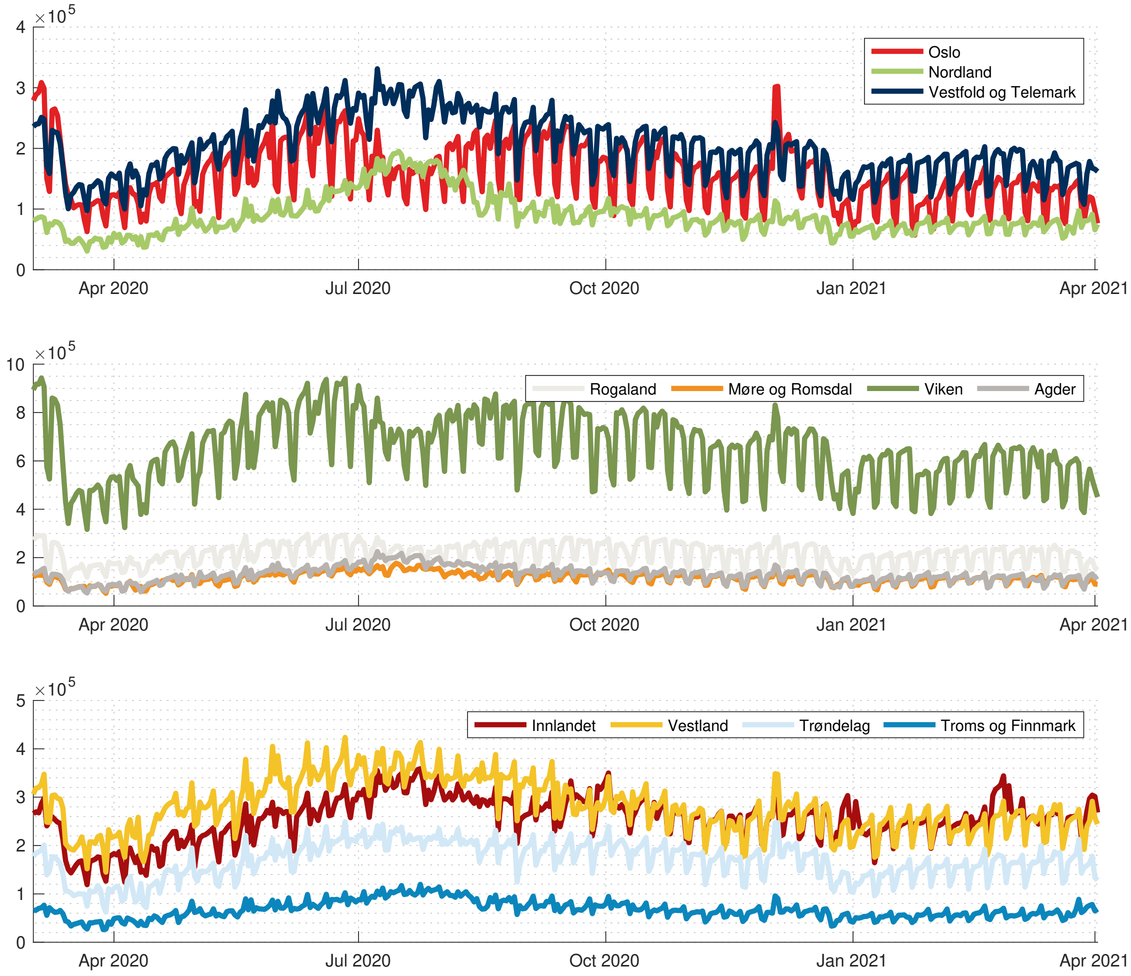

Fig AI: Daily time series of aggregated outgoing mobility from Norway's counties.

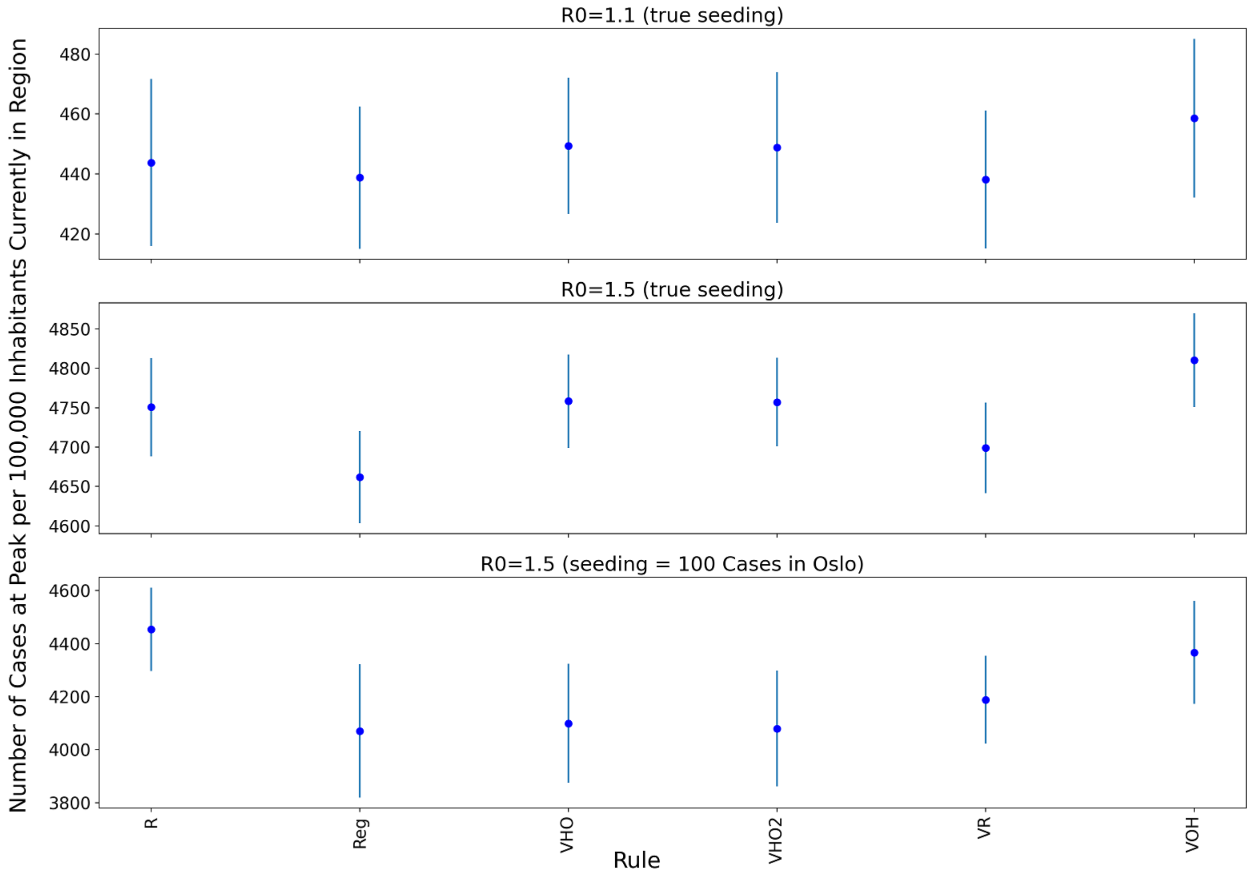

Fig AJ: National epidemic peak size with different mobility rules given different pandemic conditions, transmissibility and seeding. The plots show the mean and 95% CI based on 100 simulations for each mobility rule.

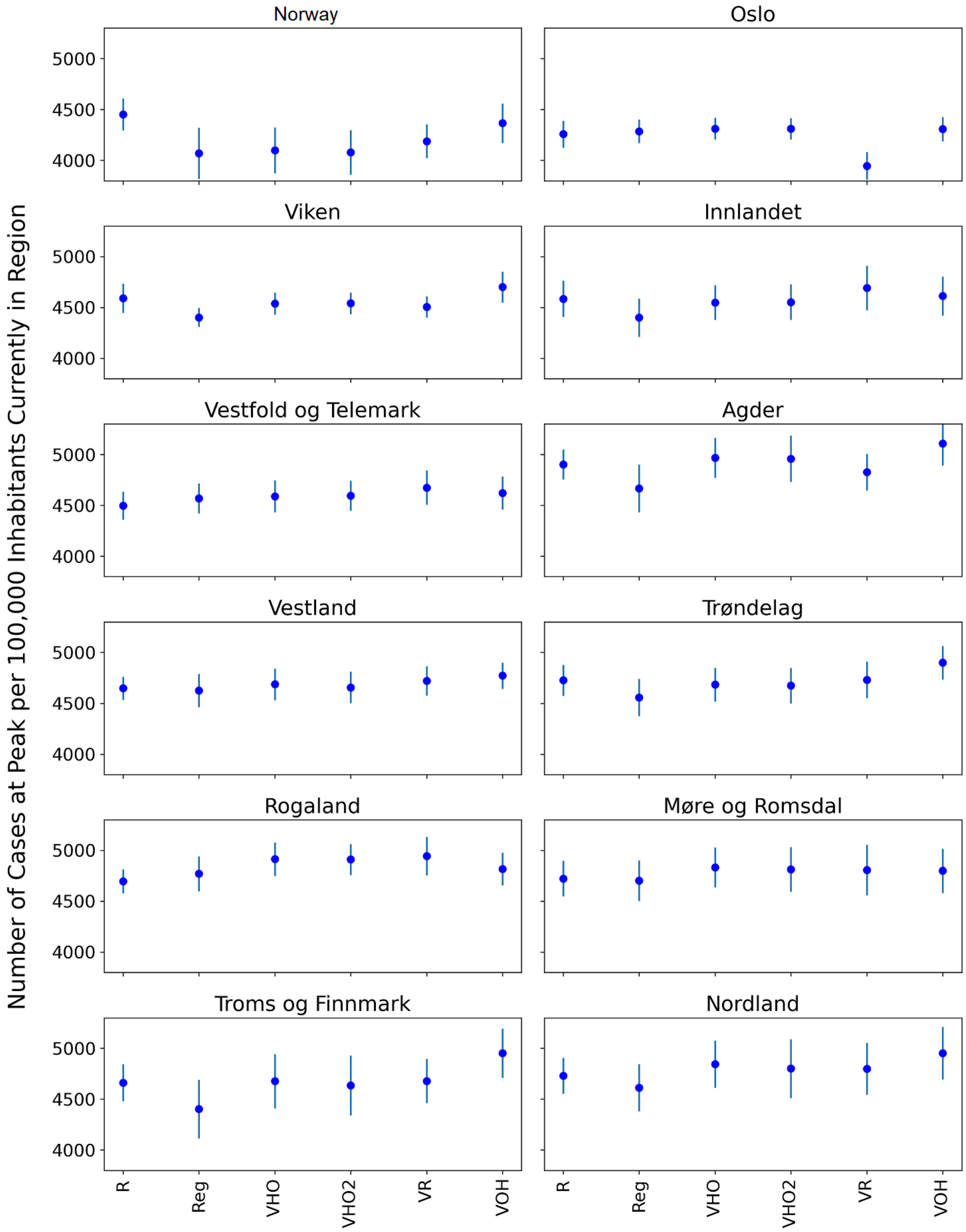

Fig AK: Epidemic peak size by county and nationally with different mobility rules, R0 = 1.5 and initial seeding of 100 cases in Oslo county. The plots show the mean with 95% CI based on 100 simulations for each mobility rule.

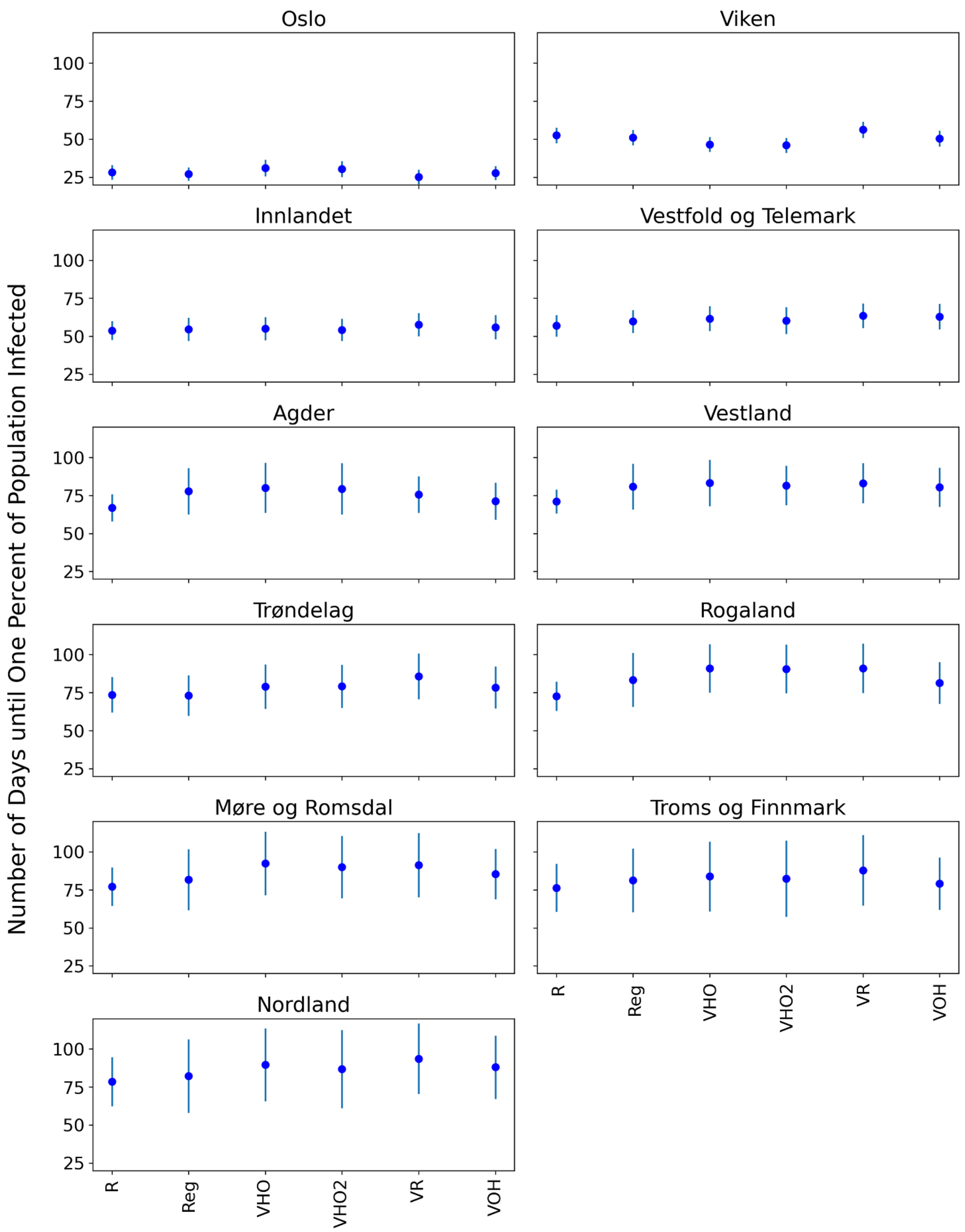

Fig AL: Time to one percent of resident population in each county is infected with different mobility rules, R0 = 1.5 and initial seeding of 100 cases in Oslo county. The plots show the mean with 95% CI based on 100 simulations for each mobility rule.

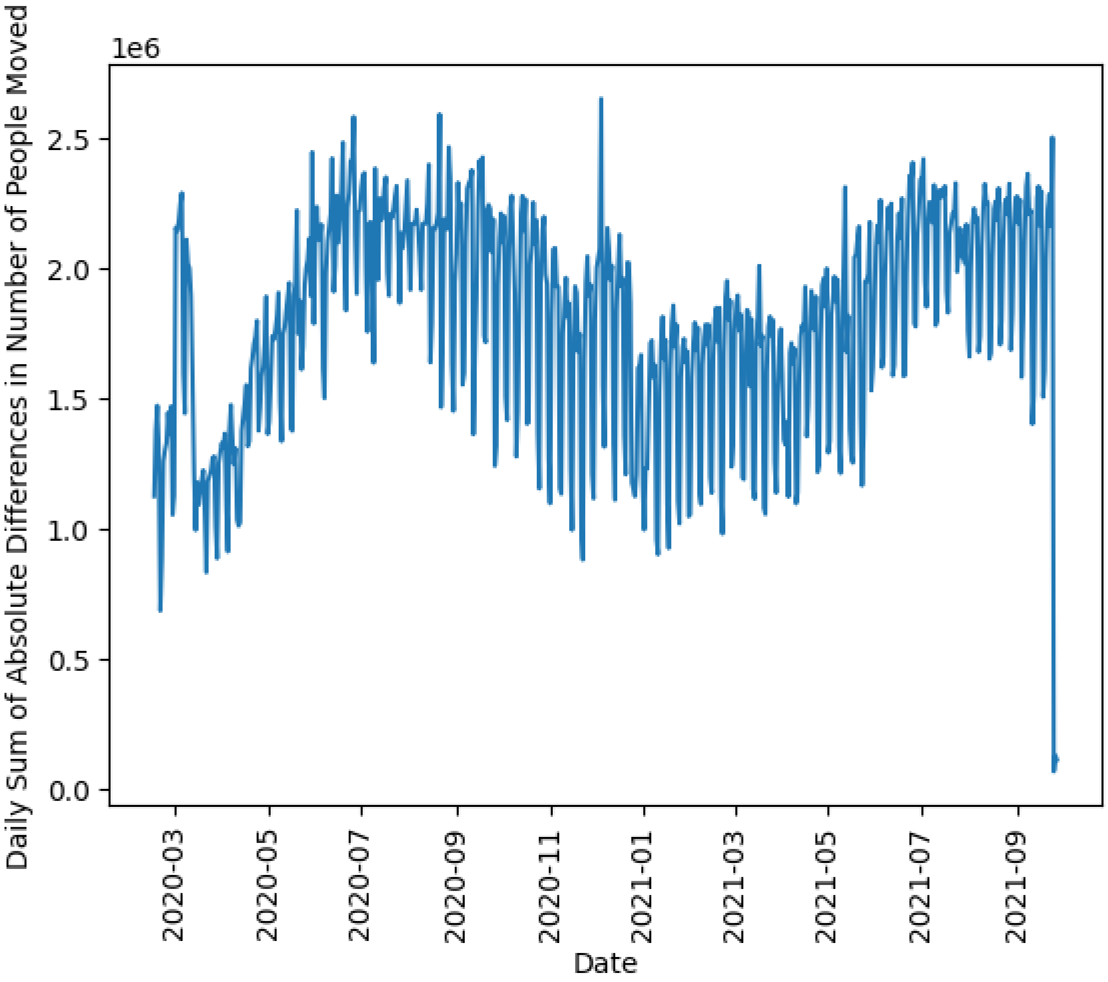

Fig AM: Daily absolute difference in inter-county movements for the regularised and original mobility matrices. To calculate the net movements between counties on a given day, we separately sum up the four daily regularised and original matrices at all non-diagonal entries, thus excluding movements within the same location.

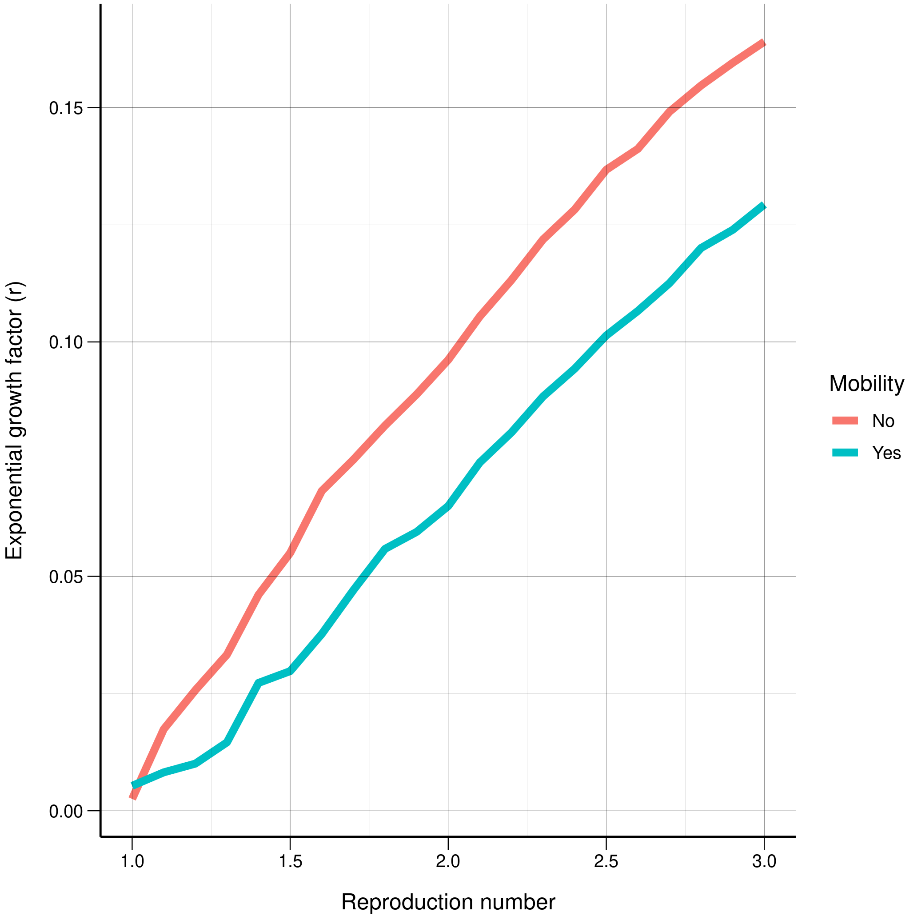

Fig AN: Estimated growth rate in Oslo as a function of increased reproduction number in Oslo. The reproduction number in all other counties is set to 1.

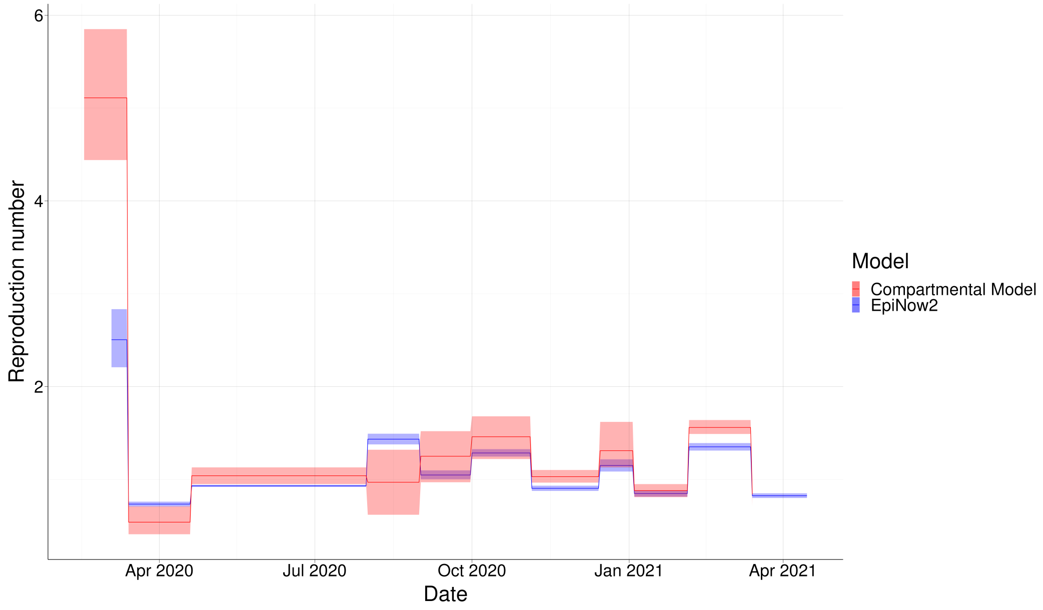

Fig AO: Estimated regional reproduction numbers for Oslo from the model presented in this paper (here named Compartmental model) compared to the estimated reproduction numbers from EpiNow2.

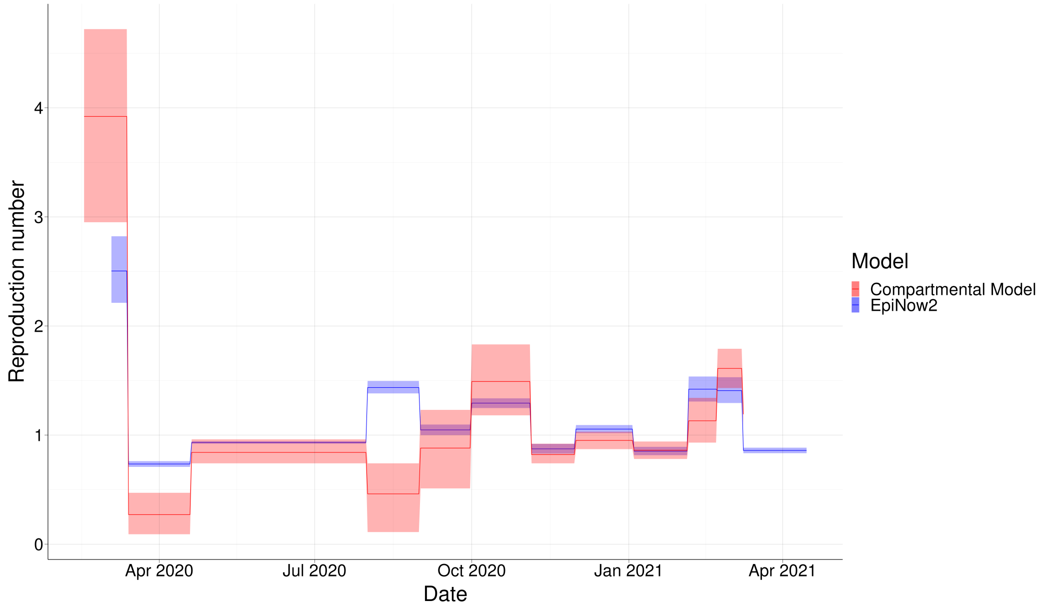

Fig AP: Estimated regional reproduction numbers for Viken from the model presented in this paper (here named Compartmental model) compared to the estimated reproduction numbers from EpiNow2.

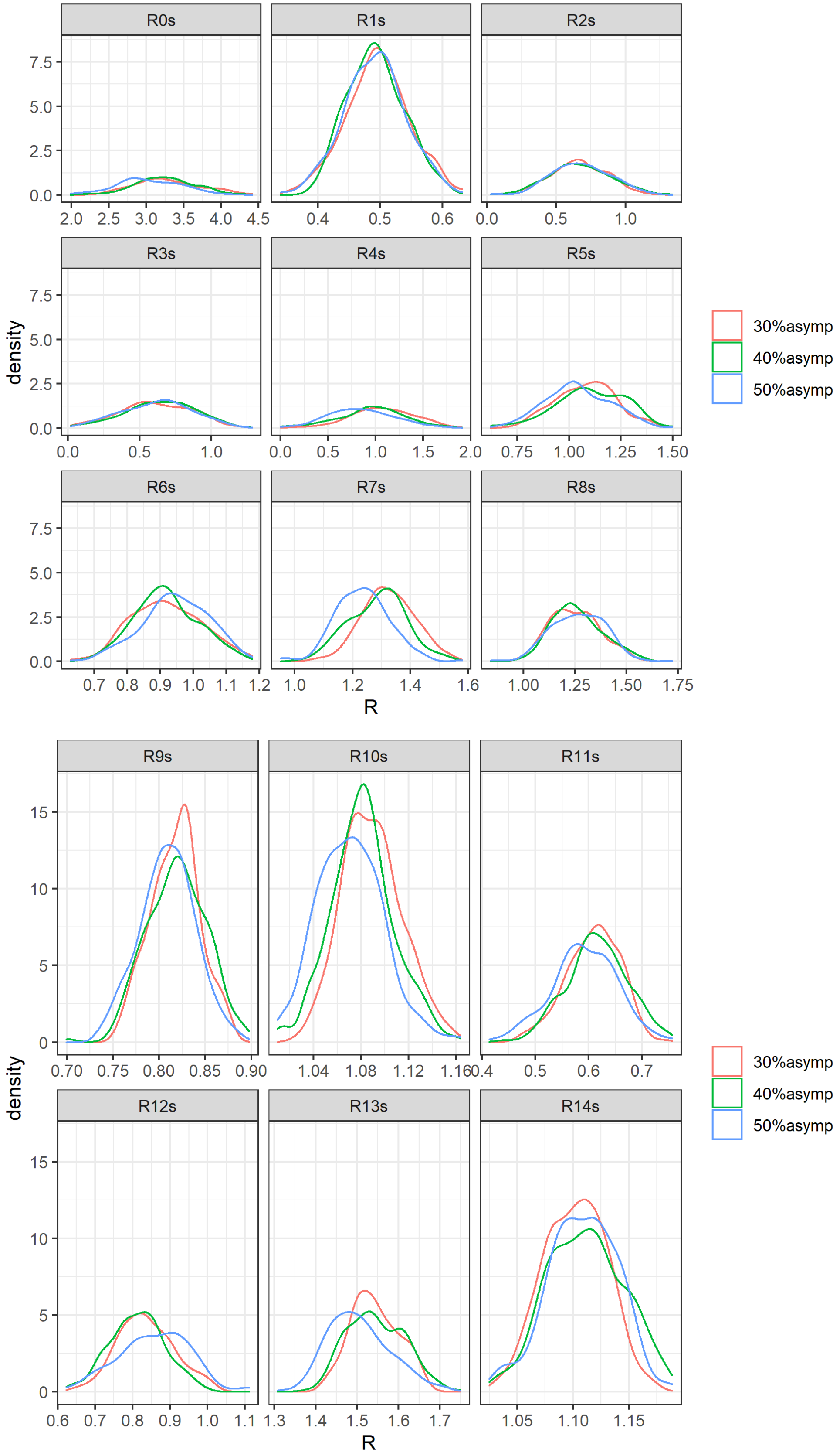

Fig AQ: Sensitivity analysis of the probability of asymptomatic infection.

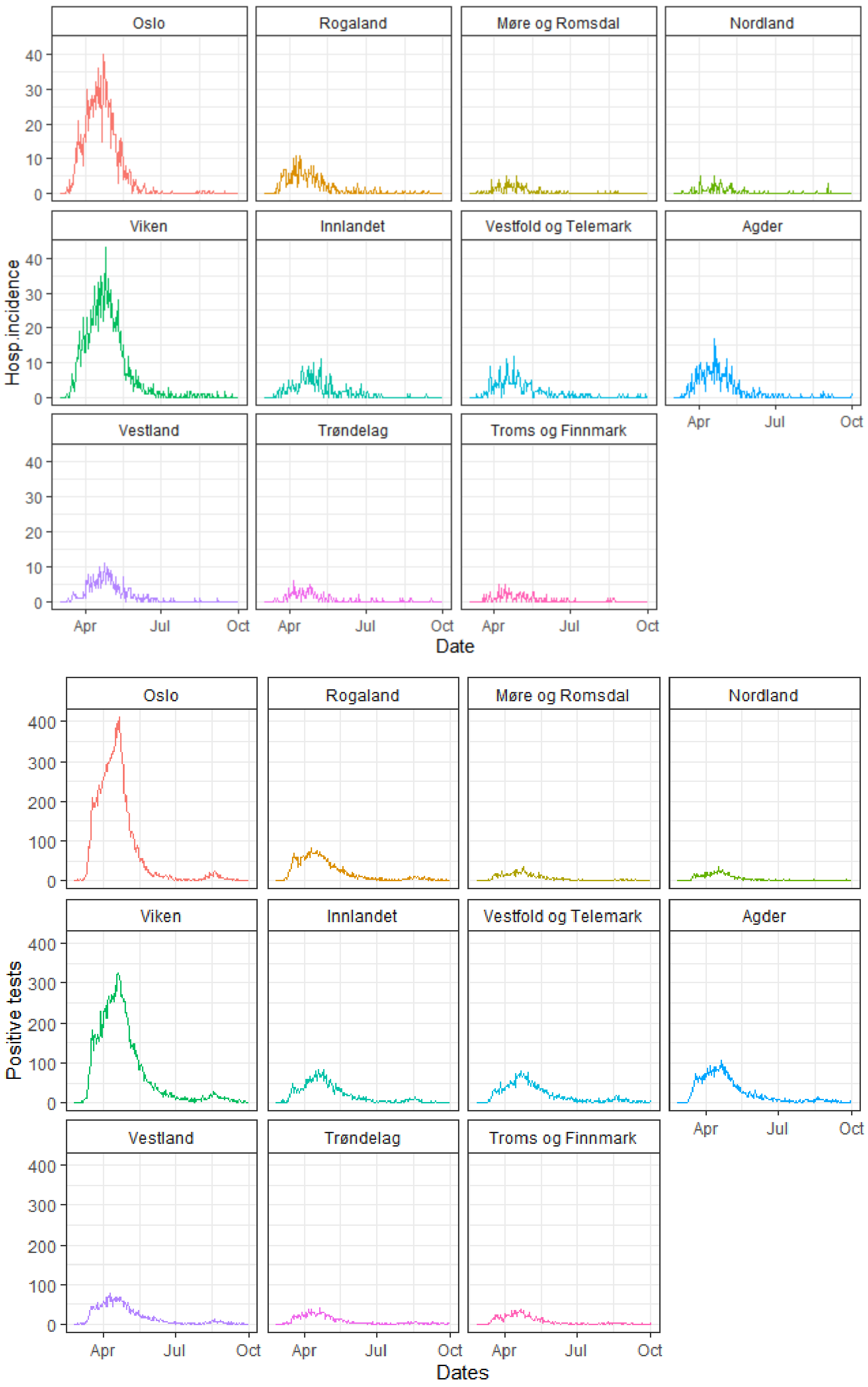

Fig AR: Synthetic data: hospital admissions (top) and positive tests (bottom).

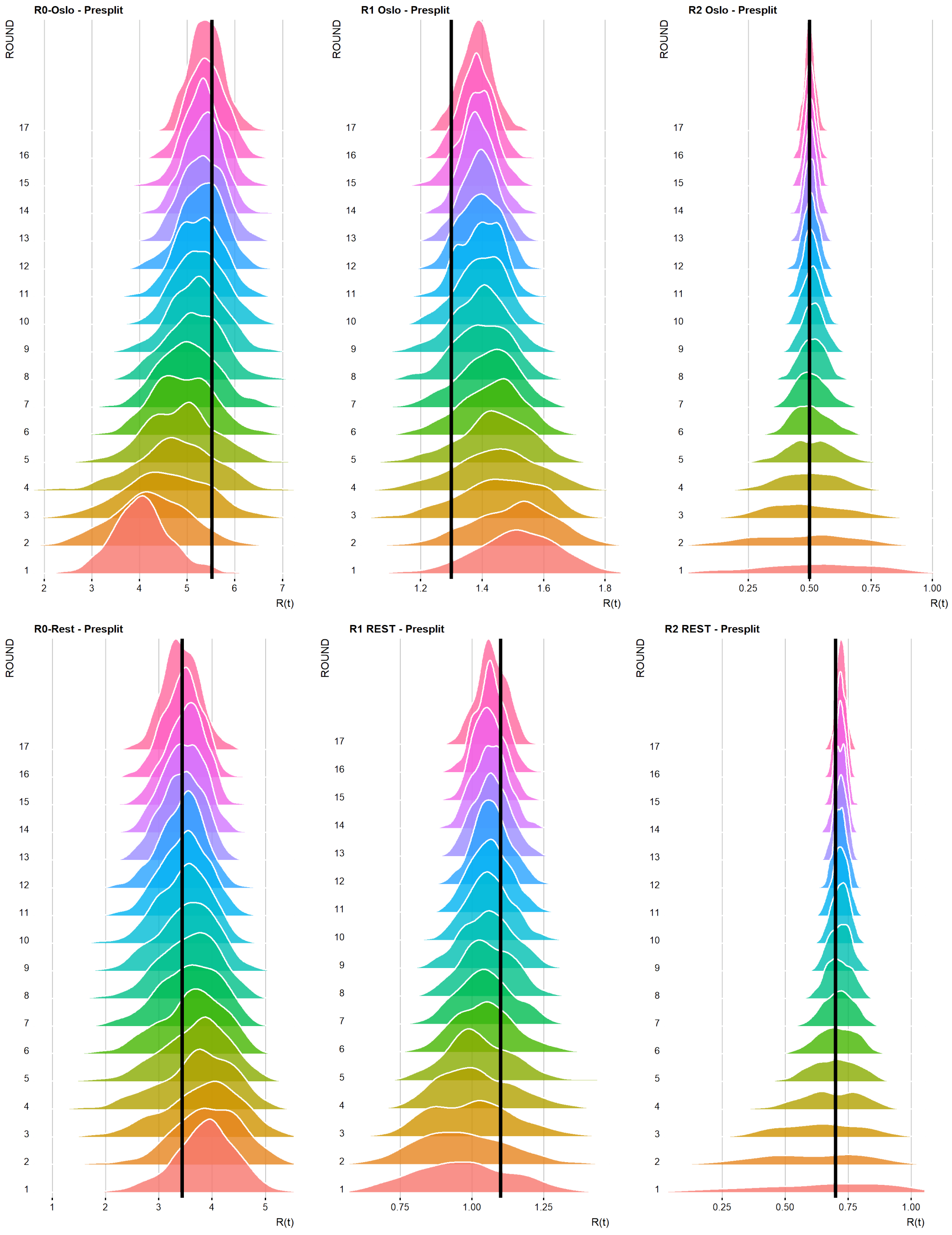

Fig AS: Convergence of split method towards the truth (black vertical line): Presplit

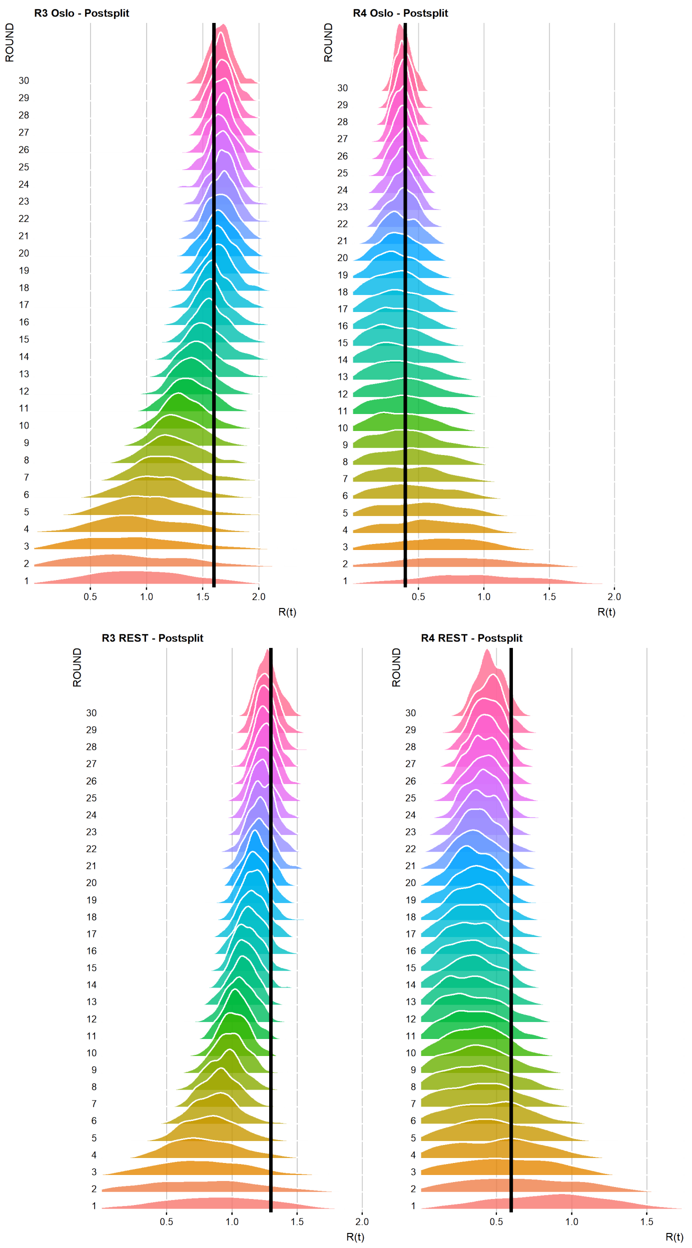

Fig AT: Convergence of split method towards the truth (black vertical line): Post-split
