# Supplementary material for: A real-time regional model for COVID-19: Probabilistic situational awareness and forecasting
Source: PLoS Comput Biol. 2023 Jan 23;19(1):e1010860. doi: 10.1371/journal.pcbi.1010860 (PMC9894546; doi:10.1371/journal.pcbi.1010860)
Supplement: Attachment — Submitted filename: ResponseLetter_PCB_110082022.pdf [file pcbi.1010860.s002.pdf]

Oslo, 11 August 20111

Dear Editor.

We thank you for giving us the opportunity to submit a revised manuscript *"A real-time model for COVID-19: probabilistic situational awareness and forecasting"* to PLOS Computational Biology. We also thank the four reviewers for the careful reading of the manuscript and their thoughtful suggestions, which have significantly improved the manuscript. We have been able to change the manuscript to account for almost all the points raised.

Please find below a point-to-point response to all comments by the reviewers.

On behalf of all authors,

Birgitte Freiesleben de Blasio

**Reviewer #1:**

The authors present a metapopulation model of SARS-CoV-2 transmission in Norway including the effect of human mobility from mobile phone data, informed with highly detailed information from individual-based surveillance data and calibrated to aggregated data on hospital incidence and notified cases. The model was actually used to support national decision making throughout the COVID-19 epidemic and in this paper it is applied retrospectively to the whole epidemic history up to April 1, 2021. The model is able to reproduce correctly the epidemic curves of hospitalized and confirmed cases by region, and to predict with good accuracy observed data up to three weeks after the end of the calibration period. The manuscript also presents an innovative and well thought algorithm for calibrating the many parameters of the model (mainly the changepoints in regional transmissibility following the initiation and termination of interventions). All in all, the manuscript is clearly written, the methods have been explained with detail and transparency, and the amount of work for its development and calibration is to be commended. Below are some of my comments which I hope can improve this interesting work.

* Parameters for the transmission dynamics (Table S3) were assumed from a preprint coming from the early phase of the pandemic when most of these values were still poorly quantified; furthermore, the purpose of that reference was to demonstrate the effectiveness of digital contact tracing via a theoretical model rather than to realistically describe observed epidemic dynamics. Some of the adopted parameters are not very credible with what is known now about COVID: in particular, the infectiousness of asymptomatic individuals being 1/10 of symptomatic individuals or the 40% probability of being asymptomatic are both very low. I think the authors should verify the impact of these assumptions with sensitivity analyses; I am not asking to recalibrate the model if this is a computationally intensive task, but to demonstrate in the supplementary material how the behavior of the model would change if more realistic parameter values were used. I also recommend finding support for the other parameters used (lengths of incubation/presymptomatic/infectious periods) from relevant epidemiological studies.

***ANSWER:*** *Thank you for making this important point. Even at this stage, the probability of being asymptomatic varies hugely in the existing literature, e.g.:*

- *19.9% (95% CI: 18.8-21.1%): https://www.hindawi.com/journals/bmri/2022/3401566/*
- *82-87%: https://www.pnas.org/doi/10.1073/pnas.2019716118*
- *40.5% (95% CI, 33.50%-47.5%) among the confirmed population: https://jamanetwork.com/journals/jamanetworkopen/fullarticle/2787098*
- *No significant difference in proportions of symptomatic and asymptomatic index cases was seen (50%): https://www.sciencedirect.com/science/article/pii/S1201971221001867*

*So in this respect, we are not convinced that 40% is too low. But, as suggested, we have included a sensitivity analysis of the asymptomatic proportion in the SI Text. We have done this in the national calibration model, repeating the analysis assuming a 0.3 and 0.5 probability of asymptomatic infection. The analysis shows that the estimated reproduction numbers were robust with respect to these differences. We added the analysis to the SI Text , see Section S2. 9.*

*The parameters for the duration of the preinfectious and infectious states in our model are based on the paper by Ferretti et al. as referenced in the SI Text Section S1.3. A systematic review by Alene et al. (BMC Infect Dis.. 2021 Mar 11;21(1):257. doi: 10.1186/s12879-021-05950-x) finds a mean serial interval in the range 4.2-7.5 days, and a weighted pooled mean estimate of 5.2 days (95%CI: 4.9-5.5) days. In comparison, our generation time is close to the upper interval of the reported serial intervals. We have updated the disease state duration parameters in our weekly situational awareness model in accordance with more recent literature. However, for this paper we have chosen to use the original model parameters to document our approach and the results as they were reported during 2020 and the first part of 2021.*

* The definition of the model for the detection probability (lines 311+) is quite hard to assess. If I understand correctly, the authors assume that the fraction of detected cases depends logistically on the number of tests performed; however, this does not take into account the fact that the scale-up in the number of tests could not always keep up with the rise in the number of infections (as shown by the positivity test ratio usually going up with new epidemic waves). As a consequence, a detection rate of 55-70% (Figure 2) seems extremely high even for the high public health standards of Norway; this ratio has been estimated at about 10% for Italy and France (Marziano et al, PNAS 2021, Pullano et al, Nature, 2021) in the early phase of the epidemic and to not exceed 40% throughout 2020, even when the incidence was low and the logistic of testing had been fine-tuned.

***ANSWER:*** *We agree that this is a very simplified model, and that it could well be that the probability of detecting a case should also depend on the number of positive cases. Remember that we use three main sources of data, namely the daily incidence of covid hospitalisations, the number of daily lab-confirmed positives and the total number of daily tests performed. With this data it is very difficult to get good estimates of factors that contribute to the detection probability. We were not able to estimate a model with more parameters than the current two for the detection probability. By allowing a logistic dependence on the number of tests, we capture how the detection probability depends on the testing capacity. We would have liked to study this relationship more thoroughly, however, unfortunately we do not have good data for Norway to inform us about it: we believe that one would need longitudinal seroprevalence data to properly study the detection probability. As a reference, there is one seroprevalence study for Norway (https://www.eurosurveillance.org/content/10.2807/1560-7917.ES.2022.27.13.2100376 ) where a detection probability of almost 90% is estimated, which is higher than our estimated 55-70%. We believe that the parameter which mostly affects the estimated detection probability is the hospitalisation risk in Norway. Again, without seroprevalence studies, we do not have good data to*

*inform us about this parameter, and this is a limitation. We have added the following to the paragraph on the detection probability:*
*Note that this is a very simple model. For example, one may argue that the probability of detecting a positive case should also depend on the total number infected (not only through the total number of tests). However, without additional and more specific data able to inform about the detection probability (e.g. seroprevalence data), we were not able to estimate additional parameters.*

* The estimated values of the basic reproduction numbers (Table 1) seem very high compared to literature values of 2.5-3. The higher effectiveness found for the lockdown in Norway, compared to estimates from other countries, may derive from an overestimation of the R0. Furthermore, where regional analysis have been conducted, limited variability in both the basic reproduction number and the effectiveness of lockdown was observed (e.g., [Guzzetta et al., EID, 2021]), whereas in this analysis the variability seems significant. Did the authors try to compute the net reproduction number from the epidemic curves at the beginning of the epidemic [Cori et al, AJE 2013], to see how they compare with model-derived estimates? Can the authors discuss their results and provide hypothesis on why would the R0 in Norway be almost 25% higher than in most other countries and the one in Oslo as high as 5.1?

***ANSWER:*** *We agree that the estimated basic reproduction number is somewhat high compared to estimates from other countries. There can be multiple reasons for this. One likely reason is an underestimation of importation, underestimation of hospitalisation probability or a slightly too long generation time. Comparing to reproduction numbers estimated from early case-counts in Norway is a very difficult exercise due to very low testing activity and frequently changing testing criteria. We have added one sentence in the manuscript commenting on this. For Oslo, which has the highest reproduction number, there is an additional factor in that the model includes a significant outflow of infection from Oslo due to the mobility. This leads to a higher estimated reproduction number than one would naively assume from the slope of hospitalisations or cases. We have added a section in the SI Text, Section 2.8 to illustrate this effect. In addition, we have added the following sentence in the Discussion:*

*"Note that the estimated reproduction numbers are on the high side of the estimated reproduction numbers for other countries (Billah et al. 2020), which could be due to for example an underestimation of importation, underestimated hospitalisation risks, longer assumed generation time, or due to mobility, as illustrated in S1 Text section S2.8".*

* The conclusion at line 673-674 that "the reopening of kindergartens and schools did not increase transmissibility significantly" needs to be more explicitly contextualized to the lockdown effectiveness in Norway, otherwise it risks to be misinterpreted as a proof of the lack of importance of kindergartens and schools for transmission. Even in the author's analysis, the reopening contributed with an increase of 0.17 (i.e. 35%), in the reproduction number after April 20, 2020 (Table 2); this increase may not be important when starting from a situation of transmission significantly below threshold, but can cause large epidemics if the transmissibility before reopening is close to the epidemic threshold.

***ANSWER:*** *Agree. We have changed the sentences to:*
*"Interestingly, we found that the reopening of kindergartens and schools did not coincide with a significant increase in the reproduction numbers in Norway. Note however that a small increase in the reproduction number may lead to a large increase in the number of cases in a setting close to the epidemic threshold".*

* lines 726-728: please note that at least another attempt was previously published early on in the pandemic (Gatto et al., PNAS 2020), so consider revising this sentence.

***ANSWER:*** *The model by Gatto et al. uses 2011 commuting data from Italy, which is different from our approach of using real-time mobility data. Containment measures have strongly affected mobility patterns during the pandemic. We have altered the sentence in the Discussion to emphasize the difference between real-time vs historical mobility data and added the suggested reference as an example of previous works that employ the latter. It now states: "While there are published SARS-CoV-2 models informed by historical mobility data, e.g. ref Gatto et al., using commuting data from 2011, to the best of our knowledge, our modelling approach is first to employ real-time national mobility data with fine geographical resolution".*

* The manuscript is very verbose; A large part of methodological explanations should be moved to the Supplementary information.

***ANSWER:*** *We have moved most of pages 12 and 13 to the SI Text, as also suggested by one of the other reviewers. We have chosen to keep the rest of the methodological explanations, as we believe this is a key contribution of the manuscript.*

**Reviewer #2:**

The paper presents an adapted algorithm for implementing SMC-ABC methods for fitting a discrete-time stochastic epidemic model stratified by regions. The model integrates complex data on movements between regions based on mobile phone data to capture the spread of transmission between regions and to improve the ability to make short-term predictions on the epidemic spread. The model has clearly been in use throughout the COVID pandemic and has been guiding/informing public policy throughout. It's important that such models achieve good visibility with high impact publication so that there is public trust and transparency in the work being done to inform government.

My main concerns regarding the paper are that model has lots of facets and interesting features, that have been largely summed up through written descriptions that in parts can be quite obscure, confusing and difficult to follow. I strongly suspect some of my concerns below would be addressed through greater clarity of explanation, model figures and maybe some extra technical detail in the supplementary information.

Main concerns

1. The calculation of the hospitalisation risk is very confusing in the manuscript. The is obviously a quantity that is highly heterogeneous over age and with the lack of age dependence the authors are correct to try and consider the constitution of the different regional populations. The authors use age-specific estimates for hospitalisation risk from literature and then, for each region, uses a weighted average of these. The text (in the first paragraph of the section) seems to suggest that the weighting is derived based on demography, before, in the second paragraph of the section, suggesting that the weighting is based on the age distribution of the cases. This latter weighting seems to make more sense, but I found these two paragraphs to be contradictory. Could it be different treatment of the symptomatic and asymptomatic infections?

***ANSWER:*** We agree that the explanation was unclear. Demography is used for the periods where we do not use the test data (i.e. before May 1 2020). Otherwise, the weighting is based on the age distribution of the cases. The text in the *Model for admission to hospital and intensive care* section now reads: *"The hospitalisation risks were based on the age-specific estimated hospitalisation probabilities in Salje et al. 2020. Even though the compartmental model did not have age compartments, we needed to take into account age when computing hospitalisations, as the probability of requiring hospitalisation was highly dependent on age. We corrected the hospital admission probabilities to account for age-dependencies in transmission, by adjusting for the age distribution of the positive cases. For the period where we do not use the test data (prior to May 1, 2020), we computed the probability of hospitalisation by taking into account the demographic age profile in each region. The details on the delay distributions and hospitalisation risks are provided in S1 Text Section S1.2."*
*As the reference for the hospitalisation risks provided the probability of hospitalisation per infection (not separating between symptomatic and asymptomatic infection), we do not use separate probabilities for the two. However, in practice there is of course likely a difference.*

2. Fitting of the regional model. The authors appear to make multiple use of the data, estimating the 'static' parameters (d, pi1, pi2, amplification) based on the national model before then disaggregating the data and then estimating the transmission parameters in the regional model assuming a prior for these parameters based on the first stage posterior. This appears, as written, to be a multiple use of the data overweighting the information it contains. Is this a fair reflection? Is there not some way that the data could be partitioned so that a double use of the data is not being made (i.e. estimate one based on hospitalisations, and the other based on test positive cases)?

***ANSWER**: As these parameters have the same definition in the regional and in the national models, we have done this to speed up convergence of the regional model as a pragmatic choice. We agree that this might affect the uncertainty of the predictions (and estimates). However, we do find rather good coverage of the regional predictions (cf. Fig 5) and are thus not worried about this effect.*

3. The time from transmission to positive test is a prior a uniform rv over the range [0,4] days. Is this not VERY short, considering the expected incubation period a priori is 5.1 days? Would suggest that most people are testing positive before they've even got symptoms. Can this be so? Would it not be better to count d as the mean time between transmission and test positive and have some kind of convolution over this distribution (it doesn't have to introduce extra stochasticity) and would smooth out the projected curve of case counts.

***ANSWER:** Thank you for pointing this out. The text was unclear. It should be the time between entering the infectious symptomatic class (I) or infectious asymptomatic class (I_a) and positive test. We have corrected the sentence in the Calibration section, which now reads: " [..], the delay between entering the infectious symptomatic or asymptomatic class and a positive test, which was assumed to be a uniform integer between 0 and 4 days".*

4. The algorithm doesn't say what happens if the simulated data fall outside of the epsilon thresholds. I think it should say that you keep simulating until something does lie within the specified bounds, but there is a missing else statement. I guess also the definition of the weights doesn't need the case r = 0.

***ANSWER:*** *The while loop continues until i=1000. Hence, it restarts when the simulated data fall outside of the epsilon thresholds. It is correct that the weights are not necessary for r=0, and it should instead be r=1. We have corrected this in Algoritm 1 ABC-SMC in the Calibration section.*

5. As mentioned in the intro above, mathematical descriptions of some of the model features, such as the hospitalisation risk mentioned above, in the supplementary information would be very welcome.

***ANSWER:*** *We have added a mathematical description of the hospitalisation risk to the S1 Text, Section 1.2 material as suggested.*

6. I may have missed any discussion on the availability of data. I can see there is a github repository for the code, which, as ever, is welcome.

***ANSWER****: We have added more information about data availability to the manuscript.*

(Very) Minor comment
5. I felt the paper was overselling itself in describing a novel way of accounting for vaccination.. many works before have had time-dependent betas mopping up all sorts of fluctuating transmission behaviour etc...

***ANSWER:*** *We agree and have taken the comment into consideration and removed the sentence "Similarly, our approach copes with vaccination in an innovative way" in the Discussion section.*

**Reviewer #3:**

This is a rather long manuscript detailing (a) the use of a multi-region SEIR model for COVID-19, (b) how the authors overcome the parameter estimation problem by using a sequential approximate Bayesian computation approach, and (c) how they went on to use the methods on modeling roughly the first year of COVID-19 pandemic in Norway. The model described here seems interesting and useful, especially on the part where they incorporate mobility information. The manuscript went through great details on how to estimate parameters from the data for their models and make the model works, which honestly is a tough part of adding complexity to infectious disease model. I believe this manuscript provides significant methodological insight and will serve as a useful resource for future modelers. However, I can't recommend the manuscript for publication in its current form, and I believe a major revision of the manuscript is needed here, due to the poorly defined objectives, weak organization of the paper and communication about the model.

The manuscript is rather lacking in clear objectives. It is hard to know what is the novel part that you introduced to your model, based on the writing. Is the manuscript about making a multi-region SEIR model and that regions are interacting with each others and yet each region is separately parameterised? Is the manuscript about the "Split-SMC-ABC" that is particularly important in estimating parameters? Is the manuscript about showing how various regions in Norway experienced reduction of R values? Without clear objectives and questions that you are trying to answer, the results section looks like a mishmash of findings without clear thread and message. For example, why should the reader care about 3-week ahead prediction while also looking at how the R values have changed over time? Are the changes in R values over time in Norway going to help me understand if the model is a good choice? What can I learn from the regional mobility time series in relation to the

objectives? I think the abstract is much better in framing the objectives than the manuscript itself. And I think you should organize the story around the objectives.

***ANSWER:*** *We understand that the manuscript required more focus in many parts and therefore we implemented a careful re-organisation. We take the critique raised by reviewer to heart and have made major alterations to the manuscript to improve the readability and clarity of the text. This will be explained specifically in several points raised by all four referees. We moved material into the SI Text, we moved material within the main paper, we shortened but also added text for clarification. In the introduction we added a new paragraph, highlighting the objectives and detailing the organisation of the paper:*
*"In this study, our objective was three-fold: (i) to present the new regional model and our inferential approach; (ii) to report on the weekly epidemiological situation during 2020-2021 in Norway, where the model was used to inform real-time policy decisions, and (iii) to compare and validate our modelling approach. The article is organised in the following way. First, we provide a technical presentation of our regional model, including a model for using real-time mobility data, and a novel split-ABC-SMC inference technique. In the result sections, we document the implementation of the model with Norwegian data and report on county-specific effective reproduction numbers, alongside national results obtained from an alternative model ("null model"), excluding regional differences in the transmissibility. We present short-term forecasting results of hospital admissions and test data in the counties, obtained at different pandemic stages, covering highly volatile and relatively stable periods. Finally, we document the enhanced predictive performance of the regional model by comparing its projections to those obtained from a national null model and a simple regional model forecasting from the past two-weeks data."*
*We have restructured the Materials and Methods, Results and Discussion sections by 1) moving parts to the supplement, 2) re-organising the text to streamline the message, 3) changing the sub-sectioning.*

Although you talked about mobility data and how you analyse them at length, it wasn't clear to me how the mobility matrices were incorporated into the regional transmission model you formulated. You mentioned how people are moved from one region to another in the paragraph starting in Line 162, yet in the set of equations describing their local SEIR model, there was no mention about the movement of people among the locations. This is confusing, especially when I try to understand who are the people visiting other locality. Did you randomly move the people regardless of their state (S, E, I, R) and what are the justification for that? There is a lot of disconnect between the mobility part of the model with the local transmission part of the model.

***ANSWER:*** *We have clarified further the connection between the local transmissibility model and the mobility by explicitly defining N_i^t through the mobility matrices X_ij^t in the Local stochastic epidemiological model section.*
*In addition, we have added a specification that we move individuals regardless of their disease state. We chose to do this as we do not know the infection status of the individuals in the mobility data. We agree that it is reasonable to assume that symptomatic individuals might move less, in particular if the symptoms are severe. We have still chosen to move them randomly independent of disease status as one would otherwise have to make quite strict ad-hoc assumptions where we do not have any data. The added specification to the Regional transmission model section is: "We moved individuals independently of their disease status. Another option would have been to decrease the probability of travel for e.g. the infectious symptomatic, however we have chosen not to do this, as it would require ad hoc assumptions on the size of reduction".*

As with the results section, the discussion also feels like a mishmash of ideas. You seem to interweave future direction, limitation, the good and bad about the model, and sometimes tangential findings or "tips" altogether. This is very well tied into the problem of lack of general sense

of objectives. As a reader, I simply could not tell from the discussions if using the model is a good idea. In fact, I don't even quite understand what is exactly novel in the model already. I would again note that the abstract is better at selling the uniqueness of your model than the whole discussion section: "Our approach is the first regional changepoint stochastic metapopulation model capable of real time spatially refined surveillance and forecasting during emergencies." It doesn't help that the manuscript ends rather abruptly. This is a long manuscript and at times it helps to connect the part in the results to the part in the discussions, and this requires good organization of the paper.

***ANSWER:*** *We have made major modifications to the Discussion section as mentioned above under the comment on a lack of objectives. In particular, we have reorganised the text to bring together related parts of the discussion. We have also removed some less central issues, added supporting text, and a short conclusion summarising the paper:*
*"In conclusion, regional differences are a key trait of the COVID-19 pandemic. We propose a stochastic regional metapopulation model for situational awareness including real-time mobility data, calibrated to combined daily counts of cases and admissions using a novel Split-ABC-SMC technique. We provide estimates of county-specific and national reproduction numbers for Norway, and three-weekly projections of regional hospital and ICU beds, thereby documenting our modelling pipeline supporting the Norwegian health authorities and government since the early phase of the COVID-19 pandemic. Our approach is rather complex, focussing on geographical aspects at the expense of adopting a simple transmission model within each unit. We show that the model provides better projections compared to selected reference models. However, other studies, including adaptations to other countries, and additional data, are warranted to validate the approach further".*

Besides these major comments, I have some specific comments about the manuscript listed below:
I know that you're using mm/dd/yyyy, but I would recommend that dates be expressed in a less ambiguous way, e.g. Jan 1, 2020 or 2020 Jan 1.

***ANSWER:*** *The date format has been changed throughout the manuscript to e.g. January 1, 2020 as suggested, and we have specified the date format in Table 2 to avoid ambiguity.*

Line 34: lowest *COVID-19* death rate

***ANSWER:*** *Thank you, we have corrected the sentence as suggested.*

Line 102: There are 356 municipalities in Norway but 11 counties are modelled. It helps to explain what is municipality and what is county, also do you need to introduce the concept of municipality here since you're going to aggregate them into 11x11 mobility matrices anyway? Note also that in the manuscript in general, county is also known as region sometimes, perhaps it's wise to stick to one term?

***ANSWER:*** *Though we agree that we do not need the concept of municipality as the model is on the county level, the mobility data are aggregated from municipality level. Hence, to be precise in exactly what we have done, we have chosen to describe the processing of the mobility data on the municipality level. In the manuscript, we have decided to use "region" when discussing the general model and methodology, and "county" when we are in the specific case of Norway. The counties are specified in the map in Fig 1, and the population sizes and densities of the counties are provided in Table 1. We have added the following sentences to the Case data section to clarify this further: "The counties are further divided into 356 municipalities, but in the case of Norway we have chosen to simulate on the county level. In the paper, we will use the term region when we refer to the general model and methodology, and county when we refer to the case of Norway".*

Line 129: What's "churning" here?

***ANSWER:*** *The term churning in this setting refers to customers that change mobile network provider through changing their SIM-cards. Changing the SIM-card will result in the customer "disappearing" from the network in case of churning out, or "appearing" in the network in case of churning in. In Norway the total churn activity between mobile network providers is relatively low with an approximate monthly churn rate just short of 1%. Hence, there are very small corrections that are done due to churn activity of the customers.*

Line 226: example for "changes in behaviour or viral properties"?

***ANSWER:*** *We have specified the sentence by changing it to: "In addition, we included further changepoints to capture other potential gradual changes in behaviour or viral properties like the gradual takeover by the more transmissible alpha variant in winter 2021 and potential tiring of compliance with restrictions over time".*

Pg 12 and 13: I appreciate that you go into the details of how each of these data is extracted and analyzed or calculated, but would it help to be brief here and put the details in the SI? It helps to reduce the length and focus on your objectives.

***ANSWER:*** *We have moved most of pages 12 and 13 to the supplement as suggested, and replaced it by the following subsection Model for admission to hospital and intensive care:*
*"Based on the estimated incidence in each region obtained from the spatial metapopulation model, we modelled the number of individuals admitted to the hospital. In the model assuming a common, national transmissibility, we used a binomial distribution with hospitalisation probabilities provided in S1 Text Table S2. In the model with region-specific transmissibilities, we instead used a 28-days moving average of the daily estimated hospitalisation risk. We use a moving average because the daily hospital admission data were prone to randomness and noise. This means that in the model with a national transmissibility, we assumed hospitalisation risks constant every calendar month, while when we modelled transmissibility regionally, we assumed smoothed daily hospitalisation risks. It would be straightforward to also include daily moving averages in the national hospitalisation model.*
*When simulating, once an individual was selected to be sent to hospital, we generated the delay from onset of symptoms to hospitalisation and the length of stay in hospital from two negative binomial distributions. For Norway, we estimated these parameters from individual-level registry data.*
*The hospitalisation risks were based on the age-specific estimated hospitalisation probabilities in Salje et al. 2020. Even though the compartmental model did not have age compartments, we needed to take into account age when computing hospitalisations, as the probability of requiring hospital care was highly dependent on age. We corrected the hospital admission probabilities to account for age-dependencies in transmission, by adjusting for the age distribution of the positive cases. For the period where we do not use the test data (prior to May 1, 2020), we computed the probability of hospitalisation by taking into account the demographic age profile in each region. The details on the delay distributions and hospitalisation risks are provided in S1 Text Section S1.2".*

Formula about Line 305: What is this quantity? What is t in this quantity?

***ANSWER:*** *We have specified that t denotes day and have clarified that we multiply by the formula. The formula models the logistic takeover of the alpha variant where t is the number of days from January 1st 2021. As mentioned in the text, we multiply the resulting factor by our daily hospitalisation risks so that we can approximately reproduce the proportion of cases of the alpha*

*variant from January until March 2021 and how this led to a gradual increase in severity from 100% (factor=1 before January 1st) to 160% (factor 1.6 approx. from March 2021). The section is now moved to the SI Text, Section 1.2.2*
Line 306: Assuming t means number of days since 2021 Jan 1. 9% increase occurs at around Jan 21 and 50% increase occurs at around Feb 12. These days are neither the center nor the end of the month, how did you choose these numbers to report?

***ANSWER:*** *The reference values correspond to monthly estimates, so in order to compare them against daily data, we considered them to be the mean over each month. The mean factor obtained for January with this formula is 1.09, and 1.50 for February.*

Formula at Line 317: Nitpicky here but pi_0 and pi_1 would have meant detection probability at day 0 and day 1; I don't this is what you want?

***ANSWER:*** *Thank you for pointing this out. We agree about the unfortunate notation and we have changed the detection probability to \pi(t) instead of \pi_t.*

Line 355 to 358: So you're comparing **final** observed hospital incidence and test to simulated ones? If so, it seems possible for the model to be completely wrong in the middle of trajectory but still get it right finally (e.g. wildly low incidence in the beginning and wildly high incidence in the end). Or did you compare monthly?

***ANSWER:*** *We compare daily and regionally, i.e. a sum of the deviance in each region on each day. We clarify the text in the Calibration section further, as: "We used the least squares between the simulated regional daily hospital incidence and the observed regional daily hospital incidence, summed over all the regions".*

Line 456 to 457: Not sure what this sentence means, presumably you meant that 90% of hospitalisation occurs within 15 days of infection?

***ANSWER:*** *We refer to the simulated hospitalisations (as we know their delay distribution). When we want to compare simulated and observed hospitalisations on day t, we cannot start the simulations on day t, as most of the hospitalisations on day t correspond to simulated transmission events that occurred days ago. We have tried to clarify further, and the text in the Split-SMC-ABC section now reads: "As the simulated hospitalisations and laboratory-confirmed cases on t_{cal} corresponded to simulated transmission events that occurred a certain number of days before, it was necessary to start the simulations a certain number of days before t_{cal}. We chose to start the simulations at t_{cal}-15, as 15 days covered approximately 90% of the expected hospitalisations in the simulations in our setting (which are more delayed than the positive tests) ".*

Line 523: 77% and 88% of pre-lockdown value?

***ANSWER:*** *Correct, we have added the specification.*

Fig 3, 4: Explain the different shade of blues.

***ANSWER:*** *Thank you, we have added the band definitions to Figure 3 and Figure 4.*

Line 534: Did you mean that you were using this model to publicly project case numbers, and people read your prediction and change their behaviour? If so, this is not conveyed the paper at all (that your predictions are perhaps being used by the government or media to communicate with the

public.) Table 1: "Bottom: Estimated reduction of transmissibility ... " as in reduced by percentage? or absolute reduction?

***ANSWER:*** *Thanks, we have added a clarification of the reduction in the table. We have also changed the sentence in the Regional predictions section to: "However, as the predictions were used to inform decision-making by the Norwegian government, publicly available and communicated by the media, they often triggered new policies and behavioural changes, which in turn influenced reproduction numbers".*

Line 548: What is appropriately coherent?

***ANSWER:*** *Thanks, we have removed "appropriately".*

*Table 1 and 2, Section in Line 570 onwards: What should I learn about this that is in line with your objectives?*

***ANSWER:*** *We have clarified our objectives as described above. These include an estimation of regional reproduction numbers. For comparison we also estimate national reproduction numbers. Both are central indicators in our ongoing situational awareness and forecasting weekly reports. We chose to highlight the early lockdown period for the regional estimates as this period is well studied in other countries, adding to the literature Norwegian estimates.*

Fig 5: All the other lines except for the green one is practically unreadable. Is there a need to code counties in numbers? Also, once again, is this presentation in line with the objectives?

***ANSWER:*** *To streamline the paper, this section is now moved to the SI Text. Figure 5, now Figure S35, has been improved so that all counties are visible.*

Line 582 to 587: This section comes out of nowhere and is unclear which part is it tied to... is this related to estimates of pi_0 and pi_1?

***ANSWER:*** *We believe that this is an interesting number, and we therefore report it. It is calculated as part of the calibration process, as specified in the Section "Importation of the virus". It is necessary for the model of the imported cases, as the estimated reproduction numbers will be wrong if the import of cases is wrong. We have taken away the subsection in the Results section, the results are now presented in a shortened version in the subsection "The national picture".*

Line 593 to 594: Why these five dates? And why are they not continuous?

***ANSWER:*** *The dates are not continuous as each calibration is very computationally time-consuming, and hence we had to make a choice. We wanted to include both dates where we knew that we would perform badly (when there are recent changes that are not visible in the data) and dates where the situation is more stable, like October and September 2020. We included multiple dates to validate the results. We have added information about the choice of dates. The sentence now reads: "We predicted the weekly hospitalisation incidence and the number of new confirmed symptomatic cases region-wise for three weeks ahead following April 1, 2021, March 1 2021, November 1 2020, October 1, 2020 and September 1, 2020. As the calibration is computationally time consuming, we restricted the comparison to five periods, selected to cover both relatively stable and changeable parts of the epidemic".*

Line 595 to 596: Might want to explain briefly what are energy score and CRPS? What number is considered good in general? Or is their sole purpose only to compare two models?

***ANSWER:*** *The proper scoring rules are only used to compare predictions from different models and they do not give an overall assessment of the predictive performance. We have added a sentence in this paragraph to clarify this, namely:*
*"Lower scores indicated better predictive performance. Scores of predictions from different models can be compared to conclude which model is best. However, the scoring rules do not provide an interpretable quantification of how well the predictions performed in absolute terms".*

Line 642 to 644: Is this a "suggestion" or you did that in this work?

***ANSWER:*** *Thanks, we have rephrased it to "have suggested".*

Line 645 to 656: Vaccination is almost not relevant in this work, and you did not mention it at all previously. Is this a "future direction"?

***ANSWER:*** *Yes, it is something that we are currently considering.*
*In the model we do not consider vaccination explicitly. This was warranted for the period under study given the roll-out of the first dose and the circulating variants at that time. However, the continuation of the pandemic over several years with novel lineages, multiple vaccination doses offered, waning immunity, reinfections etc. makes the current situation highly complex. This applies to models, which aims to follow in detail the implementation of vaccination using registry data, and models like the present that aims to estimate the effective reproduction numbers. In both cases, the risk of hospitalization will depend in time on the level of immunity in the population and the protection against infection and severe infection against the dominating strain at different times.*

Line 657 to 660: How is it straightforward?

***ANSWER:*** *We agree that the phrasing is not good and have rephrased it to indicate potential extensions, emphasising that this, of course, requires additional assumptions: "However, extending the model framework to include partial protection or waning immunity is relatively straightforward to implement by for example moving individuals from the recovered compartment back to the susceptible compartment, but it of course requires additional assumptions".*

Line 729: Very helpful to mention this in your method (the SEIR model part for example) that one time step in your model is 6 hours. Confusing given that incidence and test data are in daily resolution, some of the formula seems to be in daily time steps too?

***ANSWER:*** *We have added the definition of the time step which was missing. We also added the following specification: "Note that the disease spread model has a time resolution of six hours, while all the surveillance data have a daily resolution. We therefore only used every fourth time step of the model when comparing to data, corresponding to 24-hours between each simulated value".*

Line 746 & 749: Did you "show that" in the manuscript that prioritising visitors before host ... gives similar spatiotemporal dynamics? Did you "document that" your regularisation method retain the real characteristics? Or is this based on preliminary work? Not asking you to show/document them here just a poor choice of wording.

***ANSWER:*** We have clarified this wording by mentioning that we obtain similar spatiotemporal dynamics measured by the timing and peaks of the infections. We have also clarified what we mean by the text regarding regularisation. We want to say that with the mobility rule adopted, prioritising in ascending order visitors, hosts, others, we obtain similar spatiotemporal dynamics when utilising the original (“true”) mobility matrices and the regularised mobility matrices.

**Reviewer #4:**: Carl A. B. Pearson

OVERVIEW

In this work, the authors report on incorporating high resolution mobility data into a mechanistic model of SARS-CoV-2 dynamics to inform near-real-time short-term projections of regional transmission and hospitalization data in Norway.

Overall, I think this work is an ambitious integration of data into a mechanistic framework, introducing a potentially interesting fitting approach, which is suited to the aims and audience of PLOS Comp Bio. However, I have several reservations about the work.

I'm roughly categorizing my concerns into what I'll summarily label mathematical (concerning the theoretical validity of the inference / calibration algorithms), engineering / operational (practicability / utility / suitability of the approach for purpose), and scientific (concerning data & potential for replicability and/or analysis using other theories). I realize those aren't the precise distinctions everyone would make here, just trying to give them a rough shorthand.

MATHEMATICAL CONCERNS

I find the Split ABC-SMC idea intriguing and it seems intuitively plausible that it could work as an inference technique. Here I'm defining "work" to mean asymptotically identifying best estimates, having intervals mean what their labels state, etc - the features that are demonstrated theoretically for various other ABC algorithms, but are not *necessarily* features of an arbitrary new tweak to using ABC. However, the manuscript provides neither theoretical or practical demonstration that this approach does those things. Personally, I'd prefer the practical version: using known model parameters, create a synthetic version of the kind of data you perform inference with, and characterize the performance of the method in recovering the known parameters.

I'd find that kind of demonstration sufficient, but in general the manuscript needs more evidence about how this method performs in abstract when the "answer" is known.

***ANSWER:*** *Thank you for the suggestion. We agree that such a practical demonstration would benefit the manuscript and we have therefore included a simulation example to illustrate the performance of the method. We have generated a simulated data set with known, underlying parameters, and then ran the calibration method on that simulated data set. Note that since the disease spread model is stochastic, and we are only calibrating to one realisation of the underlying stochastic process, it might be that the best fitting parameters to the simulated trajectory are not exactly the true values used to generate the data. As the calibration is so time consuming, we have still chosen to only calibrate to one such trajectory, though ideally one should repeat it many times. Most of the true parameters were recovered in the illustrative example, which is added in the SI Text, Section S2.10.*

Related to this issue: a few places in the manuscript refer to picking between ABC targets, noting that hospitalization only resulted in higher variance. Seems like variance practically translates to uncertainty in forecasts, which isn't necessarily inappropriate. If the authors think that additional uncertainty is inappropriate, they could argue that from e.g. coverage. They have calculated those values, as indicated in the supplement - though the performance of the forecasting method should defiitely be main text results. A substantial part of what's being reported in this work is the performance of the approach, not just the raw outcome predicitons it makes.

***ANSWER:*** *The coverage is also included in the main paper as part of the figure depicting performance scores, not only in the supplement. Though we agree that it may be that additional uncertainty in the predictions would be appropriate, we do not think that this should be obtained by excluding information/data sources. Hence, we think that to obtain estimates of the reproduction numbers, all the information available should be used (here both test data and hospitalisation data). Regarding coverage, we obtained too high coverage when using only hospital data for one-week ahead predictions (coverage 100%), and too low coverage when using both data sources (0.8-0.85 coverage when of 95% intervals). However, we only checked this for forecasts from one date so it is difficult to make a strong statement about the effect.*

ENGINEERING CONCERNS

My read of the authors argument is that they are advocating for an approach to making short-term projections of rates of transmission generally and hospitalization specifically that will be useful for policy decision-making (e.g. the public health outcomes of policies on the spectrum of relaxing => maintaining => enhancing intervention activities) and/or logistical planning for health care capacity. I am using "approach" here to encompass roughly 1) what data to collect (regionally dis-aggregated, mobility + epidemiological count data, but also the assorted other "standard" epidemiological measures [like dwell times in conditions, conditional probability of outcomes] & national statistics like age distribution), 2) how to synthesize that data (i.e. with region-level-resolution compartment model, with interacting regions), calibrate it (split SMC-ABC procedure + hospitalization/test positive count metrics), and what to predict / how to evaluate that prediction.

Implicit in that advocacy is that readers should use this method (or something like it) and / or the authors were justified in using this method to provide estimates for decision-making. To operationalize that argument, people need to compare this approach to alternatives for producing the same outcomes from the same data (or alternative data with some sort of valuation comparing collection requirements). What immediately comes to mind for the authors stated target (short-term, regional hospitalization counts) would be something like the various options for Rt estimation

and forecasting / projection of cases / hospitalizations / etc (e.g. EpiNow2 R package, ). At the very least, some sort of null model with zeroth (static rate of outcomes) or first order elements (static rate of change in rate of outcomes) should be used. This requirement is one of the (many) standards proposed for forecasting / projection papers summarized in EPIFORGE (https://midasnetwork.us/epiforge-2020-guidelines/), which reflects at least some consensus in the community.

***ANSWER:*** *The main purpose of comparing the predictive performance of the national and regional model in the paper is to show that using a regional model gives better performance. From this perspective we view the national model as a "null model". Our argument is that our regional model gives both a better description of the epidemic in Norway, in terms of county-specific reproduction numbers, and gives better predictive performance than a simple national model. While this does not necessarily justify all the details in the model, it gives an indication that, overall, the fairly complex model gives better predictions than just using a national model.*

*In addition to the downsampling from the national model, a natural comparison would be running a simple null model in each county. We have therefore included a simple zeroth-order model in the Results section that is described it in the SI Text Section S1.4 to show that the regional model still gives better predictive performance. A first-order model is not trivial to implement due to the very few hospitalisations in many of the periods we consider. Therefore, it is very difficult to estimate any trends based on simple regression techniques. We have updated the validation results together with some of the text.*

I don't see in here a clear statement of how much additional predictive power is wrung from the additional complexity baked into this mechanistic model *for the target predictions*. I agree that such mechanistic complexity may be necessary for other kinds of predictions (e.g. projections about total incidence over a longer planning horizon) or for critical-but-rare edge cases (e.g. what will happen initially with introduction of a new variant). But I don't see sufficient argument here that this model complexity is justifiably better than alternatives.

***ANSWER:*** *We provide evidence of improved predictive performance going from a national model to a regional model. Furthermore, we have now also included a regional baseline model. While we agree with the reviewer that this does not show that all the added complexity is needed to get good predictions, good predictive performance is not the only goal of using this model. We also aim to estimate regional reproduction numbers as accurately as possible. These numbers have been useful for public health authorities during the COVID-19 response.*
*There have been multiple times during the pandemic where inclusion of mobility in the model lead to different estimates of the reproduction numbers; this justifies inclusion of this complexity in the model even if it will not necessarily lead to improved predictive performance. We have included a short description in the SI Text Section S2.8 that shows the significant effect of mobility on exponential growth rates in one large county, Oslo. During the pandemic, fairly large differences in the incidence levels between countries have been a persistent feature of the epidemiological situation in Norway.*

While the manuscript describes several other latent parameters or estimates that come out of the model, the discussion doesn't generally take a position on the value of thinking of these as predictions with direct public health consequences or of scientific interest (i.e. to be validated by new measurements).

**ANSWER:** We believe that both aspects are important. The comment is not explicit about which parameters or estimates that is referred to. In relation to the reproduction numbers, they have in fact been used to inform policies in real-time in Norway. We believe that the numbers are also of scientific interest to be validated by new measurements. While these could in principle be verified by using other data sources or other models, this is difficult in practise. We have used all the available data in the model. Another parameter, which has been used by state epidemiologists is the detection probability, and where we reference alternative estimates in the Discussion.

Aside: I also think that more complex models invite lots of criticism of specific subelements. It's tough to address the validity of those criticisms without detailed sensitivity work. For example, some elements may seem very different from what "actually" happens, but the lower detail may not matter or may be counterbalancing assumptions elsewhere in the model (made for practical reasons). In the last section, there will certainly be a few of those, but as I said - it's unclear if they're important or not.

SCIENTIFIC CONCERNS

The distinguishing feature of science from math is the wide, transparent publication of input & result data; both share a requirement to describe a model to translate from inputs to outputs.

In that sense, the key data elements here seem to be the mobility measurements, the case series, and hospitalization series. Readers will understand that the raw data cannot be indiscriminately shared given privacy implications. However, as a practical matter, I doubt the raw data can be realistically requested for anything short of a full blown replication, and (unfortunately) about the only way that sort of activity would have any professional value would be some sort of systematic replication attempt of many modelling results. I suspect by the time such could be planned and funded, the pertinent raw data sources here would have decayed to an unusable state (n.b. I'd bet this is also true of any other publications considered for such an enterprise).

Given that practical reality, I think the authors need to do more to make this work meet data availability standards for something to constitute scientific activity. I am going to suggest a rough solution I think would satisfy the epistemic requirements, but of course there may be others and I'm open to other ways of addressing this issue.

My read of the model itself is that it compares to a time series of case and hospitalization data that is sufficiently aggegrated (no age, sex; at the regional level rather than high resolution) to confer anonymization. That aggregated data set could be made directly available as part one of the repositories, along with the aggregation scripts to go from the raw format, in plain text (csv) format. It won't be a particularly large file, even in plain text - order thousand rows, order 10 columns.

Related, I should point out that it's not obvious that hospitalization data is even available because of the 1K files display issue in github - there are serious organizational issues with that repository (e.g. files could be subdirectoried into xls/EVENT/date.xls and csv/EVENT/date.csv to make it much more manageable, though I understand its structure is now a dependency in various analyses) - and I don't

think it's appropriate to expect a reviewer (or reader) to jump through hoops to check that. The recommended solution (clone the repo) seems likely to be inappropriate for bandwidth, data limited LMIC settings.

I think the same character of recommendations should be reasonable for the aggregated, regularized mobility matrices. The manuscript describes dropping low count movements, and has aggregated them to lower resolution in time and space than they are in the raw data. If the "example" mobility data in the availability statement is in fact already what I'm suggesting, then it should be characterized as such.

Again: I am open to alternative solutions by the authors. There are probably constraints on what they can do with the data that haven't been made explicit in the manuscript, some of which may preclude what I've suggested (aside: the authors / journal may wish these constraints be made explicit in the data availability statement to calibrate expectations when contacting the teleco). That understood, I have tried to paint a clear target of what I think is appropriate data to work from in terms of following the tenets of scientific activity.

***ANSWER:*** *We agree with the reviewer that transparency and data sharing is very important. However, we are working under the regulation of data sharing defined by the Norwegian law and administrated by the Norwegian registries. We have added additional information about where available data can be obtained. However, for some of our data sources, only weekly data has been made public. The mobility data is owned by Telenor Norway and shared with the Norwegian Institute of Public Health on a contractual basis. Legal and privacy issues is a major contemporary issue, but we still believe that a modelling study, such as ours, has merit and contributes to the scientific discussion about proper methodologies and approaches.*
*We have added further information about data availability to the manuscript.*

Distinct from dealing with the issue of providing appropriate data for study replication, I think there are methodological gaps in the description of getting from raw data to the aggregated input. For example, while I definitely wish more countries would use a unique personal identifier for this kind of data and applaud Norwegian authorities for attempting to do so, I doubt that collection went perfectly - so how are data issues identified and resolved?

My personal solution to this would be to a high level plain language description in the main text, a more detailed description mixing plain language, math formulae, and algorithmic pseudo code in the SI, and the actual cleaning / transforming code in a repository. As written, the manuscript neglects some key details (namely, the general scheme for data hygiene), but also feels a bit too verbose in the main text - much of what's there currently would be much more accessible as specific bite-sized chunks in SI sections. The code for cleaning / formatting / etc is not obviously available.

**ANSWER**: *We were using data from the Norwegian preparedness registry Beredt C19, merging data from various registries and other sources using a unique Norwegian social security numbers. The registry is responsible for conducting internal quality checks. We have not been involved in the data curation or quality studies. In general, however, the number of errors reported are very small.*

ASSORTED MINOR ITEMS:

- How might "regional" be portably defined? This analysis seems to define it in terms of admin 0 (nation) vs admin 1 (immediate subnational) - but for other settings (e.g. US and US states, some of which exceed Norway's national population) that division is very different from the one in this analysis.

***ANSWER:*** *We have added a comment on the spatial scale in the Discussion section of the paper. If a region is too large, it might not be reasonable to assume homogeneous mixing and a common transmissibility parameter within the whole region. If a region is too small, there might not be enough cases to obtain useful estimates of the transmissibility parameters. Moreover, the computational time scales quadratically in the number of regions, and hence the spatial scale is a trade-off between accuracy/detail and computational time. The model is advantageous when working with regions which are heterogeneous in transmissibility, but still connected through the mobility network. The added paragraph summarises this as follows: "In the case of Norway, we chose a county-level description in the regional model. The choice of spatial scale is a trade-off between accuracy/detail and computational time. The computational time for the infectious disease model scales quadratically in the number of regions, and hence increasing the number of regions is costly. In addition, if the population size in a region is too small, there might be too little signal in the data to obtain useful estimates. If the region is too large, it might be unreasonable to assume homogeneity within the region".*

- I'm unclear on the exclusion of certain negative tests? i understand it for certain analyses - it's like you're trying to consolidate "negative" episodes, but ambiguous how to translate "duplicate" negative tests from an individual testing episode into insights about unobserved prevalence / incidence.

***ANSWER:*** *Each individual was registered with at most one test in each 7-day window. If an individual had more than one test during a 7-day period, then: 1) if only one of the tests was positive, this one was registered; 2) if more than one was positive, the first of the positive tests was registered. One person was only registered with one positive test in a six-month period ; 3) a negative test result was not registered if it was less than 7 days since the last negative test. This was the definition adopted in the data registry.*

- re mobility assumptions - why these? what alternatives were explored? do those alternatives make a difference in mobility estimates? some indications in discussion, but not much by way of how much they matter. Re the bit about home establishment, why not based on modal location or modal location at night over a long period? How do inferred populations based on mobile data home establishment compare to other data sources for relative populations?

***ANSWER:*** *As the mobility data does not allow to follow individuals over time, we need to make assumptions regarding the residency of the population segments to be move in each time interval. We explored several alternative choices, which are compared and discussed in the SI Text Section S2.7. Overall, using the metrics of timing and size of the epidemic, we did not find consistent and significant differences between the various assumptions. Most of the mobility is associated with commuting, but we do not have comparable data from other sources available to make a comparison with.*

- the "four" mobility matrices (121 on pg 6) - first mention? what four?

***ANSWER:*** *Thank you for the observation, this was not clear. We have added the following specification: "First, the four mobility matrices for the given weekday were averaged, i.e. the four six-hourly matrices for each 24 hours interval"*

- Re mobility regularization: is population conservation really appropriate? I understand the practical value of it, and would be okay with conserving total population, but presumably, Norway has some seasonal shift in typical locations of individuals? Seems inappropriate to enforce detail balance at each time step.

***ANSWER:*** *This is true, and as stated in the paper, we use the actual data when simulating the past. The problem is in the future predictions, when we use the regularisation. This is because we chose to repeat the most recent mobility pattern each day of the future. If there is an imbalance in in- and outgoing flow in that matrix, then this procedure can in worst case result in empty regions (and it will if you continue to simulate for a long enough time period). Whether this is a problem or not will depend on the population sizes of the region, the size of imbalance in mobility, and the number of days simulated into the future.*

- Regarding preferential returns of vistors to their home location: how does that comport with the disaggregated mobility data indications?

**ANSWER:** *We do not understand the question. To be clear, we do not have access to disaggregated mobility data.*

- Does SEIR status change movement behavior? S and R shouldn't presumably. Probably E also fine. But I? Undetected Is maybe, but presumably Norway had isolation rules for detected Is.

***ANSWER:*** *Yes, one could explore different rules for mobility for different classes. However, as we do not know the infection status of the travellers, we have chosen not to do this, as it would require ad hoc assumptions. We have added the following specification: "We moved individuals independently of their disease status. Another option would have been to decrease the probability of travel for e.g. the infectious symptomatic, however we have chosen not to do this, as it would require ad hoc assumptions on the size of the decreased travel probability".*

- for seeding, wording suggests importation of known metadata (i.e. infections designated as travel acquired). Is that available from the reference epidemic data?

***ANSWER:*** *Yes, in the registry, infections are classified as imported or local, filled in by health care providers during patient interview based on information on likely transmission and travel history. However, the information is subject to uncertainty.*

- using smoothed average instead of actual values - so what if the epi data are noisy? Doesn't the fitting algorithm / distance function / sequential quantiling address that?

***ANSWER:*** *Yes, the model should handle variability/noise in the number of individuals infected on each day. However, it is more ambitious to properly model the reported number of cases each day. For example, there seems to be different reporting on weekends and holidays, and Monday seems to differ from the rest of the weekdays. This could be handled in an observation model which takes*

*weekday into account and learns from historical data. However, we chose to simplify the problem by averaging instead.*

- The lack of age structure muddies insight from other work on that - the aggregate estimates of severity fraction with time leaves a lot of wiggle room in the model, which might be inadvertantly counterbalancing some estimate that the model makes.

***ANSWER:*** *We are not sure we understand what is meant in the question. We extract information on the age profile of the prevalence from the test data in order to get a more accurate hospitalisation probability given infection (therefore, the risks are calculated from data and are not estimated by the model in any way). We only take into account the age groups when we estimate the aggregated hospitalisation probability which, in turn, is used to map the total incidence from the model into hospitalisations. We are well aware of the limitations of this approach in the sense that, without age structure in the epidemic model, we do not aim to explain the differences in terms of the age profile of the prevalence. We also do not provide age-specific predictions in the paper.*

- tables 1-2:
* the concrete numbers suggest a certainty that seems unwarranted
* this would be better as a visualization, particularly for highlighting how long the estimation periods where relative to each other (and relative to estimating a single R value for the period)
***ANSWER:*** *We have added a figure with the national reproduction numbers in the SI Text Section S2.1 We have chosen to keep the tables, as they contain additional information and are easier to read off for comparisons with other countries etc. We have not included a figure of the regional reproduction numbers, as this would result in 11 figures, which we think would be too many.*

- For mobility metric comparisions: is it appropriate to compare reductions / changes relative to only prior periods of analysis? or should comparison be reductions relative to historical trend for those periods?

**ANSWER:** *This section has been moved to the SI Text Section S2.6 to streamline the manuscript. You are right that it would give additional insight to compare to historical trends for those periods. Unfortunately, the mobility data have not been stored before the pandemic, and hence such historical data do not exist. We have added the following sentence to the SI Text: "We compared mobility to March 1, 2020, as we do not have mobility data before the pandemic, which also means that we cannot compare general seasonality in mobility volume".*

- Discussion implies extending the model framework to include reinfections is straightforward. That seems overconfident. While the structural addition of waning to the equations is well-specified, there are several options to choose amongst, AND the "model framework" should be thought of as including changes to the data injestion & the fitting targets as well. While the authors might not change the fitting targets, that is still a choice that entails as strong as assumptions as any other.

***ANSWER:*** *We agree, and have changed the comment to: "However, extending the model framework to include partial protection or waning immunity is relatively straightforward to implement, for example by moving individuals from the recovered compartment back to the susceptible compartment or introducing additional vaccination compartments, but it of course requires additional assumptions".*

- Re split-ABC-SMC: I'm not sure I understand the bit about increased probability of accepting parameters from previously accepted trajectories? Is that an algorithmic choice, or just a reflection of how the posterior => prior for next step works?

***ANSWER:*** *This is an algorithmic choice, as it could be that certain past trajectories are more coherent with the future data (for example relating to proportion susceptible). We have added the clarification: We chose to do this as it could be that certain past trajectories were more likely, given the current data.*

- There's no little mention of deaths in here; I understand that the model isn't *for* that, but would be a useful independent validation of the transmission aspects.

***ANSWER:*** *Though we agree that death counts also contain information on the transmissibility and could be used for validation, it would require a good model for COVID-19 deaths in Norway, with assumptions about risk and delay. Moreover, as cause of death is often unknown/unclear and the number of deaths were few in Norway as the epidemic was highly controlled until the Omicron variant arrived, this data set can be very noisy. As we were not interested in death predictions, we consider making such a model for death counts as outside the scope of this work.*

- The novelty assertions seem either wrong or qualified in someway that I don't understand. There's other spatio-temporally explicit work, including using mobility data, that is making short-term forecasts of transmission / outcomes. I don't see the need to assert novelty / priority here - this is a potentially interesting entry in a difficult-to-get-right genre.

***ANSWER:*** *We agree with the reviewer that there are other spatio-temporal models that uses mobility data for forecasting. However, our use of 6-hourly national, real-time data since March 2020 and onwards with fine granularity is to our knowledge novel, and different from using historic mobility data. Notably, in Norway the epidemic was highly controlled until the arrival of the Omicron variant and many municipalities had recorded zero or few cases, making mobility particularly important. See also answer to Reviewer #1. We have added an explanation to the the Discussion section "While there are published SARS-CoV-2 models informed by historical mobility data, e.g. ref Gatto et al, using commuting data from 2011, to the best of our knowledge, our modelling approach is first to employ real-time national mobility data with fine geographical resolution".*

- Regarding fitting resource indications (one week, 1400 cores): what is run time for a "single" sample? how many samples does that total calibration time correspond to?

***ANSWER:*** *The run-time for a single sample is in seconds, hence what mainly takes time is to find parameters which improve the error, not the disease spread simulation itself. We have added this information to the section Computational time and convergence in the SI text, Section S2.5.*

___________________________________

**Have the authors made all data and (if applicable) computational code underlying the findings in their manuscript fully available?**

The PLOS Data policy <https://journals.plos.org/ploscompbiol/s/materials-and-software-sharing> requires authors to make all data and code underlying the findings described in their

manuscript fully available without restriction, with rare exception (please refer to the Data Availability Statement in the manuscript PDF file). The data and code should be provided as part of the manuscript or its supporting information, or deposited to a public repository. For example, in addition to summary statistics, the data points behind means, medians and variance measures should be available. If there are restrictions on publicly sharing data or code —e.g. participant privacy or use of data from a third party—those must be specified.

Reviewer #1: Yes

Reviewer #2: No: See point 6 in the review. I can see that the code has been made freely available, as have the mobility matrices, but I can't see any of the count (cases or hospitalisations) in there, nor any discussion of how available they are. One suspects they may be official statistics and available (at best) only be request, but hopefully the authors will deal with it as part of the response.

Reviewer #3: Yes

Reviewer #4: No: The authors have made most of the relevant bits available; how to obtain the data or synthetic example data is obvious, as is where to get the disease model, but it's non-obvious where to find their fitting code. That appears to be in what the data availability statement refers to as example mobility data.

It is not obvious where the overall analysis flow appears. Again, this appears to be in the repo referred to by the data availability.

In terms of unambiguous citations, the authors should produce a release tag for the repositories or at least reference a specific commit. I also recommend that they view the repositories (that they control) as effectively supplementary materials submissions, and thus do a bit more organization in the top level READMEs to walk readers through what's where, how to use the repo, etc.

As noted in more general notes to authors, I do think there should be a different set of data available.

***ANSWER****: We have now provided a further repository with code used for fitting the Split-ABC model and some additional code. This repository includes a readme file specifically for the paper. The other repositories that are referred to is beyond our control and we can therefore not edit them.*

Data Requirements:

Please note that, as a condition of publication, PLOS' data policy requires that you make available all data used to draw the conclusions outlined in your manuscript. Data must be deposited in an appropriate repository, included within the body of the manuscript, or uploaded as supporting information. This includes all numerical values that were used to generate graphs, histograms etc.. For an example in PLOS Biology see here: http://www.plosbiology.org/article/info%3Adoi%2F10.1371%2Fjournal.pbio.1001908#s5.

Reproducibility:

To enhance the reproducibility of your results, we recommend that you deposit your laboratory protocols in protocols.io, where a protocol can be assigned its own identifier (DOI) such that it can be cited independently in the future. Additionally, PLOS ONE offers an option to publish peer-reviewed clinical study protocols. Read more information on sharing protocols at [https://plos.org/protocols?utm_medium=editorial-email&utm_source=authorletters&utm_campaign=protocols](https://plos.org/protocols?utm_medium=editorial-email&utm_source=authorletters&utm_campaign=protocols)
