# Supplementary material for: A real-time regional model for COVID-19: Probabilistic situational awareness and forecasting
Source: PLoS Comput Biol. 2023 Jan 23;19(1):e1010860. doi: 10.1371/journal.pcbi.1010860 (PMC9894546; doi:10.1371/journal.pcbi.1010860)
Supplement: Attachment — Submitted filename: Review comments.pdf [file pcbi.1010860.s003.pdf]

I appreciate the authors' attempts at addressing my concerns from the initial submission. In response to my initial points:

1. I am pretty sure I get how the hospitalisation risks work now. The detail in the supplement could do with some tidying, in particular, the expression of $h_{i,t}$ in equations S1-S3. If an index appears on the LHS of an equation, it should also appear on the RHS. Specifically, in S1, it is not clear how the RHS depends on time, at what time-scale does t_{ij} change? Do we have a new value every month/week/calibration period? The use of t for the proportion of tests is a bit confusing. In S2, B_{117} is a function of time and should be written as such. I am probably missing something obvious as to the significance of the 117. In S3, we have the reverse issue of S1, where now the index i does not appear on the RHS. The ratio of test variables: it is not clear how this differs from t_{ij} if at all. Could this simply be written as $h'_{i,t} = h_{i,t}B_{117}(t)$, where $h_{i,t}$?

2. I must admit empirical Bayesian approaches do leave me a bit cold, though I acknowledge the authors' comments on the prediction intervals. Going forward, rather than use the posterior estimates from the national modelling, could they use these posteriors as an importance distribution to generate a weighted sample from the prior rather than as a prior itself? This would solve the problem of initially finding good samples without invoking empirical Bayes.

3. Resolved.

4. I would still like it explicitly stated in the algorithm what should be done if the tolerance limits are exceeded.

5. See point 1.

6. Resolved

Minor Resolved

Overall, I appreciate the efforts that have been made to improve the model description in the text. The downside of having done this is that an already-long manuscript is now even longer. It would really benefit from being more succinct in parts and the authors should consider where they can move some text to the supplement. Some examples:

1. Page 17, line 392. The sentence that starts here has already been described in lines 386-8. Would suggest keeping only one.

Other minor issues

1. Abstract: "estimated to 85%" should be "estimated to be.."
2. Page 10: the use of $X_k(t), k = 1, \ldots, 6$ for the flows between states. Having just used X on the previous page to denote mobility, maybe these could be Y's instead? In the distribution of X_3 there is a δt that I don't think should be there.

3. Page 11: how are the X_{ij}^{t} distributed among disease states? Presumably everyone is equally likely to move irrespective of infection status, but as the model is over a discrete support, do these have to be taken as random variables based on the proportions in each disease state?
4. Page 15: Based on the number of parameters, it appears there are fewer changepoints in the regional model than in the national one. Is this so?
5. Page 17, line 378: The observed number and simulated number of positive tests: are these also smoothed over 7 days as well as k_t.
6. Page 20, line 455: It appears that you get some information on the past trajectories based on the current one, whereas on the previous pages they're described as being constant. I think what is meant is that, in effect, you are imposing a prior on the past trajectories and parameters that is defined only on the support from the previous analysis. Is this fair?
7. Page 22, line 499: Do you mean Section S2.1?
8. How does the national picture when aggregated from the regional models differ from when modelled separately?
9. Page 27, line 575: word missing "allowed us to".
10. Page 27, line 578: matter of taste, I would rather see "preferred" rather than "best".
11. Page 30, line 644: word missing "suffer **from**".
12. Page 30, line 656: See point 6 above.
13. Page 31, line 676: word missing "enter **the** model"
14. Page S2, Plural of data, "test data **are**…".
15. Page S15, I don't see how VHO2 is different to VHO.
