# Supplementary material for: A real-time regional model for COVID-19: Probabilistic situational awareness and forecasting
Source: PLoS Comput Biol. 2023 Jan 23;19(1):e1010860. doi: 10.1371/journal.pcbi.1010860 (PMC9894546; doi:10.1371/journal.pcbi.1010860)
Supplement: Attachment — Submitted filename: reviewer_commentsPCB_19122022.docx [file pcbi.1010860.s004.docx]

Dear Editor,

We thank you for the positive feedback and opportunity to submit a second revision of the manuscript “A real-time model for COVID-19: probabilistic situational awareness and forecasting” to PLOS Computational Biology. We also thank the reviewers for their additional comments. Please find below a point-to-point response to all comments by the reviewers, with our responses marked in red. We attach two versions of the main paper, one with all changes marked in red text, and one clean version.

On behalf of all authors,

Birgitte Freiesleben de Blasio

Editor comments:

1.Some of the reviewers still have a few suggestions for further clarifications. Reviewer 4 also asks you to compare the Rt estimates from your approach to those from an existing R package (e.g. EpiNow2) for a few example regions, which should be reasonably straightforward to do.

We have now added such a comparison with EpiNow2 for two regions. See the comment to Reviewer 4 for details.

2.Regarding the data availability issue raised by Reviewer 4: I understand that there are instances in which data cannot be shared, but the science is still important. To address the reviewer's concern, I think it would be sufficient to more clearly state the steps that would need to be taken and permissions needed to access the data and/or provide simulated data that could be used to validate the findings of the study.

The updated data availability status reads:

*Individual requests for the regional COVID-19 case data should be made to Helsenorge.no; information is available at https://www.fhi.no/en/more/access-to-data/applying-for-access-to-data/. Nonsensitive Norwegian COVID-19 case data are available at: https://github.com/folkehelseinstituttet/surveillance_data. Requests for access to mobility data should be directed to Telenor Research by email: TelenorResearch@telenor.com. A simulated mobility data set can be found at https://github.com/geirstorvik/smc.covid. The infectious disease spread model is publicly available on GitHub in the R-package spread, and can be found at https://github.com/folkehelseinstituttet/spread.*

Reviewer #1: The authors have carefully responded to all of my comments and most of the limitations of the work have been now appropriately discussed. I think the manuscript is now suitable for publication.

Reviewer #2:

I appreciate the authors’ attempts at addressing my concerns from the initial submission. In

response to my initial points:

1. I am pretty sure I get how the hospitalisation risks work now. The detail in the

supplement could do with some tidying, in particular, the expression of ℎ_(i,t) in

equations S1-S3. If an index appears on the LHS of an equation, it should also appear

on the RHS. Specifically, in S1, it is not clear how the RHS depends on time, at what

time-scale does 𝑡_(ij) change? Do we have a new value every month/week/calibration

period? The use of 𝑡 for the proportion of tests is a bit confusing. In S2, 𝐵_117 is a

function of time and should be written as such. I am probably missing something

obvious as to the significance of the 117. In S3, we have the reverse issue of S1,

where now the index 𝑖 does not appear on the RHS. The ratio of test variables: it is

not clear how this differs from 𝑡_ij if at all. Could this simply be written as ℎ’_(i,t) = ℎ_(i,t)B_117(t), where ℎ_(i,t)?

We have clarified the notation now. We have added the time dependency for the B_117(t) and fixed the indices. We agree that the choice of *t*  as the fraction of positive tests was confusing given that a subscript *t* corresponds to time in the LHS of S1. Following the recommendation we changed this.

Regarding the time scale of the probabilities of hospitalisations: In the national model monthly estimates have been used as shown in Table S2 and the comment regarding the logistic function of B117 : *“in the model with national transmissibility, we assumed an increase of 9% in January, 50% in February and 60% from March 2021 corresponding to the monthly values of the logistic increase assumed regionally”.* In the regional model, due to the low number of cases in some of the regions, we decided to apply a hybrid method. In this case, the probabilities of hospitalisations were estimated on a daily basis, but with smoothing factors in time, as the age profiles were estimated daily based on a *moving window over the last 28 days.* And on space because, *the country-specific proportions were adjusted using national data in those regions when few tested positive in a given period* as stated in S1.2.1 *.*

2. I must admit empirical Bayesian approaches do leave me a bit cold, though I

acknowledge the authors’ comments on the prediction intervals. Going forward,

rather than use the posterior estimates from the national modelling, could they use

these posteriors as an importance distribution to generate a weighted sample from

the prior rather than as a prior itself? This would solve the problem of initially finding

good samples without invoking empirical Bayes.

We respectfully disagree. Rerunning all the calibrations with a different prior would take weeks of running on a supercomputer and we do not see this as sufficiently important, as we do not share the concern of empirical Bayes. These empirical Bayes priors are only used for the parameters which are not of particular interest in the regional estimation. We also believe that running with different priors would mainly affect the uncertainty (as the prior means are based on the national posterior means) and the coverage of the regional model is investigated in Fig 5 of the main paper.

3.Resolved.

4. I would still like it explicitly stated in the algorithm what should be done if the

tolerance limits are exceeded.

This information has now been explicitly added to the algorithm.

5. See point 1.

6. Resolved

Minor Resolved

Overall, I appreciate the efforts that have been made to improve the model description in

the text. The downside of having done this is that an already-long manuscript is now even

longer. It would really benefit from being more succinct in parts and the authors should

consider where they can move some text to the supplement. Some examples:

1. Page 17, line 392. The sentence that starts here has already been described in lines

386-8. Would suggest keeping only one.

We have rephrased the suggested text about the prior distributions. We have also moved a subsection of the methods chapter to the supplement (regularised mobility matrices). In addition, we have moved a sentence to the supplement, namely:

*By April 12, 2021, NIPaR included 4.248 hospitalised individuals, of whom 735 needed mechanical respirator treatment, while MSIS contained 103.461 positive test results.*

and removed the following sentence:

*By April 12, 2021, the data contained 3.426.045 negative test results. We used the negative test data to estimate the probability of detecting a positive case.*

However, apart from these changes, we do not wish to move large parts of the methods section to the supplement, as we regard this as one of the main contributions of the paper.

Other minor issues

1. Abstract: “estimated to 85%” should be “estimated to be..”

The text has been changed accordingly.

2. Page 10: the use of 𝑋_k(𝑡), 𝑘 = 1,… , 6 for the flows between states. Having just

used 𝑋 on the previous page to denote mobility, maybe these could be 𝑌’s instead?

In the distribution of 𝑋_3 there is a 𝛿𝑡 that I don’t think should be there.

Thank you, we have fixed the expression for X_3, and renamed to Y’s.

3. Page 11: how are the X_(i,j)^t distributed among disease states? Presumably everyone is

equally likely to move irrespective of infection status, but as the model is over a

discrete support, do these have to be taken as random variables based on the

proportions in each disease state?

Correct, this is a multinomial distribution with probabilities based on the proportions in each disease state. We have added that specification to the section *Regional transmission model*.

4. Page 15: Based on the number of parameters, it appears there are fewer

changepoints in the regional model than in the national one. Is this so?

This is correct. As this is a harder estimation problem, we add fewer changepoints in the periods without changes in interventions in the regional model. This difference has been specified in the text.

5. Page 17, line 378: The observed number and simulated number of positive tests: are

these also smoothed over 7 days as well as 𝑘_t.

Yes, we have rephrased the sentence to avoid misunderstandings.

6. Page 20, line 455: It appears that you get some information on the past trajectories

based on the current one, whereas on the previous pages they’re described as being

constant. I think what is meant is that, in effect, you are imposing a prior on the past

trajectories and parameters that is defined only on the support from the previous

analysis. Is this fair?

We agree that the wording was imprecise. We have changed the text to specify that we restrict the past parameters to their posterior samples, instead of claiming that they are fixed to the posterior distributions. However, this is not a prior distribution, as it changes between rounds.

7. Page 22, line 499: Do you mean Section S2.1?

No, we mean section S2.3. Section S2.1 contains national results.

8. How does the national picture when aggregated from the regional models differ

from when modelled separately?

We have not focussed on the national aggregated results from the regional model, as we believe that the main advantage with the regional model is to provide regional estimates. Moreover, we do not calibrate to the national average in the regional calibration (i.e. we only compute county-specific distances, and hence a model which overestimates the cases in all counties would come out as equally good as a model which overestimates in some counties and underestimates in others). However, we do include a comparison of the regional model on national level predictions to the national model predictions in Fig 5. As the regional estimates are very uncertain for regions with few cases, the uncertainty in the aggregated prediction from the regional model is similarly affected, and the confidence intervals are wider.

Comparing the reproduction numbers is more difficult, as it depends on the proportion infected in the different regions over time, which changes due to mobility. Moreover, there are fewer changepoints in the regional model than in the national model.

9. Page 27, line 575: word missing “allowed us to”.

Thank you, we have added “us”.

10. Page 27, line 578: matter of taste, I would rather see “preferred” rather than “best”.

Changed accordingly.

11. Page 30, line 644: word missing “suffer **from**”.

Thanks, fixed.

12. Page 30, line 656: See point 6 above.

13. Page 31, line 676: word missing “enter **the** model”

Thanks, fixed.

14. Page S2, Plural of data, “test data **are**…”.

Thanks, fixed.

15. Page S15, I don’t see how VHO2 is different to VHO.

It is not different, VHO2 is simply a repetition of VHO to assess the model variability. We have added the following sentence to the supplement:

*“Hence, VHO2 is a repetition of VHO to assess the variability between two runs with the same rule”.*

Reviewer 3: I am satisfied with the edits and the response to my comments. Good job! I have only one major concern regarding the time step update in the model, and a nitpicky comment here.

> ANSWER: We have clarified further the connection between the local transmissibility model and the mobility by explicitly defining N_i^t through the mobility matrices X_ij^t in the Local stochastic epidemiological model section.

In addition, we have added a specification that we move individuals regardless of their disease state. We chose to do this as we do not know the infection status of the individuals in the mobility data. We agree that it is reasonable to assume that symptomatic individuals might move less, in particular if the symptoms are severe. We have still chosen to move them randomly independent of disease status as one would otherwise have to make quite strict ad-hoc assumptions where we do not have any data. The added specification to the Regional transmission model section is: ”We moved individuals independently of their disease status. Another option would have been to decrease the probability of travel for e.g. the infectious symptomatic, however we have chosen not to do this, as it would require ad hoc assumptions on the size of reduction”.

I think it is fair to assume that people move among counties regardless of disease status. However, I still find the section regarding the time steps update unclear. The movement of people shouldn't have just affected the N_t^i (which affects only the contact rate). Wouldn't S^i(t), E^i(t), I^i(t) be affected by the inward and outward flux of people too? Let's say there are 10 symptomatic infectious person in location i at time t. In the next time step, let's say none of them recovered, and no new exposed person become symptomatic infectious locally then I^i(t+1) would have been 10 according to equation (8). However, 3 of them move to other counties, and only 1 move into the county from elsewhere, then I^i(t+1) should have been 8 instead of 10 and this has some implication in the infection next time step. The movement of people isn't reflected in the set of equations (5) to (9). Perhaps what you did was to first update the number of people in each SEIR compartment to account for movement among counties at the beginning of each time steps (or maybe at the end of each time step), then you use equation (5) to (9) to update the movement among compartments? It would be nice to describe this in a few lines, if you don't want to over complicate the equations?

Yes, the number of individuals in the different compartments change when we move individuals, so the equations are only valid between mobility steps and for the individuals currently present in a region (i.e. as specified *The per-compartment counts S^i, E^i_1, E^i_2, I^i, I^i_a, and R^i are the number of individuals in each compartment who are currently present in the region.*)

We have added the following sentence to further specify the dynamics:

*The number of infected individuals in a municipality thus changes both with the disease dynamics between each six-hour period, and due to mobility between locations every sixth hour.*

> ANSWER: We have specified the sentence by changing it to: “In addition, we included further changepoints to capture other potential gradual changes in behaviour or viral properties like the
gradual takeover by the more transmissible alpha variant in winter 2021 and potential tiring of compliance with restrictions over time”.

Perhaps 'compliance fatigue' is a more commonly seen phrasing vs 'tiring of compliance' here.

Thank you, we have changed the phrasing accordingly.

Reviewer 4: I think the authors' reply is responsive to my concerns. I'm not sure those concerns are fully resolved, however.

On validity:

Regarding demonstration of Split ABC-SMC, the main text should indicate that readers may refer to the SI for a demo calibration of the method. The method should be clearly caveated to the effect of that it hasn't undergone the same sort of validation that other ABC-SMC methods have, so shouldn't be assumed to have the guarantees of those methods.

As suggested, we have added the following sentences to the main text:

*A demonstration of the calibration performance in a simple setting is provided in the S1 Text S2.11. Note that we have yet to investigate the theoretical convergence properties of the method.*

I think the practical considerations used to constrain the demonstration validation were basically reasonable, though I would prefer an "unlike" demonstration in addition to the "alike" demonstration used.

Further investigating the convergence properties of the calibration method is a good idea. We plan to validate the method further in a future study. However, the current paper is already very long and dense in material and for this reason we would like to conclude with the evidence that we have given at this point.

However, I'm not sure I agree that the method was actually showing convergence to the input values - some of the intervals, like Oslo R2, are definitely there, but others, like Oslo R1, definitely *aren't*. The authors need to grapple with that outcome a bit more frankly.

We have rephrased the paragraph to

“As shown in Figure S43 and Figure S44, both the presplit and postsplit stages converged asymptotically to the ground truth values for most parameters. One exception is R1 in Oslo, where the method seems to converge, but to a number which is somewhat higher than the ground truth. Note, however, that we are calibrating to one single realisation of the stochastic disease spread, and hence it might be that the underlying truth is not the best fitting parameter”.

On suitability:

I think the case that a regionally disaggregated approach is approach is preferrable is very clear, and I understand the comparison between national / regional forecasts. However, I do not think I was sufficiently clear about comparison to simpler methods in my original notes. The authors should just use one of the packaged non-parametric Rt estimation / incidence forecasting tools on some of the regional / national data and compete those estimates with the estimates from their more detailed models. I'm emphasizing "some" here, because I agree that data restrictions (e.g. too low hospitalization counts) may make those tools unusable for some regions. But it seems entirely reasonable to run one of the packaged Rt methods against the hospitalization data for e.g. Viken and Oslo and score that kind of non-parametric estimate against this approach (as well as evaluating computational resources etc to achieve the forecast). For the particular questions this work intends to address, the non-parametric Rt approach is more like a standard analysis, and so the authors should be characterizing their approach in terms of what it does better (e.g. probably dealing with the low-count regions) vs worse than the packaged Rt approaches.

We have added a comparison in the supplementary text section S2.9, where EpiNow2 is compared to our estimates for Oslo and Viken (the largest counties). For EpiNow2, we use the number of positive cases, as it is not straightforward to use both the hospitalisation and test data. The same changepoints in transmissibility are used.

The methods show the same general patterns. There are clear discrepancies in the early part of the pandemic, likely because the test data are not easy to use for this period, due to unstable testing capacity and criteria. For Oslo, our estimates are generally higher than for EpiNow2, likely due to the mobility, i.e. outflow of infectious individuals from Oslo. After the reopening of the borders in August 2020, we note that the EpiNow2 estimate is larger for both Oslo and Viken, presumably due to a high number of imported cases, which is information fed into our model estimates, and not to the EpiNow2 method.

On data availability:

I appreciate the sharing constraints and the easy way to satisfy them, but don't find them particularly tolerable as a reader. If the non-sensitive data are the only result that's publicly available, then a version of this analysis that is based on those data is what is appropriate *for the scientific literature* (reports for public health decision-making can be made to alternative standard). I'll also highlight again that asking readers to sift the raw public data into the format used in the analysis isn't appropriate, and furthermore it seems that resource may not have suitable longevity based on the README at <https://github.com/folkehelseinstituttet/surveillance_data>. The authors should figure out how to incorporate the relevant snapshot of that data as used in their analysis, in a more stable repository.

I also do not find the mobility data approach convincing. The example data is imprecisely documented (README indicates a Norway_adj data object, running a run_Norway.R script - that's presumably referring to data/dNorway.rds and test/run_Norway_test.R). The mob_data field in that object seems to be movement counts; the manuscript describes censoring counts < 20, but there are counts <20 in that data (e.g. mob_data[[1]] seems to be about 30% n < 20).

In the end, I'd refer to the PLOS data policy - "you make available all data used to draw the conclusions in the manuscript". If there are data that you'd like to use to drive some result, but can't make available, then you can't use them. If that means having to figure out a deindentified version of the data and then doing a lower resolution version of the analysis or whatever, well, then those the results that can be presented in the open scientific literature. I understand there are exceptions, but I'm not convinced that this analysis couldn't be done in a way more amenable to releasing data.

We have changed the data availability statement in accordance with the suggestion from the editor.

On assorted minor comments:

I found these responses mostly satisfactory.

I will clarify my question: "Regarding preferential returns of vistors to their home location: how does that comport with the disaggregated mobility data indications?" The model description says individuals are assigned a home location, i. They then visit j in some time period. In the next time period, any trips from j to i are preferentially assigned to people with home location i, currently visiting j (and then assorted other priority rules). My question concerned how well the individual data matched that assumption - from your reply, I gather you don't actually have access to the data in a way that would allow you to make that assessment. Is it possible to put that question to Telenor?

Regarding the preferential returning to home location, this has been studied before and is well documented (see for example González MC, Hidalgo CA, Barabási AL. 2008 Understanding individual human mobility patterns. Nature 453, 779-782. (doi:10.1038/nature06958 and Song C, Koren T, Wang P, Barabási AL. 2010 Modelling the scaling properties of human mobility. Nat. Phys. 6, 818-823. (doi:10.1038/nphys1760)).

The other assumptions are less evidence-based, but we do provide a sensitivity analysis to the mobility rules in the supplement, where the overall conclusion is that the statistics are relatively robust to the mobility assumptions.

It is correct that we do not have access to such individual-level data, and hence we cannot assess the degree to which the individual-level data match the assumptions.

Regarding putting the question to Telenor. Telenor has so far not analysed these individual-level data from the perspective of shedding light over the rule of preferential returns of visitors to their home location. The individual-level data is very short-lived (this data is only kept in Telenor’s systems for 21 days and is then deleted) and no longer exist. This is due to both regulatory and privacy reasons, as this data is considered extremely sensitive. Despite the great importance of verifying this scientific question, this will most likely not trump the purpose of guarding such data for ensuring full security and privacy for Telenor’s customers.

Regarding SEIR changing movement behavior: to be clear, the assumption made is an equally ad hoc quantity (that probability of movement remains = 1). I recommend considering a different angle on this argument.

We agree and have added “still” to the sentence, so that it now reads:

Another option would have been to decrease the probability of travel for e.g. the infectious symptomatic, however we have chosen not to do this, as it would *still* require ad hoc assumptions on the size of the decreased travel probability.

For averaging, estimating a time-averaged reporting probability, rather then day-scaled ones, is fine, for the practical reasons the reply highlights. I still don't think it makes sense to feed the estimation procedure adulterated data (by applying a smoothing window). Probably doesn't make much of difference in terms of resulting estimates, but its not like its computationally cheaper because it doesn't change data resolution. So seems like no reason to do it?

If we do not use a 7-days-moving average, we would have to model day-of-the-week effects in the test data. There are clear day-of-the-week effects in the test data, with a higher proportion of positive tests on weekends, and lower proportion of positive tests on Mondays. Though it is in theory possible to include a more complicated observation model for the test data in the model, we found it more practical to apply the moving average.
